# Promoter reinforcement supports transcriptional resilience in drug-resistant cancer

**DOI:** 10.1038/s41594-026-01829-0

**Published:** 2026-07-06

**Authors:** Vasumathi Kameswaran, Sayantanee Paul, Daniel Le, Alissa D. Guarnaccia, Jonathan Hoover, Liang-Fu Chen, Thijs J. Hagenbeek, Minyi Shi, Luke Y. Zhao, Jessica M. Lund, Ana Xavier-Magalhães, Julia Lau, Marco De Simone, Yuxin Liang, Antonina Hafner, Anwesha Dey, Zora Modrusan, Bence Daniel

**Affiliations:** 1 Department of Proteomic and Genomic Technologies, Genentech Inc., South San Francisco, CA USA; 2 Department of Discovery Oncology, Genentech Inc., South San Francisco, CA USA

**Keywords:** Chromatin structure, Epigenetics, Cancer, Transcription, Epigenomics

## Abstract

In mammalian cells, gene-distal regulatory elements enable long-range gene regulation and support cell-type-specific transcriptional programs. This regulatory architecture is frequently perturbed in cancer, particularly when oncogenic transcription factors are targeted therapeutically. However, how cancer cells adapt under such selective pressure has remained poorly understood. Here we show that mesothelioma cells dependent on the oncogenic TEAD family of transcription factors acquire resistance to a pan-TEAD inhibitor. Such resistance is accompanied by a promoter-centric regulatory mechanism, a process we term promoter reinforcement, to sustain gene expression following TEAD inhibition. Using base-pair-resolution Micro Capture-C on a set of TEAD target genes, we find that regulatory element–promoter interactions are weakened or lost in resistant cells, even as promoter activity and gene expression recover in the context of partial epigenetic restoration. Mechanistically, resistance-induced transcription factors show promoter-biased localization and can increase promoter activity, whereas distal regulatory element function can become dispensable. Together, these findings identify promoter reinforcement as a locus-specific compensatory response that supports transcriptional resilience under TEAD inhibition, indicating promoter-associated vulnerabilities in drug-resistant cancer.

## Abstract (web summary)

Kameswaran et al. show that cancer cells can restore gene expression after TEAD inhibition by shifting control from enhancers to promoters, a process termed ‘promoter reinforcement’, enabling transcription despite disrupted promoter–enhancer communication.

## Main

Transcription factor (TF)-regulated gene expression is orchestrated by long-range regulatory elements (REs; that is, enhancers, silencers and insulators) in the noncoding genome that interact with target gene promoters via chromatin looping^1–3^. REs serve as critical ‘landing pads’ for sequence-specific TFs, coordinating gene expression in a temporal- and cell-type-specific, distance- and orientation-independent manner^1,4–7^. Dysregulation of RE–promoter (RE–P) connections can often lead to disease, including cancers^8–10^.

In cancer, RE–P dysregulation may be driven by genetic and epigenetic mechanisms. Genetic mechanisms include single-nucleotide variants, insertions and deletions, and structural variants, which shape three-dimensional (3D) genome organization at multiple scales, including A/B compartments, topologically associating domains and RE–P interactions^11–14^. Epigenetic mechanisms such as DNA methylation can affect RE–P communication and genome compartmentalization, thereby disrupting normal gene regulation and altering cancer progression^15–20^.

Our understanding of how cancer cells restructure their 3D genome architecture to support growth, respond to treatment and establish resistance remains limited. This is especially true at the level of RE–P contacts, which require high-resolution analysis. Micro Capture-C (MCC) offers a promising solution, providing base-pair resolution with respect to RE–P interactions^21–23^. Therefore, MCC can provide unprecedented insight into RE–P interactions when cancer cells respond and develop resistance to small-molecule drugs.

Cancer cells often depend on specific transcriptional regulators, becoming ‘addicted’ to TFs such as MYC, EWS-FLI1, TAL1 or TEAD that can facilitate long-distance RE–P interactions^24–28^. This creates a therapeutic opportunity to target TFs that exploit REs of the noncoding genome. The TEAD family (TEAD1–4) of TFs, the main downstream effectors of the Hippo signaling pathway, represent a group of regulators that can bind promoter-distal REs and are critical growth regulators in multiple cancers^29–32^.

Malignant pleural mesothelioma is an aggressive cancer arising from pleural mesothelial cells, which form the epithelial-like cell layer lining the pleura. Mutations of genes encoding upstream Hippo pathway regulators such as *LATS2* or *NF2* are frequently detected across pleural mesothelioma cells^33–35^, culminating in high nuclear accumulation of YAP/TAZ and hyperactive transcriptional activity through its interaction with TEAD TFs. Thus, pharmacological regulation of the Hippo pathway has therapeutic relevance to pleural mesothelioma and several other cancers, but how targeting this pathway will affect the regulatory landscape of these cells has remained unclear. Understanding the 3D genome organization and, specifically, RE–P interactions in TEAD-inhibited cancers could clarify the broad mechanistic effects involved, particularly in cases of acquired resistance.

Here we studied the mechanism by which two pleural mesothelioma cell lines (NCI-H226 and MSTO-211H, referred to as H226 and MSTO, respectively) with hyperactivation of Hippo signaling due to genetic deficiency of *NF2* and *LATS2*, respectively, develop resistance to the pan-TEAD inhibitor (TEADi) GNE-7883^26^. GNE-7883 works by binding to TEAD and displacing its cofactor YAP, leading to target gene repression and cell growth inhibition^36^.

We observed that TEADi-resistant mesothelioma cells established a gene expression and YAP occupancy profile akin to that of vehicle-treated parental cells with resistance-specific differences. Base-pair-resolution MCC and genome engineering experiments revealed that many pan-TEADi-sensitive genes in resistant cells do not depend on RE–P interactions for their transcriptional recovery. We found that TFs that gained activity in the resistant state showed promoter-biased localization and circumvented the lost RE–P interactions via enhanced promoter activity, a phenomenon that we term ‘promoter reinforcement’. Our results support a model in which drug-resistant cancer cells can sustain gene expression programs through promoter-driven mechanisms to overcome selective pressure from therapeutic agents and are consistent with mechanistic insights gained from high-resolution 3D genome architecture assays.

## Results

### Transcriptomic restoration is a hallmark of drug-resistant cancer cells

**Fig. 1:**
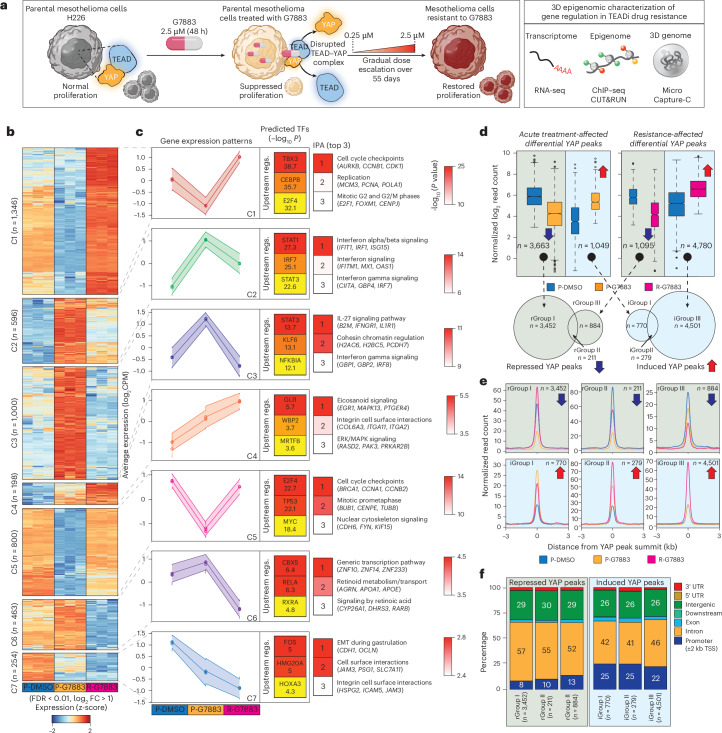
Transcriptomic and epigenomic restoration in drug-resistant cancer. **a**, Experimental workflow. Parental H226 mesothelioma cells were treated with the pan-TEADi GNE-7883 (G7883; 2.5 μM, 48 h), resulting in suppressed proliferation and disruption of the YAP–TEAD complex. Gradual dose escalation (0.25–2.5 μM over 55 days) generated resistant cells with restored proliferation^37^. Cells from parental, acutely treated and resistant states were profiled by RNA-seq, ChIP–seq, CUT&RUN and MCC. **b**, Heatmap of differentially expressed genes (FDR < 0.01, log_2_FC > 1, *n* = 3; *n* = 4,657) grouped by *k*-means clustering. **c**, Average gene expression profiles for each cluster (left). Top three upstream regulators predicted by IPA are shown with enrichment significance (–log_10_-transformed *P* values; right-tailed Fisher’s exact test, no additional multiple-testing correction) in the inner heatmaps (middle). The top three enriched biological pathways with representative genes are shown (right). **d**, Differential YAP binding across conditions (FDR < 0.01). Box plots show normalized read counts; boxes indicate the interquartile range (IQR), center lines the median, and whiskers extend to 1.5× IQR. Arrows denote decreased or increased YAP occupancy. Venn diagrams show overlap of peaks with decreased or increased occupancy, defining six binding groups. **e**, Aggregate profiles of normalized read enrichment centered on YAP peak summits for each binding group. **f**, Genomic annotation of YAP peaks across binding groups, shown as percentages of total peaks. Regs., regulators; UTR, untranslated region. Panel **a** created in BioRender; Daniel, B. https://biorender.com/gicmylv (2026). Source data

To define transcriptional changes associated with drug response and resistance, we performed bulk RNA sequencing (RNA-seq) on mesothelioma H226 cells under three scenarios: (1) parental cells treated with vehicle (dimethyl sulfoxide; P-DMSO), (2) parental cells acutely treated (48 h) with pan-TEADi GNE-7883 (P-G7883) and (3) cells made resistant to GNE-7883 (R-G7883) through continuous exposure to increasing concentrations of the inhibitor. Briefly, parental H226 cells were subjected to gradual dose escalation from 0.25 µM to 2.5 µM over 55 days, allowing recovery and outgrowth of persister populations under sustained drug pressure, as previously described^37^ (Methods and Fig. 1a).

Across all conditions, we detected 4,657 differentially expressed genes (false discovery rate (FDR) < 0.01, log_2_(fold change (FC)) > 1), which could be grouped according to seven distinct transcriptional patterns (C1–C7; Fig. 1b and Supplementary Table 1). These clusters defined distinct responses to acute treatment and resistance.

C1 and C5 included genes that were downregulated upon acute GNE-7883 treatment but whose expression either returned to the levels in parental cells (C5, *n* = 800) or exceeded them (C1, *n* = 1,346) in the resistant state. These clusters therefore reflected transcriptional recovery and overcompensation. Upstream regulator analysis using Ingenuity Pathway Analysis (IPA) predicted cell cycle TFs (for example, E2F and MYC) and enrichment of cell cycle pathways (for example, replication)^38,39^ (Fig. 1c and Supplementary Table 2). C2 and C3 included genes induced upon acute inhibitor treatment. In C2 (*n* = 596), expression remained elevated in resistant cells, whereas in C3 (*n* = 1,000), expression returned to baseline. These clusters therefore captured sustained versus transient induction and were associated with inflammatory TFs (for example, STATs and IRFs) and interferon-driven inflammatory pathways^40,41^.

Genes in C4, the smallest cluster (*n* = 198), showed de novo activation, with low or no expression in parental cells and progressive upregulation, with expression peaking in resistant cells. Predicted regulators included TFs involved in proliferation and differentiation (for example, GLI1 and WBP2), with enrichment of integrin cell surface interactions and ERK/MAPK signaling^42,43^. C6 and C7 showed reduced expression compared to parental cells. In C6 (*n* = 463), repression was specific to resistant cells, whereas in C7 (*n* = 254), repression occurred upon acute treatment and was further enhanced in resistant cells. These clusters thus represented progressive repression across conditions and included predicted regulators of CBX5, RXRA, FOS and HOX, as well as SNAI2, which is a regulator of integrin and cadherin genes and of epithelial–mesenchymal transition, the top pathway in C7^44^.

**Extended Data Fig. 1:**
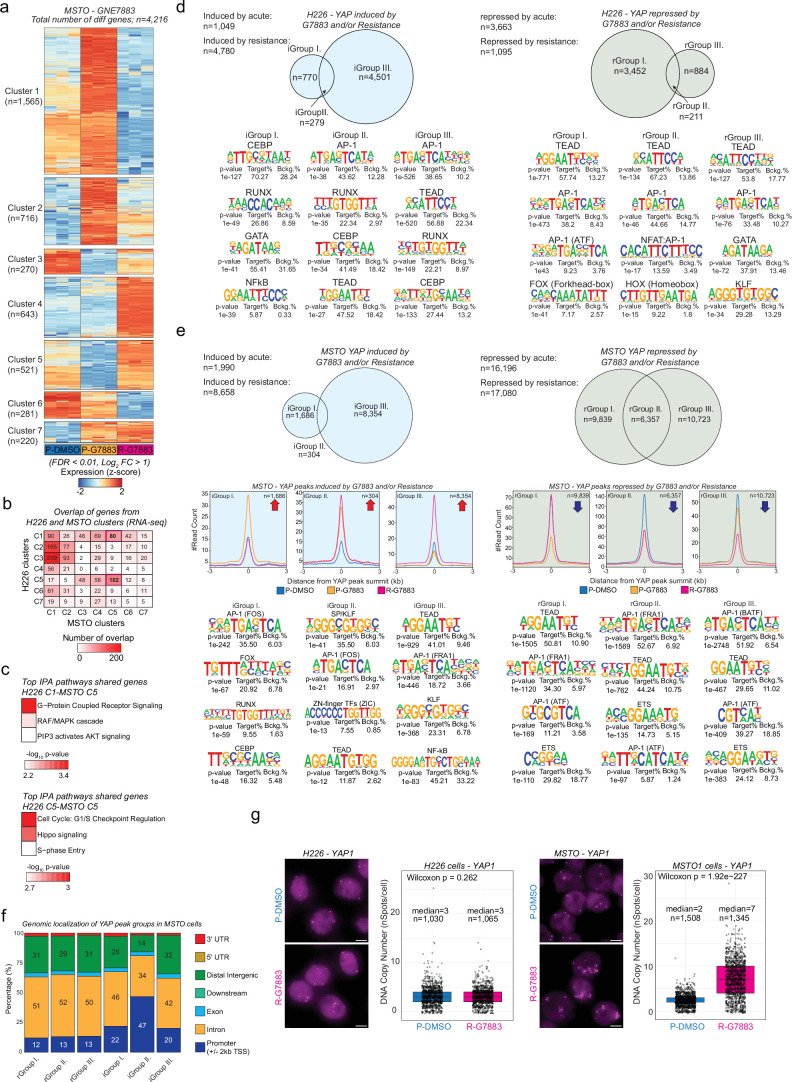
Transcriptomic and YAP cistromic analyses in MSTO and H226 mesothelioma cells. (**a**) Heatmap showing differentially expressed genes (FDR < 0.01, log_2_FC > 1; n = 4,216) in MSTO cells treated with DMSO, acute GNE-7883, and resistant conditions, clustered into seven expression groups (C1-C7). (**b**) Heatmap displaying overlap analysis between transcriptional clusters derived from MSTO and H226 RNA-seq datasets. (**c**) Top three canonical pathways from Ingenuity Pathway Analysis (IPA) for overlapping gene clusters: shared pathways between H226 C1 and MSTO C5 (top) and between H226 C5 and MSTO C5 (bottom). Overlap of YAP ChIP-seq peaks gained or decreased by acute GNE-7883 treatment and resistance in H226 (**d**) or MSTO cells (**e**), shown as Venn diagrams. Enriched transcription factor motifs identified within each group are displayed below. Below, read coverage plots across YAP peak summits and corresponding motif logos for lost YAP peaks. (**f**) Genomic feature annotation of YAP peak groups in MSTO cells, represented as stacked bar plots showing the distribution of peaks across promoters ( ± 2 kb TSS), intronic, exonic, and intergenic regions. (**g**) DNA FISH analysis of YAP1 copy number in parental and GNE-7883–resistant H226 and MSTO1 cells. Representative images (top) and per-cell spot quantification (bottom) show no change in YAP1 copy number in H226 (median = 3), whereas MSTO1-resistant cells display a significant increase in YAP1 copy number compared to parental cells (scale bar - 5 µm; median = 7; Wilcoxon rank sum test, p < 0.001). Source data

To assess conservation of these patterns, we performed RNA-seq in a second mesothelioma cell model (MSTO; Methods), identifying 7 clusters (differential genes; *n* = 4,216) across the same conditions (P-DMSO, P-G7883 and R-G7883) (Extended Data Fig. 1a and Supplementary Table 3). We then compared clusters across models to identify shared programs. Overlap analysis focused on genes that recovered after acute treatment or gained activity in resistant cells (Extended Data Fig. 1b). Clusters with shared programs included H226 C1 and MSTO C5 (*n* = 80), which showed transient repression followed by strong recovery or increased expression in resistance; and H226 C5 and MSTO C5 (*n* = 102), which showed full recovery after acute repression. These conserved programs indicate common recovery mechanisms and were enriched for RAF/MAPK, cell cycle and Hippo signaling (Extended Data Fig. 1c).

Together, these data define transcriptional responses to acute pan-TEADi treatment and resistance. Resistant cells adopt a distinct state dominated by restoration toward a parental-like transcriptional program, with a smaller contribution from de novo gene activation.

### Restoration and extension of the YAP cistrome in drug resistance

GNE-7883 functions by displacing YAP, a key cofactor of TEAD, from chromatin-bound TEAD^26^. To define how YAP chromatin binding changed during acute treatment and resistance, we performed YAP chromatin immunoprecipitation followed by sequencing (ChIP–seq) and differential binding analysis in both models (FDR < 0.01, log_2_FC > 1), comparing (1) P-DMSO versus P-G7883 and (2) P-DMSO versus R-G7883. These analyses identified widespread remodeling of YAP occupancy in both acute treatment and resistance conditions. Acute treatment resulted in peaks with decreased and increased YAP occupancy (H226 decreased: *n* = 3,663; increased: *n* = 1,049; MSTO decreased: *n* = 16,196; increased: *n* = 1,990), whereas resistance showed a distinct pattern with substantial gain of YAP binding sites (H226 decreased: *n* = 1,095; increased: *n* = 4,780; MSTO decreased: *n* = 17,080; increased: *n* = 8,658; Fig. 1d, top, and Extended Data Fig. 1d,e).

To define shared and condition-specific binding events, we overlapped peaks with increased YAP occupancy (iGroups I–III) and peaks with decreased YAP occupancy (rGroups I–III) across the two comparisons within each model (Fig. 1d, bottom, and Extended Data Fig. 1d,e). This classification distinguished peaks affected by acute treatment (rGroup I and iGroup I), resistance (rGroup III and iGroup III) or both (rGroup II and iGroup II). In MSTO cells, rGroup I peaks (*n* = 9,839; Extended Data Fig. 1e) showed decreased YAP occupancy upon acute treatment, followed by restoration to parental levels in resistant cells. By contrast, H226 rGroup I peaks (*n* = 3,452, Fig. 1e) exhibited restoration and a further increase in YAP occupancy in resistance, whereas rGroups II (H226: *n* = 211; MSTO: *n* = 6,357) and III (H226: *n* = 884; MSTO: *n* = 10,723) showed reduced YAP occupancy upon both acute treatment and resistance, and all iGroups displayed elevated YAP occupancy relative to control parental cells (Fig.1e and Extended Data Fig. 1e).

Motif analysis of peaks with increased YAP occupancy (iGroups I–III) revealed enrichment for CEBP, AP-1, NF-κB and RUNX TF motifs, whereas peaks with decreased YAP occupancy (rGroups I–III) were enriched for TEAD and AP-1 TF motifs in both models (Extended Data Fig. 1d,e). Peaks with increased YAP occupancy, either upon acute treatment or in resistance, were approximately twice as likely as peaks with decreased occupancy to localize to promoter proximal regions (±2 kb from transcription start sites (TSSs); Fig. 1f and Extended Data Fig. 1f), indicating a shift toward promoter-associated YAP binding in resistance.

To determine whether restoration of YAP occupancy in resistant cells was driven by changes in gene dosage, we assessed *YAP1* copy number by DNA fluorescence in situ hybridization (FISH). No change in *YAP1* copy number was observed in resistant H226 cells despite expansion of the YAP1 cistrome, whereas resistant MSTO cells exhibited an approximately three-fold increase in *YAP1* copy number (Extended Data Fig. 1g).

Together, these results demonstrate that both models restore and expand the YAP cistrome with a promoter-biased binding profile in resistance, independent of *YAP1* copy number status.

### Dysregulated RE–P interaction networks in resistant cells

**Fig. 2:**
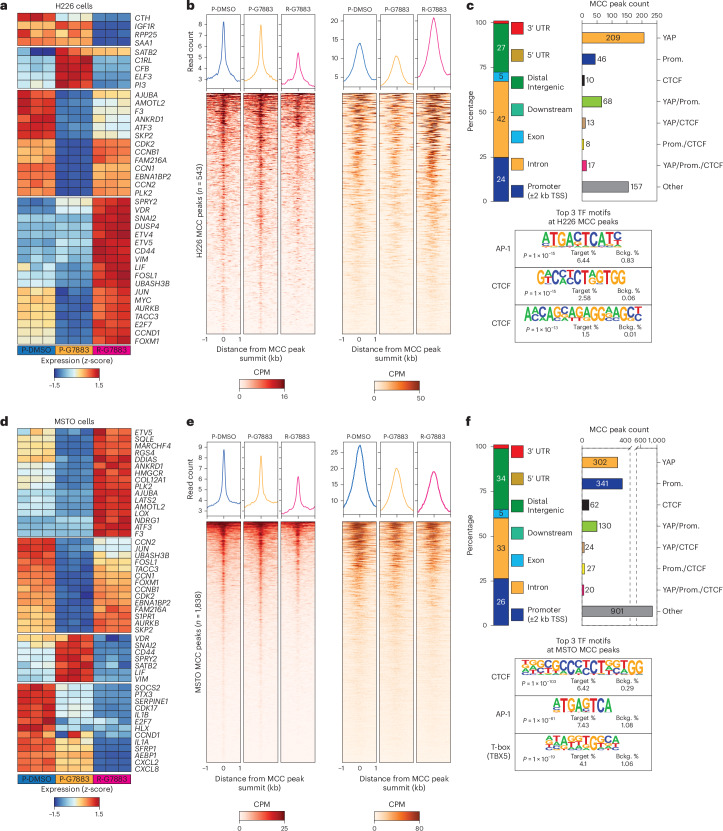
RE–P interactions are disrupted in resistant cancer cells. **a**, Heatmap of gene expression (*z*-scores) for MCC-selected genes in H226 cells (*n* = 40) across parental (P-DMSO), acutely TEAD-inhibited (P-G7883) and resistant (R-G7883) conditions. **b**, Aggregated read-density plots and heatmaps showing normalized MCC contacts (left) and YAP ChIP–seq signal (right) centered on MCC peak summits (*n* = 543). **c**, Genomic annotation of H226 MCC peaks and their overlap with YAP, CTCF and promoter sites; ‘other’ indicates overlap with ATAC-seq peaks only (top). Motif enrichment is also shown (bottom). **d**, Heatmap of MCC target gene expression (*z*-scores) in MSTO cells (*n* = 50) across the same conditions. **e**, Aggregate MCC and YAP read-density profiles at MSTO MCC peaks (*n* = 1,838). **f**, Genomic annotation and motif enrichment of MSTO MCC peaks as in **c**. Motif enrichment was performed using HOMER (hypergeometric test, one-sided; no additional multiple-testing correction). Bckg., background; prom., promoter. Source data

**Extended Data Fig. 2:**
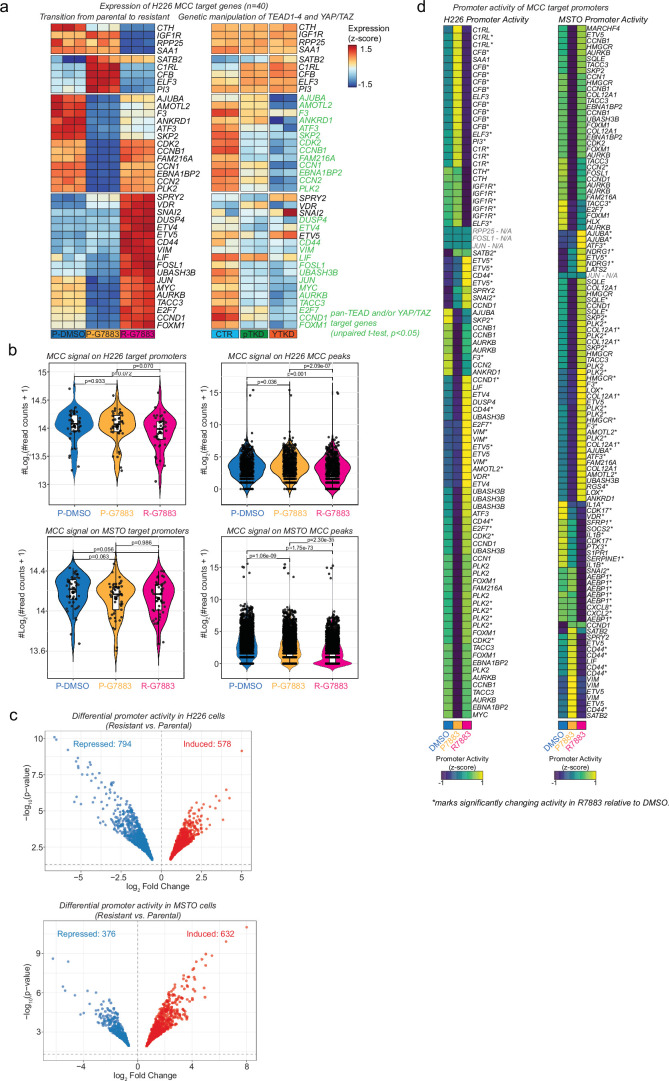
Promoter activity and MCC-based interaction analysis in H226 and MSTO cells. (**a**) Heatmap of H226 MCC target gene expression (RNA-Seq, z-score; left). Right, heatmap of the same genes after CRISPR-mediated perturbation of YAP/TAZ or pan-TEAD in parental cells to assess their dependence on YAP/TAZ-TEAD signaling. Genes highlighted in green are pan-TEAD and/or YAP/TAZ targets (unpaired t-test, p < 0.05) (**b**) Violin plots of normalized MCC read coverage at target promoters for H226 (top) and MSTO (bottom) cells across experimental conditions. Right, violin plots displaying MCC read coverage at corresponding distal regulatory elements (REs). Statistical significance was assessed using the Wilcoxon signed-rank test (p < 0.05). (**c**) Volcano plots of differential promoter activity between parental and resistant conditions. Statistically significant promoter activity changes were defined by Local False Sign Rate (LFSR) ≤ 0.1. (**d**) Heatmaps of promoter activity for MCC-targeted genes in H226 and MSTO cells across all conditions. Stars mark statistically significant promoter activity changes (LFSR ≤ 0.1). Alternative promoters are also displayed. Source data

Having established that transcriptional programs and YAP binding were restored or extended in resistant cells, we next considered whether RE–P interactions were similarly affected. To address this, we performed high-resolution genome conformation mapping via MCC in both models and designed model-specific hybrid capture probes targeting promoters of TEAD target genes validated by *YAP/TAZ* or pan-TEAD (*TEAD1–4*) perturbations in parental H226 cells (Extended Data Fig. 2a, right). Capture probes included promoters of genes showing restored or increased expression in resistant cells, as well as control genes with reduced expression in resistance (H226 *n* = 40 and MSTO *n* = 50; Fig. 2a,d and Supplementary Table 4).

First, we examined read coverage at targeted gene promoters and observed similar signals across conditions, indicating comparable capture efficiency (Extended Data Fig. 2b). We next analyzed read coverage at REs interacting with the captured promoters that overlapped with open chromatin regions defined by assay for transposase-accessible chromatin using sequencing (ATAC-seq) (peak calling, Methods). This identified 543 RE peaks interacting with 40 promoters in H226 cells and 1,838 peaks interacting with 50 gene promoters in MSTO cells. Despite similar promoter capture efficiency, the average MCC signal at these interacting REs was comparable between parental and acutely treated cells but significantly reduced in the resistant state in both models (Wilcoxon rank-sum test, *P* < 0.05; Fig. 2b,e and Extended Data Fig. 2b). However, YAP binding dynamics at these REs differed between models. In H226 resistant cells, YAP occupancy was increased relative to parental cells, whereas in MSTO cells, YAP occupancy remained reduced in the resistant state compared to parental cells (Fig. 2b,e).

Annotation of MCC peaks showed that captured promoters primarily interacted with YAP-bound regions, gene promoters, CTCF sites and other open chromatin regions defined by ATAC-seq (Fig. 2c,f, top). Motif enrichment analysis revealed AP-1 and CTCF motifs in both models and a T-box motif specifically in MSTO cells (Fig. 2c,f, bottom). To determine whether these interaction changes were accompanied by altered promoter activity, we quantified promoter activity using bulk RNA-seq-based inference of promoter-initiated transcript levels^45^. Across the genome, we identified promoters with differential activity between parental and resistant cells, including 794 with decreased activity and 578 with increased activity in resistant H226 cells, and 376 with decreased activity and 632 with increased activity in resistant MSTO cells (local false sign rate ≤ 0.10) (Extended Data Fig. 2c and Supplementary Table 5). Among MCC-targeted promoters, 13 showed increased activity in resistant H226 cells (for example, *VIM*, *CD44*, *SKP2*), and 10 showed increased activity in resistant MSTO cells (for example, *TACC3*, *FOSL1*, *RGS4*) (Extended Data Fig. 2d), whereas most promoters exhibited full or partial recovery of activity in both models.

Together, these results indicate that reduced RE–P interaction strength coincides with restored or increased promoter activity at selected loci, consistent with a shift in regulatory logic in resistant cells toward reduced dependence on RE–P interactions for gene expression recovery.

### Enhancers become dispensable for transcriptional regulation in resistance

To test whether distal REs that lost promoter interactions in the resistant state remained enhancers of gene expression, we used paired CRISPR guides to delete selected REs and promoters in both cell line models. Target regions were chosen based on loss or reduction of RE–P interactions detected by MCC in resistant cells. Using this strategy, we perturbed REs or promoters at the *FOSL1* and *TACC3* loci in both models; *CCN1*, *CDK2* and *ANKRD1* in H226; and *RGS4* and *PRCP*-associated REs in MSTO, with the expectation that RE ablation would show diminished effects on gene expression in resistant cells.

**Fig. 3:**
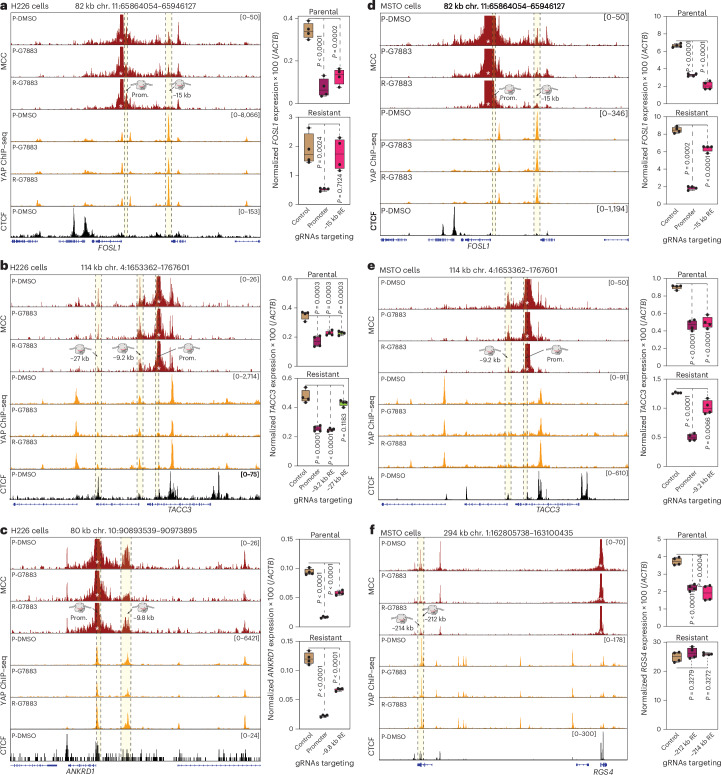
Distal RE activity is dispensable in resistant cells. **a**–**c**, Genome browser tracks visualizing MCC, YAP and CTCF ChIP–seq signals in the indicated conditions at the *FOSL1* (**a**), *TACC3* (**b**) and *ANKRD1* (**c**) loci from H226 cells. **d**–**f**, Genome browser tracks visualizing MCC, YAP and CTCF CUT&Tag^53^ signals in the indicated conditions at the *FOSL1* (**d**), *TACC3* (**e**) and *RGS4* (**f**) loci from MSTO cells. CRISPR perturbation loci are highlighted by yellow shaded regions, and viewpoint regions are represented by asterisks. Box plots represent *ACTB*-normalized mRNA levels for genes in the indicated conditions following CRISPR deletion. The box represents the IQR, the internal line represents the average of the data, and whiskers extend to the smallest and largest values. Significant differences were determined by two-tailed, unpaired *t*-tests at *P* < 0.05; *n* = 4 (two biological and two technical replicates were performed for each editing experiment). Source data

**Extended Data Fig. 3:**
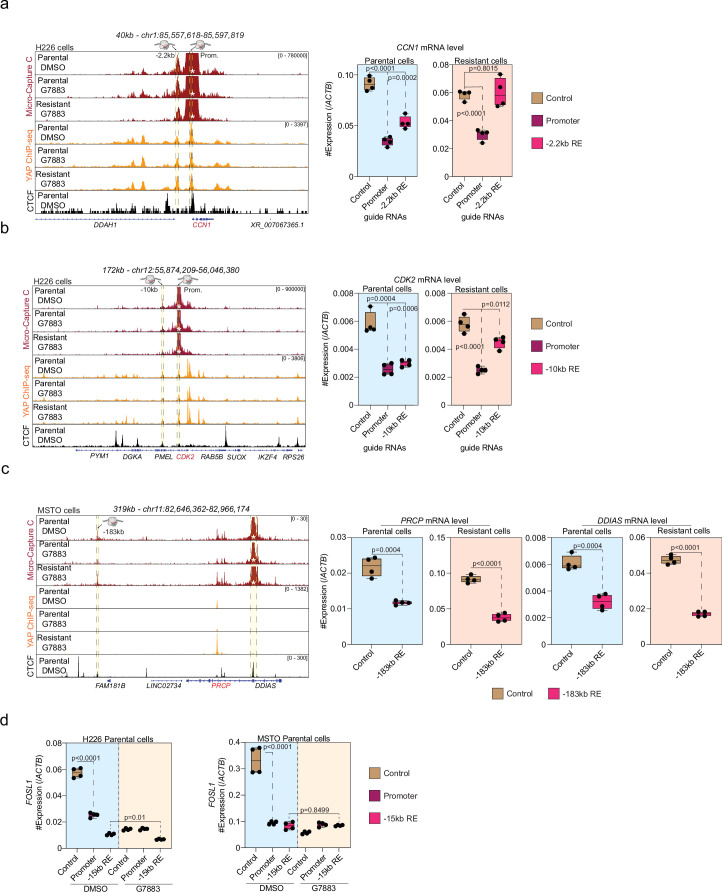
CRISPR-mediated perturbation of promoters and distal regulatory elements in H226 and MSTO cells. (**a**-**c**) Genome browser snapshots of the regions targeted for CRISPR excision at select promoters and/or distal regulatory elements (REs) in H226 (**a**, **b**) and MSTO (**c**) cells. Yellow shaded regions indicate genomic segments removed using pairs of guide RNAs in parental (DMSO) and resistant (R-G7883) conditions. Boxplots show RT-qPCR-based *ACTB*-normalized (#) mRNA expression levels of the indicated genes following gene editing in parental and resistant cells. Box represents the interquartile range (IQR), the internal line indicates the average, and whiskers span the minimum to maximum values. Each boxplot represents n = 4 measurements (2 biological and 2 technical replicates). Significant differences were determined using two-tailed unpaired *t*-tests (p < 0.05). (**d**) CRISPR-mediated perturbation of the promoter and a distal RE upstream of *FOSL1* in parental and acutely GNE-7883-treated H226 and MSTO cells. Boxplots show *ACTB*-normalized (#) *FOSL1* expression measured by RT-qPCR under the indicated conditions, with the same boxplot structure and sample size as above. Statistical significance was assessed using two-tailed unpaired *t*-tests (p < 0.05). Source data

At the *FOSL1* locus, we targeted a −15-kb RE that interacted with the promoter in parental cells but lost this interaction in resistance, despite restored YAP binding in H226 and reduced YAP binding in MSTO (Fig. 3a,d). Deletion of this RE reduced *FOSL1* expression in both parental models but had only marginal effects in resistant cells, whereas promoter perturbation reduced expression in both parental and resistant states. Similar behavior was observed at the *CCN1* and *CDK2* loci in H226 cells (Extended Data Fig. 3a,b): in parental cells, *CCN1* depended on a −2.2-kb RE (overlapping with the *DDAH1* promoter) and *CDK2* on an intronic −10-kb RE, but these dependencies were diminished or lost in resistance.

We next examined the *TACC3* locus, which showed reduced RE–P interactions in both models but distinct architectures. In H226, we perturbed two REs: a −27 kb CTCF-overlapping site that lost interaction in resistant cells and a −9.2 kb site (the *SLBP* promoter) that retained partial interaction (Fig. 3b). All perturbations reduced *TACC3* expression in parental cells. In resistant H226 cells, the −27-kb RE became dispensable, whereas the −9.2-kb RE remained essential. In MSTO cells, in which no strong −27-kb interaction was detected, we targeted only the −9.2-kb RE and the promoter; although this RE retained function in resistant H226 cells, it became dispensable in resistant MSTO cells (Fig. 3e). Further, at the *RGS4* locus in MSTO, two long-range REs (−214 kb CTCF and −212 kb YAP–CTCF co-occupied sites) were required for expression in parental cells but became dispensable in resistance (Fig. 3f).

We also analyzed loci in which RE–P interactions were reduced after acute treatment but exhibited signs of restoration in resistance. In H226 cells, perturbation of an intergenic −9.8-kb RE at the *ANKRD1* locus reduced expression in both parental and resistant cells, as did perturbation of the promoter (Fig. 3c). Similarly, in MSTO cells, deletion of a −183 kb CTCF site interacting with the ‘shared’ promoter of *PRCP* and *DDIAS* reduced expression of both genes in parental and resistant states (Extended Data Fig. 3c).

Finally, to assess residual enhancer activity after acute treatment, we perturbed the *FOSL1* promoter and −15-kb RE under acute GNE-7883 exposure. Although acute treatment reduced *FOSL1* expression, promoter deletion did not further decrease expression in either model. Deletion of the −15-kb RE caused a small additional decrease in H226 but had no effect in MSTO (Extended Data Fig. 3d).

Together, these perturbation experiments demonstrate that multiple genes recover or maintain expression in resistant cells despite reduced distal RE function, supporting a shift toward promoter-reinforced regulatory control.

### Resistance-induced FOSL1 exhibits promoter-biased binding profile in the genome

**Extended Data Fig. 4:**
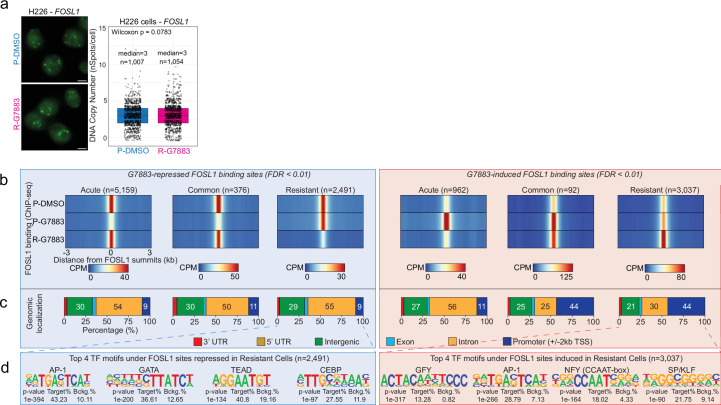
FOSL1 exhibits promoter-biased localization in resistant H226 cells. (**a**) DNA FISH analysis of *FOSL1* in parental and GNE-7883–resistant H226 cells. Representative images (left, scale bar 5μm) and quantification of DNA copy number per cell (right) show no significant change between parental and resistant states (median = 3 copies per cell; Wilcoxon rank sum test, p < 0.001). (**b**) Heatmap representation of normalized FOSL1 ChIP-seq signal around lost (blue box), and gained (pink box) FOSL1 peaks. Regions lost or gained specifically in the acute treatment (‘Acute’), resistant state (‘Resistant’) or both (‘Common’) are represented. (**c**) Stacked bar plots represent the genomic localization of FOSL1 binding sites across the different peak groups. (**d**) Top 4 enriched transcription factor motifs in the ‘Resistant’ state for either GNE7883-specific lost (Left, Blue box) or -gained (Right, Pink box) FOSL1 peaks are shown. Each motif logo is visualized with the enrichment *p*-value, the percentage of motif presence in the target peaks (Target%), and their presence in ~50k random background genomic sequences (Bckg.%). Source data

To identify gene regulatory mechanisms by which GNE-7883-resistant cells might circumvent the observed loss of RE–P interactions, we focused on TFs that gained activity in the resistant state of H226 cells. Recent research has identified AP-1 family member FOSL1 as a critical TF in the resistant state that is essential for maintaining cell proliferation^37^. First, we assessed copy number variation with DNA FISH in parental and resistant cells and did not observe copy number differences for *FOSL1* (Extended Data Fig. 4a). Second, we performed ChIP–seq for FOSL1 to understand how the FOSL1 cistrome was remodeled in resistant cells. This analysis identified FOSL1 binding sites that were specific to acute inhibitor treatment (‘acute’), resistance (‘resistant’) or both (‘common’) (FDR < 0.01, log_2_FC > 1; Extended Data Fig. 4b).

Notably, FOSL1 sites that were gained in resistant cells (*n* = 3,037) or by acute treatment and maintained in the resistant state (*n* = 92) were more enriched in the vicinity of gene promoters than any other FOSL1 peak group (for example, 44% in ‘common’ and ‘resistant’ versus 11% ‘acute’; Extended Data Fig. 4c). Further analysis of the resistance-specific gained FOSL1 sites revealed strong enrichment of promoter-specific TF motifs (for example, GFY, NFY, SP/KLF), whereas FOSL1 sites decreased in resistance were enriched for AP-1 and TEAD motifs (Extended Data Fig. 4d).

These findings demonstrate that resistance-induced FOSL1 exhibits a promoter-biased binding profile and suggests that some of the resistance-specific TFs might preferentially operate from gene promoters to recover gene expression in resistance.

### TF network of TEADi-resistant mesothelioma cells

**Extended Data Fig. 5:**
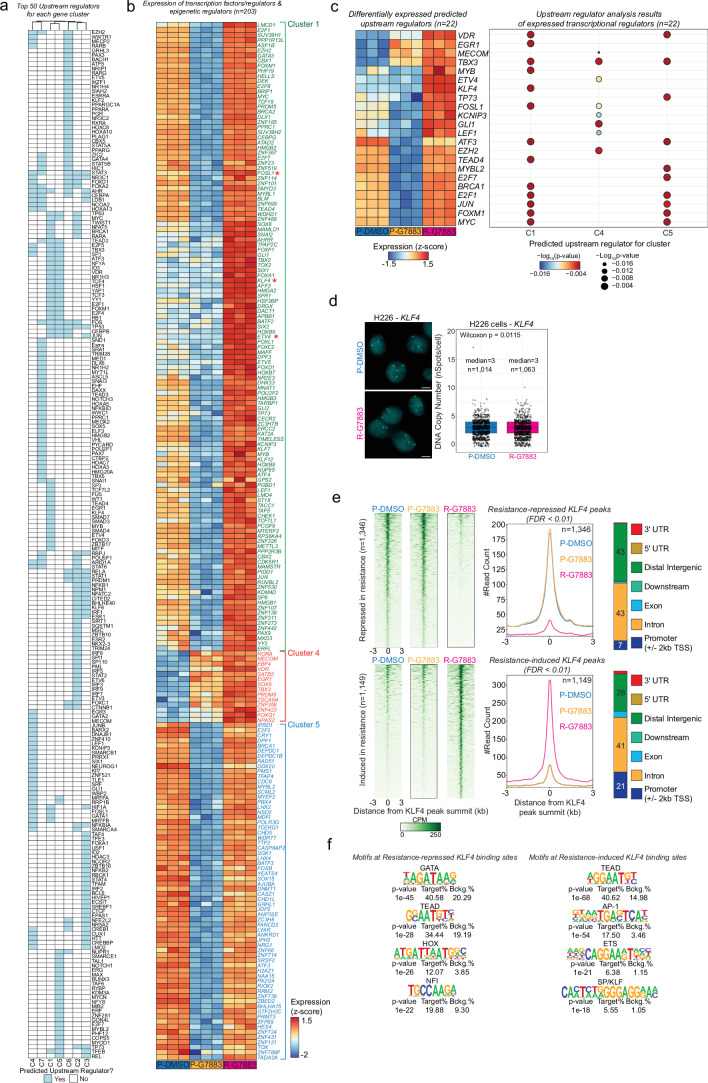
Integrated identification of transcriptional regulators and KLF4 cistromic remodeling in resistant H226 cells. (**a**) Table of the top predicted upstream regulators for each transcriptional cluster derived from H226 RNA-seq data, as identified by Ingenuity Pathway Analysis (IPA). (**b**) Heatmap of expression (z-score) of transcription factors, regulators, and epigenetic modifiers (n = 203) identified from the TFCheckpoint database that are differentially expressed in H226 cells. Regulators belonging to RNA-seq Clusters 1, 4, and 5 are indicated. (**c**) Heatmap of transcriptional regulators that were predicted by IPA upstream regulator analysis and whose expression was restored or increased in the resistant state. Right, dot plot summarizing IPA upstream regulator analysis results for the same factors; dot color and size represent -log_10_ enrichment *p*-values. (**d**) DNA FISH analysis of *KLF4* in parental and GNE-7883–resistant H226 cells. Representative images (left, scale bar 5 µm) and quantification of DNA copy number per cell (right) show no significant change in *KLF4* copy number between parental and resistant states (median = 3 copies per cell; Wilcoxon rank sum test, p < 0.001). (**e**) Differential KLF4 ChIP-seq binding analysis in resistant versus parental cells (FDR < 0.01). Heatmaps display normalized read density at KLF4 binding sites that are significantly diminished (top) or increased (bottom) in resistance. Histograms to the right show corresponding average KLF4 signal profiles centered on peak summits. The stacked bar plot illustrates the genomic annotation of lost and gained KLF4 peaks, including promoters ( ± 2 kb TSS), intronic, exonic, downstream, and intergenic regions. (**f**) Enriched transcription factor motifs identified at KLF4 peaks that are decreased (left) or increased (right) in resistant cells. Motif logos are shown with associated statistical values (p-value, target count, and background frequency). Source data

We then used Ingenuity’s upstream regulator analysis to predict TFs that could regulate gene expression in resistant H226 cells. Using genes that were specifically induced, or restored in the resistant state, IPA upstream regulator analysis identified TFs whose known targets were significantly enriched among these gene sets (Extended Data Fig. 5a). Next, to identify the most relevant TFs that governed the resistant state, we used TFCheckpoint^46^, a database of transcriptional regulators, and overlaid its members with genes that exhibited resistance-restored or resistance-induced expression compared to parental control cells (RNA-seq C1, C4 and C5; Fig. 1b). This analysis identified 203 potential transcriptional regulators (Extended Data Fig. 5b and Supplementary Table 6). By taking the intersection of the predicted upstream regulators with this TF gene list, we identified 22 TFs with potential roles in governing resistance-specific gene expression, including *FOSL1*^37^, *KLF4* and *ETV4* (Extended Data Fig. 5c).

The above results and the fact that KLF TFs could bind the promoter-specific SP/KLF motif (that is, the GC-box), motivated us to further evaluate the roles of KLF4 in resistance. We first determined *KLF4* copy numbers and found no difference between parental and resistant cells by DNA FISH (Extended Data Fig. 5d). Next, we mapped the binding sites of KLF4 by ChIP–seq with the expectation that KLF4 would show a largely resistance-specific binding profile based on its expression pattern; nonetheless, we detected a similar number of KLF4 peaks that were lost (*n* = 1,345) and gained (*n* = 1,149) in resistance (FDR < 0.01). No acute GNE-7883 treatment induced changes were detected. Notably, resistance-specific KLF4 peaks exhibited promoter-biased binding profiles compared to the ones that were weaker in the resistant state (decreased 7% versus gained 21%), with enrichment for the promoter-specific SP/KLF motif; however, most KLF4 peaks were found at intronic and intergenic locations (Extended Data Fig. 5e,f).

Overall, these data identify a TF network of resistance and indicate that TFs including KLF4 are potential regulators of the resistant state in H226 cells.

### Promoter-centered regulation accompanies transcriptional recovery in resistance

**Fig. 4:**
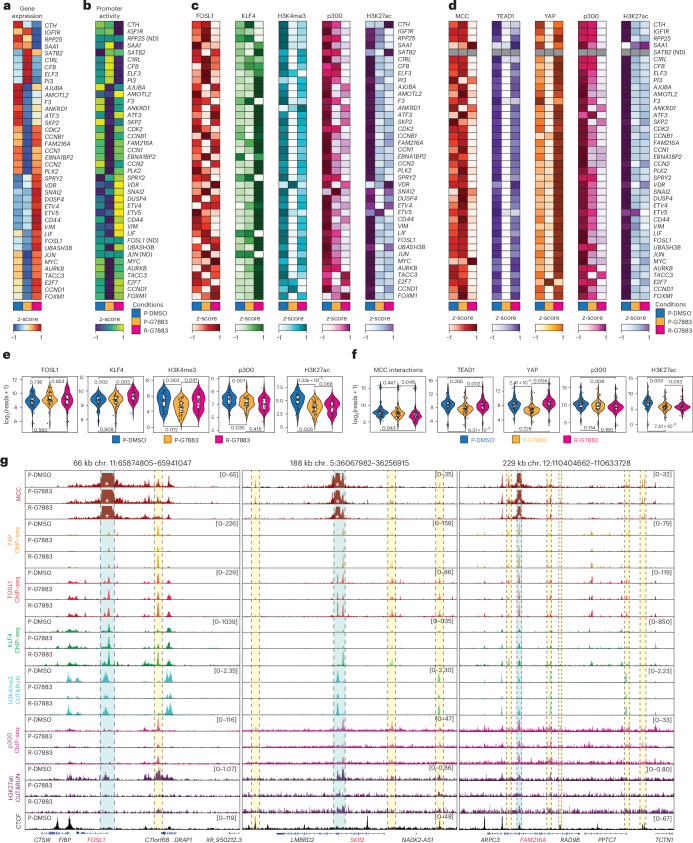
Promoter activity and gene expression are restored in a dysfunctional chromatin conformation and HAT activity context. **a**,**b**, Heatmaps of RNA expression (**a**) and inferred promoter activity (**b**; from bulk RNA-seq^45^) for MCC target genes (*n* = 40) across P-DMSO, P-G7883 and R-G7883 H226 cells (*z*-score). **c**,**d**, Heatmaps of CPM-normalized signal (*z*-score) at (**c**) MCC-targeted promoters for FOSL1, KLF4, H3K4me3, p300 and H3K27ac and (**d**) MCC-linked distal REs for MCC interaction frequency, TEAD1, YAP, p300 and H3K27ac occupancy (ChIP–seq or CUT&RUN). **e**,**f**, Violin plots of normalized read counts at promoters (**e**) and REs (**f**). Center lines indicate medians, distributions reflect data density and overlaid boxes show IQR with whiskers extending to 1.5× IQR. Statistical significance was assessed using a two-sided Wilcoxon rank-sum test (*P* < 0.05; *n* = 2 biological replicates). **g**, Genome browser tracks of representative loci. Blue shading marks promoters (asterisks); yellow shading marks MCC-linked REs. ND, not detected. Source data

**Extended Data Fig. 6:**
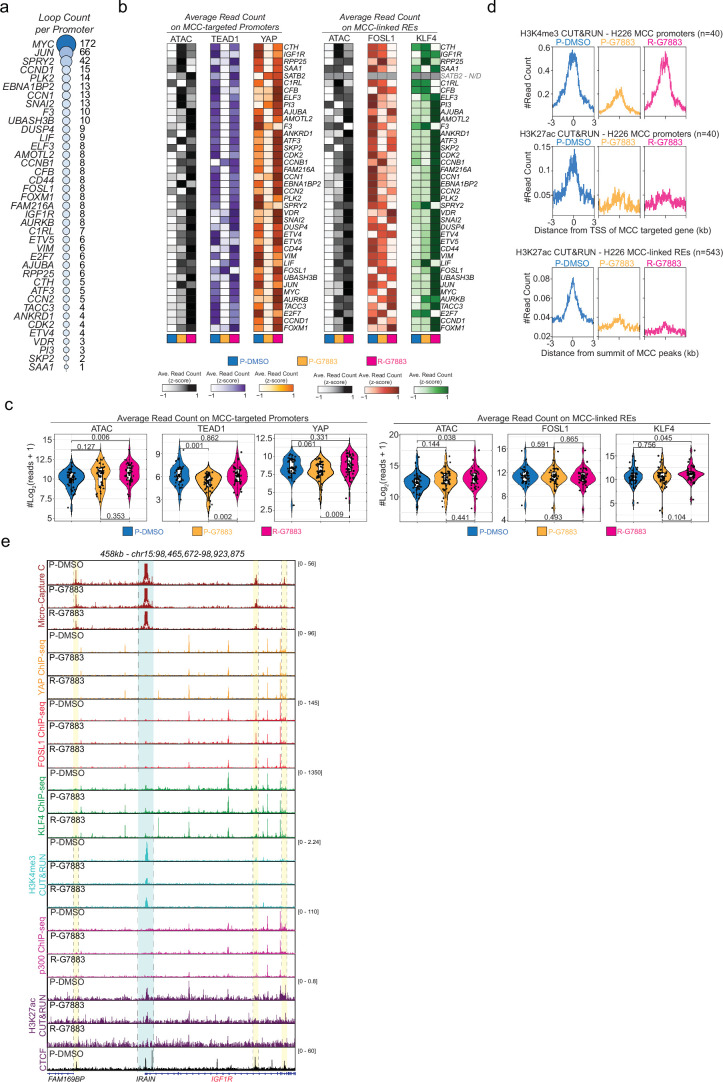
Chromatin features associated with MCC-identified promoter-regulatory element interactions. (**a**) Beads-on-a-string plot showing the number of MCC-called promoter loops for each gene, ordered from highest to lowest loop count. (**b**) Heatmaps of z-score-normalized average read counts for the indicated chromatin features at MCC-targeted promoters (left: ATAC-seq, TEAD1 ChIP-seq, YAP ChIP-seq) and at MCC-identified interacting RE (right: ATAC-seq, FOSL1 ChIP-seq, KLF4 ChIP-seq). (**c**) Violin plots quantifying normalized read count distributions for the indicated factors and chromatin accessibility at MCC-captured promoters (left) or interacting REs (right) (**d**) Aggregate CUT&RUN profiles showing normalized signal intensities of H3K4me3 and H3K27ac centered on the transcription start sites (TSSs) of MCC-target gene promoters (top). Bottom, aggregate H3K27ac CUT&RUN signal centered on the summits of MCC-linked distal regulatory elements. (**e**) Genome browser view of the *IGF1R* locus illustrating MCC, ChIP–seq, and CUT&RUN profiles across parental (DMSO), acute (G7883), and resistant (R-G7883) conditions. Blue shaded area highlights the gene promoter, and yellow shaded regions mark MCC-identified distal regulatory elements. Source data

To obtain an integrated view of the regulatory landscape at target promoters and their interacting REs, we combined transcriptomic and epigenomic datasets in the H226 model (Fig. 4 and Extended Data Fig. 6). MCC analysis revealed that target genes engaged variable numbers of promoter-associated chromatin interactions (Extended Data Fig. 6a). Across this set, gene expression and promoter activity showed highly concordant patterns, indicating that promoters remained transcriptionally active in resistant cells even when distal RE function was compromised (Fig. 4a,b). By contrast, genes repressed in resistance (for example, *CTH*, *IGF1R* and *SAA1*) exhibited reduced promoter activity.

Analysis of promoter-associated features revealed gene-specific and variable *FOSL1* binding, whereas *KLF4* occupancy increased at most target promoters in resistant cells (Fig. 4c,e). Promoter-associated histone 3 K4 trimethylation (H3K4me3) was reduced following acute GNE-7883 treatment but restored in resistance, whereas p300 and histone 3 K27 acetylation (H3K27ac) were suppressed by acute treatment and failed to fully recover, indicating incomplete restoration of histone acetyltransferase (HAT) activity at promoters (Fig. 4c and Extended Data Fig. 6d). TEAD1 and YAP occupancy displayed transient repression followed by restoration, and increased chromatin accessibility at most promoters in the resistant state, consistent with recovered promoter activity (Extended Data Fig. 6b). Resistance-repressed genes showed weaker or no acquisition of these features, except for partial recovery of TEAD1 and YAP binding (Fig. 4a–d).

At MCC-linked REs, interaction frequencies were broadly reduced in resistance and accompanied by near-complete restoration of TEAD1 binding, increased YAP and KLF4 occupancy, and enhanced chromatin accessibility; however, p300 and H3K27ac showed minimal recovery (Fig. 4d,f and Extended Data Fig. 6b,c,d), indicating that these regions did not fully regain enhancer activity. At representative loci (*FOSL1*, *SKP2* and *FAM216A*; Fig. 4g), reduced RE–P contacts coincided with promoter-proximal gains in KLF4 and FOSL1 binding and restored H3K4me3, despite diminished p300 and H3K27ac. By contrast, *IGF1R*, which was repressed by acute treatment and failed to recover in resistance, lacked promoter KLF4 or FOSL1 binding, showed no restoration of H3K4me3 or H3K27ac, and exhibited weakened distal RE interactions (Extended Data Fig. 6e).

Collectively, the results of this integrative analysis demonstrate that chronic TEAD inhibition reshapes regulatory architecture by limiting enhancer-associated acetylation and long-range chromatin looping, while enabling promoters to regain transcriptional activity marked by restored H3K4me3.

### Enhanced chromatin opening despite impaired HAT activity in resistance

**Extended Data Fig. 7:**
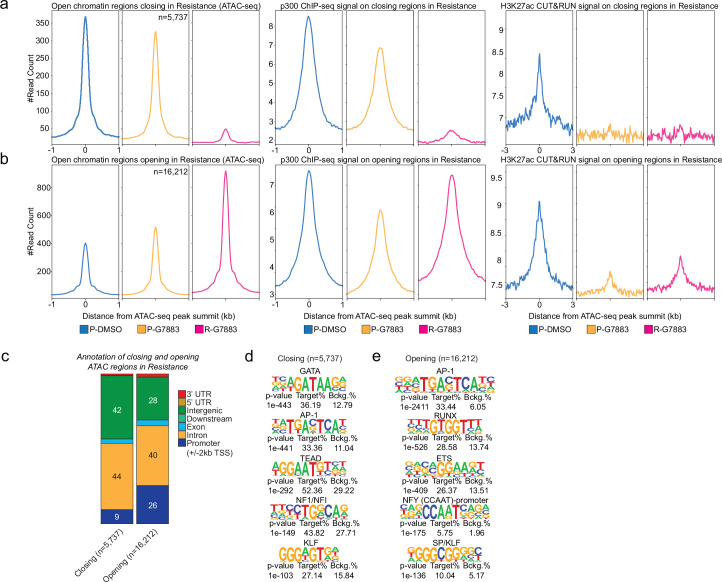
Genome-wide analysis of chromatin accessibility changes in resistant H226 cells. Aggregate ATAC-seq profiles showing normalized read coverage (#; Counts per million - CPM) at chromatin regions that are either lost (n = 5,737; FDR < 0.01, log_2_FC > 0.5) (**a**) or gained (n = 16,212; FDR < 0.01, log_2_FC > 0.5) (**b**) in the resistant state. Middle panels show corresponding p300 ChIP-seq signal, and right panels show H3K27ac CUT&RUN signal across parental (DMSO), acute GNE-7883-treated (P-G7883), and resistant (R-G7883) conditions. (**c**) Stacked bar plots showing the genomic annotation of closing and opening ATAC regions in resistant cells, categorized into promoters ( ± 2 kb TSS), intronic, exonic, downstream, distal intergenic, and UTR elements. (**d**–**e**) Transcription factor motif enrichment analysis at closing (**d**) and opening (**e**) ATAC regions in resistance, with representative motif logos shown along with associated statistical values (p-value, target percentage, and background percentage). Source data

To extend our promoter-centric findings from MCC-targeted genes, we next investigated global changes in chromatin accessibility to determine how resistant cells remodeled their RE landscape under sustained TEAD inhibition. ATAC-seq profiling of the H226 model revealed widespread chromatin alterations in resistant cells compared to parental cells (FDR < 0.01, log_2_FC > 0.5). We detected 16,212 opening and 5,737 closing ATAC regions in resistance, consistent with globally increased chromatin remodeling activity (Extended Data Fig. 7a,b). At closing regions, we observed marked loss of p300 and H3K27ac, consistent with enhancer decommissioning. By contrast, at opening regions, globally, p300 occupancy (which was reduced after acute GNE-7883 treatment) was restored in resistance, whereas H3K27ac showed only weak recovery (Extended Data Fig. 7a,b). Annotation of differential ATAC regions showed nearly three-fold more enrichment of promoter-proximal sites among opening regions, further highlighting promoters as major REs of recovery in resistance (Extended Data Fig. 7c). Motif analysis reinforced this distinction: closing regions were enriched for enhancer-associated motifs such as GATA, TEAD and NF1, whereas opening regions were enriched for AP-1, ETS, and promoter-associated NFY and SP/KLF motifs (Extended Data Fig. 7d,e).

Together, these findings suggest a genome-wide adaptation strategy in which TEADi-resistant cells increase chromatin remodeling activity to sustain transcription under conditions of reduced HAT function and weakened RE–P interactions, establishing a promoter-centric mode of transcriptional control.

### KLF4 regulates promoter-centric gene expression and growth in resistant cells

**Fig. 5:**
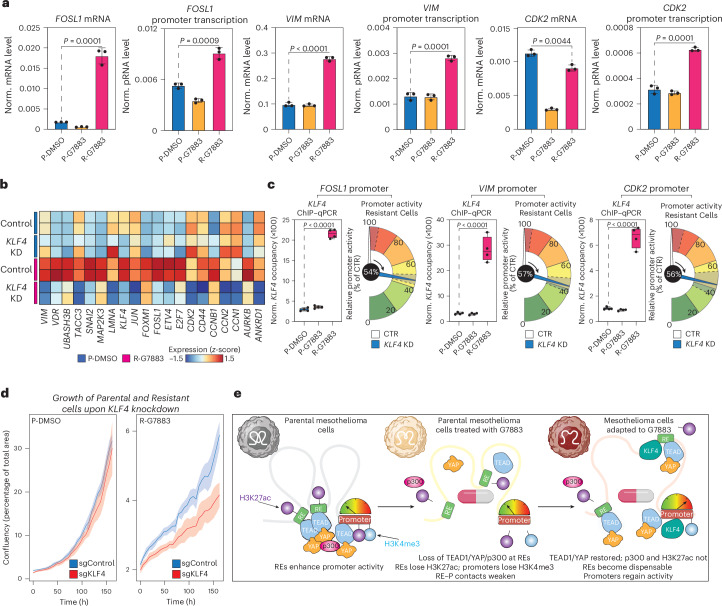
KLF4 is required for promoter-centric gene regulation in resistant cells. **a**, Bar plots showing *ACTB*-normalized mRNA and promoter transcript levels for *FOSL1*, *VIM* and *CDK2*. Data are presented as mean ± s.d.; significance was assessed by two-tailed unpaired *t*-tests (*P* < 0.05; *n* = 3 biological replicates). **b**, Heatmap of *ACTB*-normalized mRNA levels for the indicated genes (*n* = 20) following *KLF4* knockdown in parental and resistant cells (qPCR; two-tailed unpaired *t*-test, *P* < 0.05; *n* = 2 biological replicates). **c**, Box plots showing KLF4 ChIP–qPCR enrichment at the indicated gene promoters. Boxes represent the IQR, the center line indicates the median, and whiskers extend to the minimum and maximum values; all data points are shown. Significance was assessed by two-tailed unpaired *t*-tests (*P* < 0.05; *n* = 4; two biological replicates with two technical replicates each). Gauge plots show *ACTB*-normalized promoter transcript levels in resistant cells under control and *KLF4* knockdown conditions, relative to control (nontargeting guide). Pins indicate mean values, and shaded regions indicate ±s.d. across replicates. Percentage values indicate reduction relative to control (two-tailed unpaired *t*-test, *P* < 0.05; *n* = 4; two biological replicates with two technical replicates each). **d**, Real-time live-cell measurements of parental (left) and GNE-7883-resistant (right) cells following *KLF4* (red) or control (blue) knockdown over 7 days. Lines represent mean confluency and shaded areas indicate ±s.d. (*n* = 6 biological replicates). **e**, Model of promoter reinforcement in TEADi resistance. In parental cells, distal REs contact promoters and support gene expression. Acute TEAD inhibition disrupts YAP binding and enhancer acetylation and weakens RE–P interactions. In resistant cells, RE–P contacts are further reduced, distal REs lose functional contribution and promoters regain activity, as marked by restoration of H3K4me3 and recruitment of TFs such as KLF4, enabling promoter-centered gene expression despite incomplete enhancer reactivation. CTR, control; KD, knockdown; norm., normalized; pRNA, promoter-associated RNA. Panel **e** created in BioRender; Daniel, B. https://biorender.com/gicmylv (2026). Source data

**Extended Data Fig. 8:**
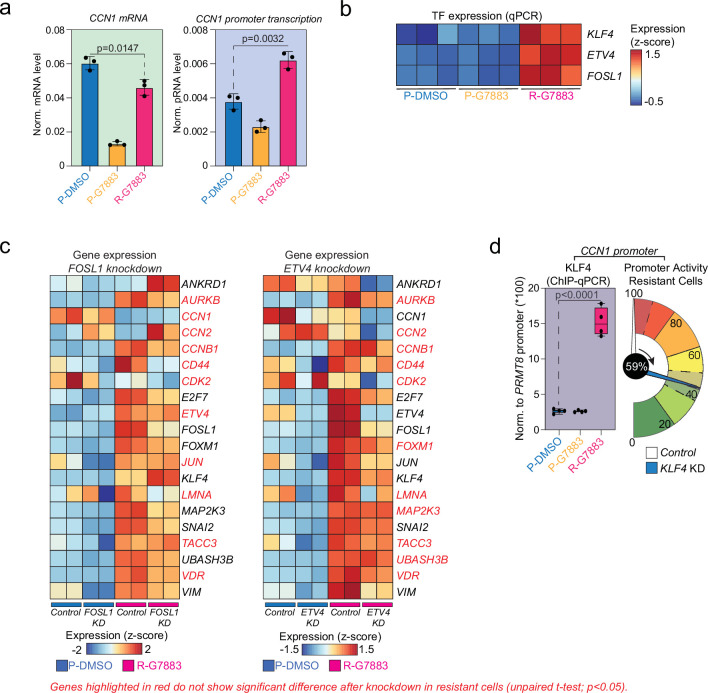
Functional analysis of promoter-associated transcription factors in parental and resistant H226 cells. (**a**) Bar plots showing normalized *CCN1* mRNA levels (left) and *CCN1* promoter-derived transcript levels (right) across parental (DMSO), acute GNE-7883-treated (P-G7883), and resistant (R-G7883) H226 cells. Statistical significance was determined using two-tailed unpaired *t*-tests (*p* < 0.05). *n* = 3 biological replicates. (**b**) Heatmap showing *ACTB*-normalized expression (z-score) of selected transcriptional regulators (*KLF4*, *ETV4*, *FOSL1*) across parental, acutely treated, and resistant H226 cells measured by RT-qPCR. (**c**) Heatmaps of gene expression (z-score) after CRISPR knockdown of *FOSL1* or *ETV4* in parental and resistant H226 cells. Two biological replicates are shown for each condition. Genes labeled in red do not show significant changes upon TF knockdown in resistant cells (two-tailed unpaired *t*-tests, *p* < 0.05). (**d**) Boxplots showing ChIP-qPCR enrichment of KLF4 at the *CCN1* promoter in H226 cells across indicated conditions. Box represents the interquartile range (IQR), internal line indicates the mean, and whiskers extend to minimum and maximum values. *n* = 4 (two biological replicates and two technical replicates). Statistical significance was assessed using two-tailed unpaired *t*-tests (*p* < 0.05). Right, gauge plot showing *CCN1* promoter transcript levels in resistant cells following *KLF4* knockdown relative to control (non-targeting guides). Pin indicates the average, and shaded region denotes standard deviation across replicates. *n* = 4 (two biological replicates and two technical replicates). Statistical significance determined by two-tailed unpaired *t*-tests (*p* < 0.05). Source data

To further investigate promoter-dependent mechanisms that restored gene expression in resistant H226 cells, we quantified promoter activity by measuring promoter-derived transcripts using real-time quantitative PCR (RT–qPCR). Primers were designed to capture divergent transcription upstream of active TSSs^47^. We assessed promoter transcripts and mRNA levels for *FOSL1*, *VIM*, *CDK2* and *CCN1* in parental and resistant cells (Fig. 5a and Extended Data Fig. 8a). For all four genes, promoter activity was elevated in the resistant state, accompanied by enhanced or restored mRNA expression.

We next tested the functional roles of TFs induced in resistance by CRISPR-mediated knockdown of *FOSL1*, *KLF4* and *ETV4* in parental and resistant cells (RT–qPCR; Extended Data Fig. 8b). Whereas perturbation of each TF affected expression of a subset of TEAD target genes examined by MCC, KLF4 depletion uniquely reduced expression of all 20 measured genes in a resistance-specific manner (unpaired two-tailed *t*-test, *P* < 0.05; Fig. 5b and Extended Data Fig. 8c). These experiments also revealed a hierarchical relationship in which *KLF4* maintains resistance-specific expression of *FOSL1* and *ETV4*, whereas *FOSL1* and *ETV4* have minimal impact on *KLF4* expression. Consistent with these results, ChIP–qPCR confirmed resistance-induced enrichment of KLF4 at the promoters of *FOSL1*, *VIM*, *CDK2* and *CCN1* (Fig. 5c and Extended Data Fig. 8d). In addition, KLF4 knockdown reduced promoter transcript levels at all four loci by >50% in resistant cells, validating its role in sustaining promoter activity (Fig. 5c and Extended Data Fig. 8d). Finally, loss of KLF4 significantly impaired proliferation of resistant cells, whereas parental cell proliferation was not affected (Fig. 5d).

Taken together, these data support a model in which TEADi-resistant cells rely on promoter-centric gene regulation, driven in part by KLF4, to sustain gene expression despite the loss of RE–P interactions (Fig. 5e).

## Discussion

Cancer cells employ diverse mechanisms to adapt to selective pressures, including those imposed by targeted therapies^48,49^. Here we describe a compensatory gene regulatory response, promoter reinforcement, that is associated with acquired resistance to TEAD inhibition in mesothelioma cells. Using MCC, global epigenetic profiling and genome editing, we show how resistant cells recover from the initial transcriptomic stress induced by TEAD inhibition.

By assessing promoter interaction profiles at 90 loci across two mesothelioma cell lines (H226: 40 promoters; MSTO: 50 promoters), we find that although TEADi-resistant cells regain YAP–TEAD1 binding genome-wide, this occurs within a dysregulated chromatin context in which many of our target promoters become physically uncoupled from their distal REs. Although alterations in 3D genome organization in cancer are often interpreted through enhancer hijacking or reorganization of topologically associating domains driven by structural variation and epigenetic reprogramming^50^, our data demonstrate that the expression of the studied TEADi-sensitive genes can undergo restoration—and, in many cases, gain—of expression without re-establishing RE–P connectivity in the resistant state. Instead, our results identify promoters as key regulatory units under these conditions in our gene set, supported by four lines of evidence: (1) distal REs lose enhancer-associated features (including H3K27ac and p300), become uncoupled from target promoters, and are functionally dispensable in resistant cells; (2) promoters regain transcriptional activity and H3K4me3; (3) resistance-associated TFs, including FOSL1 and KLF4, exhibit promoter-biased localization; and (4) resistant cell viability is selectively impaired upon loss of KLF4 and FOSL1^37^. Collectively, these findings support promoter reinforcement as a regulatory strategy that mesothelioma cells can engage under selective pressure exerted by small-molecule inhibition of TEAD TFs.

Our observation that gene expression becomes uncoupled from distal RE activity at TEADi-sensitive loci is consistent with findings of Martin et al., who showed that acute inhibition of SWI/SNF chromatin remodeling complexes compromises enhancer activity while promoters recover accessibility through the EP400 complex^51^. Whereas their study captured an immediate compensatory response to chromatin remodeler loss, our work extends this concept by establishing promoter reinforcement as a stable regulatory feature in a drug-resistant state^51^. In addition, global loss of enhancer–promoter communication has been reported in the context of carcinogenesis^52^, suggesting that promoter-centric regulatory behavior may represent a broader mode of transcriptional adaptation in cancer.

In summary, our findings reveal a shift in gene regulatory logic in TEADi-resistant mesothelioma cells, in which promoter-centered regulation can support transcriptional resilience under therapeutic pressure. Although this study focuses on a defined set of TEAD-responsive loci, our results raise the possibility that promoter reinforcement may contribute more broadly to adaptive gene regulatory responses in cancer. Determining the generality and therapeutic implications of this mechanism across additional drug-resistant cancer models will be an important direction for future work.

## Methods

### Cell lines and establishment of resistant cells

Two established preclinical models of malignant mesothelioma were used in this study: the NCI-H226 (H226) cell line, which is NF2-null; and the MSTO-211H (MSTO) cell line, which carries a PSEN1–LATS1 fusion leading to loss of LATS1 kinase activity and impaired phosphorylation of YAP. MSTO cells also harbor a 42-bp deletion in exon 5 of LATS2 that causes its inactivation^33,54,55^. Cell lines were obtained from the American Type Culture Collection and maintained in RPMI-1640 medium supplemented with 10% fetal bovine serum (Sigma-Aldrich), 2 mM L-glutamine (Gibco Life Sciences) and penicillin–streptomycin, in a humidified incubator at 37 °C with 5% CO_2_. GNE-7883-resistant H226 and MSTO cells were generated by long-term, stepwise dose adaptation to pan-TEADi GNE-7883. H226 cells were gradually exposed from 0.25 µM to 2.5 µM over ~55 days, and MSTO cells were exposed from 0.25 µM to 3.0 µM, with incremental increases every 1–2 weeks while maintaining at least 25% cell confluence. During escalation, transient cytostasis was followed by the emergence of proliferating persister populations under sustained drug pressure. Resistant pools were expanded from these outgrowths and maintained in medium containing 5 µM GNE-7883. Characterization of these resistant models followed previously described procedures^37^. For acute treatment conditions, H226 and MSTO cells were treated with 2.5 µM GNE-7883 for 48 h before collection.

### RNA isolation

RNA was isolated from 5 × 10^5^ cells by a standard TRIzol-based RNA precipitation method as follows. Cells were resuspended in 1 ml TRIzol (Ambion). Chloroform (200 μl) was added to this lysate, followed by extensive vortexing to obtain a homogenous mixture; then, the mixture was incubated for 3 min at room temperature before being centrifuged at 14,000*g* (relative centrifugal force) at 4 °C for 15 min. Aqueous layer was collected from the top and transferred into a new tube (~550μl), and the RNA was precipitated with equal volume of 2-propanol for 20 min at room temperature. RNA precipitates were centrifuged at 16,000*g* for 15 min at 4 °C, and the supernatant was carefully discarded without disturbing the RNA pellet. The RNA pellet was washed with 1 ml 75% EtOH and then dissolved in 30 μl nuclease-free water. RNA concentration was determined with a NanoDrop spectrophotometer.

### Real-time RT–qPCR

RNA (1 μg) was reverse transcribed with a High-Capacity cDNA Reverse Transcription Kit (Applied Biosystems) according to the manufacturer’s instructions. Transcript quantification was performed by qPCR using SYBR green master mix (Applied Biosystems). Primers for mRNA transcript detection were designed for exon junctions, whereas those for promoter transcript measurements were designed to overlap with gene promoter regions not more than 100 base pairs from the TSS of the gene. Transcript levels (mRNA) were normalized to *ACTB* expression. Primer sequences are available in Supplementary Table 7.

### Visualization of promoter activity on gauge plots

To visualize normalized gene expression levels as determined by real-time qPCR for promoters across different conditions, we generated gauge plots. Gene expression levels were measured in biological replicates (*n* = 4) for each condition, and average expression levels and standard deviations were calculated. Expression levels were normalized to the levels of the control perturbation that represented the maximum value of 100% to facilitate comparison across conditions. Gauge plots were created using the ggplot2 and tidyr libraries in R. Each plot featured a needle indicating the relative expression level, a central value displayed as a percentage of the control guide treated condition, and a shaded area with dashed lines representing the standard deviation. A color gradient was used to represent different ranges of expression levels: forest green (0–20%), yellow-green (20–40%), yellow (40–60%), orange (60–80%) and red (80–100%).

### RNA-seq

Approximately 500 ng of RNA was used for library synthesis using a TrueSeq RNA Sample Preparation kit v2 (Illumina). The sizes of libraries were confirmed using a 2200 TapeStation, and the concentration was determined by a qPCR-based method using a library quantification kit (KAPA). Libraries were sequenced on an Illumina HiSeq 2500 to generate an average of 56 million single-end 50-base reads per sample.

### RNA-seq analysis

RNA-seq data were analyzed using HTSeqGenie (R package v.4.30.0)^56^ in the Bioconductor framework as follows: reads characterized by low nucleotide qualities (defined as having 70% of bases with quality scores below 23) or those matching rRNA and adapter sequences were excluded. The remaining reads were aligned to the human reference genome (human: GRCh38.p10) using GSNAP (2013-10-10-v2) with a maximum allowance of two mismatches per 75-base sequence (using the following parameters: ‘-M 2 -n 10 -B 2 -i 1 -N 1 -w 200000 -E 1–pairmax-rna=200000–clip-overlap’)^57,58^. The aligned reads were quantified using the featureCounts function from the Rsubread package, with GTF annotation file ‘Homo_sapiens.GRCh38.104.gtf‘^59,60^. The count matrix was extracted for downstream analysis. The count matrix was imported into the edgeR package for differential expression analysis^61^. Genes with low expression were filtered out using the filterByExpr function, when gene expression was consistently low across all conditions. Data were normalized using the calcNormFactors function. A design matrix was constructed to model the experimental conditions, and dispersion estimates were obtained using the estimateDisp function. Differential expression was assessed using the quasi-likelihood F-test implemented in the glmQLFit and glmQLFTest functions. Gene annotations were retrieved from the Ensembl database using the biomaRt package. Ensembl gene IDs were mapped to external gene names, and genes without external names were retained with their Ensembl IDs. Differentially expressed genes were identified based on cutoffs of log_2_FC > 1 and FDR < 0.01 in a three-way comparison analysis strategy to retain all differential genes across conditions. The expression data for these genes were log-transformed and standardized (z-score normalization). The *z*-scores were reported with three significant digits. Hierarchical clustering was performed using the hclust function, and the dendrogram was cut into seven clusters using the cutree function. Heatmaps were generated using the pheatmap package. Average expression levels for each cluster across the three conditions were calculated and visualized using line plots. The ggplot2 package was used to create line plots, with shaded areas representing the standard deviation of expression levels within each cluster. IPA (Qiagen) was used to predict upstream regulators and biological pathways for each gene cluster. For IPA, the gene lists of each RNA-seq cluster were loaded, and core analysis was performed with default settings. The list of upstream regulators was filtered for TFs and is reported in Supplementary Table 2.

### Promoter activity analysis

Promoter activity was quantified following the framework described by Demircioğlu et al.^45^, using the proActiv R/Bioconductor package (v.1.20). Promoter coordinates were derived from the Ensembl/GENCODE v.38 (GRCh38.104) GTF annotation. Promoter activity was calculated for each annotated TSS from junction-spanning reads. For each model, only RNA-seq data from nine matched samples (three P-DMSO, three 7883-treated and three 7883-resistant replicates) were analyzed independently. Absolute promoter activity was defined to represent total expression from a promoter. The proActiv output matrices were postprocessed in R (v.4.3). Gene identifiers were mapped to gene symbols using the corresponding Ensembl GTF. Promoters were filtered to retain entries with sufficient read support and nonzero variance across replicates. Mean promoter activity per condition was used as a covariate for downstream weighting. Differential promoter activity between resistant and parental conditions was evaluated using the limma moderated *t*-statistic. log_2_FC (resistant–parental) and Benjamini–Hochberg adjusted *P* values were obtained for absolute promoter activities. Statistical significance was defined based on a local false sign rate ≤ 0.10 as estimated with the ashr package (method = ‘fdr’) to provide directionally robust discovery sets. Heatmaps were generated in R (v.4.3), and hierarchical clustering was performed on all differential promoters (P-DMSO versus R-G7883 cells from both cell line models) using Pearson correlation distance and complete linkage.

### Upstream regulator analysis and integration with expression data

Upstream regulator analysis was performed using IPA as part of a standard core analysis, applying default parameters. This analysis predicts upstream transcriptional regulators that could explain observed differential gene expression patterns based on curated causal relationships within the Ingenuity Knowledge Base. For each regulator, IPA computes an activation *z*-score, indicating the direction and magnitude of predicted activity, and a corresponding overlap *P* value, reflecting the statistical significance of the enrichment between known regulator targets and the input gene list. Only regulators annotated as transcriptional regulators were retained for further interpretation. Predicted upstream transcriptional regulators identified by IPA were compared with TFs expressed in our model system. Expression data (Supplementary Table 4) were filtered to retain TFs expressed in C1, C4 and C5, which represented the biologically relevant groups for this study. We took the intersection of these expressed TFs with the IPA-predicted regulators (Supplementary Table 2) to identify common TFs predicted and detected at the expression level. Overlapping TFs were visualized using heatmaps showing normalized expression values across experimental conditions (H226 DMSO → H226 G7883 → CL3 G7883). Corresponding IPA −log_10_ *P* values for each TF were displayed using bead plots, in which bead size reflected the strength of statistical significance. Both plots were aligned to the same gene order to enable direct comparison between TF expression and predicted regulatory significance.

### ATAC-seq

For each sample, ~100,000 viable cells were processed with an Active Motif ATAC-seq kit according to the manufacturer’s instructions. Nuclei were isolated, tagmentation was performed with Tn5 transposase for 30 min at 37 °C, and libraries were PCR amplified (12 cycles) using indexing primers. Libraries were size-selected, quality-checked with TapeStation, quantified with Qubit and sequenced as paired-end (2 × 50 bp).

### ATAC-seq computational analysis

Raw data were processed with the ENCODE ATAC-seq pipeline (v.2.2.2) under default settings^62^. Adapters were trimmed with cutadapt (v.2.5)^63^, reads were aligned to hg38 with Bowtie2 (v.2.3.4.3)^64^, and alignments were filtered for mapping quality and duplicates using samtools (v.1.9)^65^ and Picard (v.2.20.7) (Broad Institute). Peaks were called with MACS2 (v.2.2.4) using Tn5 offset correction and smoothing^66^, and hg38 ENCODE blacklist regions were removed^67^. Reproducibility was assessed with IDR (v.2.0.4.2)^68^, and standard quality control was performed, including mapping statistics, library complexity, replicate concordance and TSS enrichment. Differential accessibility was then assessed in R with DESeq2 modeling^69^ in DiffBind^70^ at FDR < 0.01 and log_2_FC > 0.5. For visualization and summary profiling, bigWig coverage tracks were generated and normalized to counts per million (CPM), and aggregate signal profiles for differential opening and closing peak sets were produced with deepTools (computeMatrix, plotHeatmap, plotProfile)^71^. Peaks were annotated with ChIPseeker using TxDb.Hsapiens.UCSC.hg38.knownGene and org.Hs.eg.db^72^, and summaries were visualized in ggplot2 as stacked bar plots. Motif enrichment on differential peak sets was performed with HOMER with size-given, masked searches against a matched background^73^.

### Chromatin immunoprecipitation followed by sequencing

ChIP–seq was performed as previously described^74^ with the following modifications. NCI-H226 (H226) and MSTO-211(H) (MSTO) mesothelioma cells (10 × 10^6^) were double crosslinked with 50 mM disuccinimidyl glutarate (C1104, ProteoChem) for 30 min, followed by 10 min with 1% formaldehyde. Formaldehyde was quenched by addition of glycine. Nuclei were isolated with ChIP lysis buffer (1% Triton X-100, 0.1% SDS, 150 mM NaCl, 1 mM EDTA, and 20 mM Tris pH 8.0) then sheared with Covaris sonicator using the following setup: fill level, 10; duty cycle, 15; PIP, 350; cycles per burst, 200; time, 8 min). Sheared chromatin was immunoprecipitated overnight with antibodies against FOSL1 (PA5-66880, Invitrogen; 5 μg), YAP (14074S, Cell Signaling; 2 μg) and KLF4 (AF3158, R&D Systems; 5 μg). Antibody chromatin complexes were pulled down with Protein A magnetic beads and washed twice in IP wash buffer I (1% Triton, 0.1% SDS, 150 mM NaCl, 1 mM EDTA, 20 mM Tris pH 8.0, and 0.1% NaDOC), twice in IP wash buffer II (1% Triton, 0.1% SDS, 500 mM NaCl, 1 mM EDTA, 20 mM Tris pH 8.0, and 0.1% NaDOC), twice in IP wash buffer III (0.25 M LiCl, 0.5% NP-40, 1 mM EDTA, 20 mM Tris pH 8.0, 0.5% NaDOC) and once in TE buffer (10 mM EDTA and 200 mM Tris pH 8.0). DNA was eluted from the beads by vigorous shaking for 20 min in elution buffer (100 mM NaHCO_3_, 1% SDS). DNA was decrosslinked overnight at 65 °C and purified with a MinElute PCR purification kit (Qiagen). ChIP–qPCR experiments were carried out as described above with antibody against KLF4 (AF3158, R&D Systems). DNA was quantified by Qubit, and equal amounts (2 ng) of DNA were used for sequencing library construction with an Ovation Ultralow Library System V2 (Tecan) using 15 PCR cycles according to the manufacturer’s recommendations. Libraries were sequenced with an Illumina NextSeq 2000, using a paired-end 50-bp read configuration. CTCF cleavage under targets and tagmentation (CUT&Tag) data for MSTO cells were obtained from Barbosa et al.^53^ (SRA BioProject ID: PRJNA949402).

### ChIP–seq analysis

ChIP–seq results were analyzed using the ENCODE ChIP–seq pipeline (v2.2.1)^62^. ChIP–seq reads were aligned to the human reference genome (hg38) using Bowtie2 (v2.3.4.3)^64^. Aligned reads were then filtered for quality and duplicates using samtools (v1.9)^75^ and Picard (v2.20.7, Broad Institute). The SPP peak caller was used to call ChIP–seq peaks for FOSL1 and YAP, and input was used to assess the background of the experiments^76^. Peak sets were filtered using a list of genomic regions that contain anomalous, unstructured, or experiment independent high signals. ChIP–seq BAM and BED files were then used to call differential peaks by using DiffBind (v3.12.0)^70,77^. Briefly, the differential binding analysis object was created by loading the bam and bed files with the dba() function. Reads were counted with the dba.count() function, followed by depth normalization using the dba.normalize() function, and differential peaks were called across the conditions with the dba.analyze() function using DESeq2 and the following statistical parameters (FDR < 0.01).

### Identification and characterization of decreased and increased YAP and FOSL1 peaks

Differential peaks were retrieved for two contrasts (P-DMSO versus P-GNE-7883; and P-DMSO versus R-GNE-7883) with a significance threshold of FDR < 0.01. Peaks were categorized into gained and lost occupancy groups based on FC values, and new differential binding analysis objects were created for these peak sets. To visualize the overlaps between the two contrasts, we generated Venn diagrams using the dba.plotVenn() function; these illustrated the unique and common peaks between the two conditions, highlighting the overlap of gained or lost peaks. Specifically, the Venn diagrams allowed us to identify three categories of peaks: those unique to the first contrast, those unique to the second contrast and those common to both contrasts. The locations of differential peaks were then written into BED files, and unique and common regions were identified for both gained and lost peaks. For the overlap analysis, we used GenomicRanges to determine which peaks were specific to each condition and which were shared^78^. This involved comparison of the genomic coordinates of peaks from both contrasts to identify regions of overlap and uniqueness, with an overlap defined as any shared region with at least 1 base pair in common. We performed motif analysis with the BED files that contained the different peak sets using HOMER with default settings to identify enriched motifs within the unique and common peak sets^73^. Subsequently, peaks were annotated using the ChIPseeker package (v.1.38.0) with the TxDb.Hsapiens.UCSC.hg38.knownGene and org.Hs.eg.db databases^79^. For data visualization, annotated peak data were plotted as stacked bar plots using ggplot2; these illustrated the genomic localization of peaks by showing the distribution of peak annotations across different genomic features. Promoters were defined as ±2 kb upstream and downstream of the TSSs of the annotated genes per default settings. We used the BED files generated from the differential peak analysis to create coverage heatmaps for FOSL1 binding sites (Fig. 2) and histograms for YAP binding sites (Fig. 1) with the computeMatrix and plotProfile functions from the deepTools suite^71^. The BED files representing unique and common regions for gained and lost peaks were used as input to calculate the coverage from bigWig files corresponding to different experimental conditions (P-DMSO, P-GNE-7883, R-GNE-7883). For each BED file, we computed the coverage matrix centered on the summit of the peaks (reference point analysis) with a window of 3,000 bp upstream and downstream. Heatmaps (FOSL1) and histograms (YAP) were then generated to visualize the coverage profiles and provide insight into the binding patterns of FOSL1 and YAP across different conditions.

### Micro Capture-C

MCC was performed as previously described^21,22^ with the following differences. Cells were scraped and crosslinked in the presence of 1% formaldehyde (in phosphate-buffered saline; PBS) for 10 min in suspension, followed by addition of 1.5 ml 1 M glycine and further incubation for 5 min at room temperature. Cells were washed in ice-cold PBS and counted, and 3–4 × 10^6^ cell aliquots were prepared for isolation of nuclei with the following MCC lysis buffer: 10 mM Tris-HCl pH 7.5, 10 mM NaCl, 3 mM MgCl_2_, 0.1% Tween-20, 0.1% Nonidet P40 Substitute, 0.01% digitonin and 1% bovine serum albumin in nuclease-free H_2_O. Each aliquot of 3 × 10^6^ cells was resuspended in 500 μl MCC lysis buffer and incubated on ice for 5 min. Cells were spun down, washed in ice-cold PBS, and washed again in nuclease-free H_2_O before being subjected to MNase digestion with 90–130 U of MNase per 3 million cells for 1 h at 37 °C with shaking in a thermomixer (Eppendorf) at 550 rcf. MNase digestion was stopped by addition of EGTA. At this point, nuclei were split into two tubes, and end repair and proximity ligation were performed overnight. The next day, nuclei were spun and subjected to proteinase K digestion at 65 °C overnight. DNA was column purified (Minelute, Qiagen) and eluted in 130 μl H_2_O for sonication with a Covaris E220 model using the following settings: fill level, 10; duty cycle, 15; PIP, 300; cycles per burst, 200; sonication time, 5 min. DNA was cleaned up with Minelute, quantified and subjected to library preparation with Ovation Ultralow V2 Library Systems (Tecan) using 100 ng DNA and eight PCR cycles. Amplified libraries were subjected to hybridization with biotinylated oligonucleotide pools that were designed by the HyperDesign Team (Roche) and synthesized by Roche. All hybridization reagents were purchased from Roche as part of the KAPA Hyper Prep kit, and hybridization, pull-down, and washes were performed per the manufacturer’s instructions. Bead-bound libraries were amplified with PCR reagents from the Ovation Ultralow Library V2 Kit (Tecan). Libraries were quantified and sequenced on a NovaSeq 6000 with a 2 × 150 bp configuration. MCC analysis was performed on two biological replicates per condition, and each biological sample was defined as a pooled total of 3 different MNase conditions (90 U, 110 U and 130 U, 3 × 10^6^ cells per MNase reaction), each split into two ligation reactions for subsequent downstream steps as described above.

### MCC analysis

Paired-end sequencing data were processed using a custom pipeline implemented in Bash and Python (https://github.com/jhoover204/MCCTools/tree/v1.0.0). Briefly, adapter sequences were trimmed from FASTQ files using TrimGalore (v.0.6.10, Babraham Institute). Reads were aligned to reference genome hg38 using BWA mem^65^ (v.0.7.18), and the resulting files were parsed to identify junction contacts using pairtools parse2 (v.1.1.0)^80^. Next, reads were sorted and deduplicated using pairtools sort and pairtools dedup and converted to BED files using a custom Python script. Promoter-linked junction pairs were identified by intersecting the BED files with a reference file containing genomic regions of interest, and the results were filtered and split into different viewpoints. Filtered BED files were converted to BAM files with sorting and indexing using bedToBam from bedtools (v.2.26)^81^, and bigwig coverage files were generated and normalized using deeptools bamCoverage (v.3.5.1)^71^ with −normalizeUsing CPM.

### Peak calling in MCC datasets

MCC coverage was quantified from duplicate-free alignments after merging all replicates from all conditions and converted to CPM-normalized bigWig tracks at 25 bp resolution using pyBigWig (v.0.3.22) and NumPy (v.1.24.4). For each capture viewpoint, the signal within ±1 Mb of the bait midpoint was extracted, with the central ±2.5 kb excluded to avoid local ligation artifacts. The bigWig-derived signal arrays were then processed using custom Python code built on NumPy, including Gaussian smoothing (*σ* = 1 bin), computation of a local background via a 10-kb running median, and calculation of median absolute deviation (MAD) values for thresholding. Candidate MCC peaks were defined as regions with signal ≥3.5 × MAD above the local baseline for at least 3 consecutive bins (≥150 bp). Adjacent candidates separated by ≤150 bp were merged, and broad plateaus longer than 1 kb were subdivided at local valleys in which the signal dropped by ≥40% relative to the local maximum. To increase biological specificity, MCC peaks were further filtered to retain only those overlapping accessible chromatin regions from matched ATAC-seq profiles. ATAC accessibility was identified using the same NumPy-based local background and MAD-thresholding framework (threshold ≥5 × MAD; minimum width: 200 bp), applied to pyBigWig-extracted coverage tracks. All MCC and ATAC peak-calling procedures described above were implemented in Python scripts that operated directly on bigWig files through pyBigWig and performed all smoothing, baseline estimation, MAD computation, thresholding, merging and segmentation using NumPy. This produced a fully reproducible, parameterized peak-calling workflow conceptually analogous to the local *λ* background estimation in MACS2 but optimized for sparse, capture-based contact profiles as detailed above.

### MCC peak annotation

MCC peak annotation was performed independently for the MSTO and H226 mesothelioma cell lines. Capture regions were defined as ±1-Mb windows centered on each assay viewpoint, derived from curated MCC-relevant gene lists, with an interval of ±2.5 kb removed from around the midpoint of each viewpoint to prevent self-overlap. All analyses used the hg38 reference genome (chromosome sizes from hg38.chrom.sizes) and gene models from Ensembl GRCh38.104. For each cell line, the nonredundant MCC peak sets were restricted to those within the viewpoint-defined windows, forming the MCC peak universes used for downstream classification. Additional regulatory reference sets included CTCF peaks that originated from our own CTCF ChIP–seq experiment in parental H226 cells, and a public dataset of CTCF cleavage under targets and release using nuclease (CUT&RUN) from MSTO cells^53^ and YAP peaks from both H226 and MSTO cells. Promoters were defined globally within the viewpoint windows as TSS ± 2 kb. TSSs were extracted from the Ensembl GTF (‘transcript’ entries), converted to strand-aware 1-bp positions, filtered to retain only those falling inside the capture windows (after application of the ±2.5 kb exclusion), expanded by ±2,000 bp and merged into a nonredundant promoter BED file. Each MCC peak was assigned to one of eight mutually exclusive regulatory categories based on its overlap with the CTCF, YAP and promoter sets, and different combinations of these sets using bedtools intersect (v.2.30.0): CTCF_only, YAP_only, PROM_only, CTCF_YAP, CTCF_PROM, YAP_PROM, CTCF_YAP_PROM, or other (denoting MCC peaks that overlapped with ATAC-seq peaks but not other features). Genomic context annotations were obtained in R using ChIPseeker, with TxDb.Hsapiens.UCSC.hg38.knownGene and org.Hs.eg.db, applying a promoter definition of ±2 kb (tssRegion = c(−2000, 2000)). Annotations were exported to CSV, and stacked fractional bar plots (promoter, untranslated region, exon, intron, downstream, distal intergenic) were generated for both cell lines.

### Quantification of sequencing reads on MCC target promoters and linked REs

For promoter-associated occupancy analysis, ChIP–seq and CUT&RUN read densities for FOSL1, KLF4, YAP, TEAD1, H3K4me3, p300 and H3K27ac were quantified across MCC promoter target regions. Aligned BAM files were used as input (two biological replicates per condition: DMSO, P-7883 and R-7883). Read counts were computed using bedtools multicov (v.2.30.0^81^) over fixed promoter intervals defined in BED format. Regions were taken exactly as the promoter coordinates provided. Replicate counts were summed per region to yield a single value per gene promoter and condition. Normalized counts (CPM) were log_2_-transformed after addition of a pseudo-count (+1). For violin plots, values were visualized as log_2_(reads + 1) using ggplot2 (v.3.5.0) and ggsignif (v.0.6.4). Distributions across conditions were compared using nonparametric Wilcoxon rank-sum tests (*P* < 0.05), with multiple comparisons displayed directly on the plots. Per-gene normalized read intensities were also converted to *z*-scores (row-wise scaling) for heatmap visualization using pheatmap (v.1.0.12). To assess long-range interaction frequency from MCC and read coverage from ChIP–seq and CUT&RUN, we used merged RE–P contact peak sets identified as input. Each peak was annotated to its target promoter within the combined BED file. MCC interaction intensities were computed using bedtools multicov across the same three aggregated MCC libraries. Counts from all peaks associated with a given gene were summed to yield total MCC interaction read counts per gene and condition. Aggregate per-gene counts were log_2_-transformed and row-scaled (*z*-score normalization) before visualization. Heatmaps were drawn in pheatmap, with hierarchical clustering performed on the expression profiles of MCC-targeted genes from RNA-seq. For heatmaps, each gene’s signal was internally standardized by computing a row-wise *z*-score across the three conditions (DMSO, P-7883, R-7883), emphasizing relative enrichment patterns. For violin plots, we displayed normalized log_2_(reads + 1) to stabilize variance and visualize the distribution of promoter or interaction intensities across genes. Thus, absolute log_2_ counts reflect aggregate binding and contact strength distributions, whereas *z*-scores indicate condition-specific shifts for each gene independently.

### CUT&RUN

CUT&RUN profiling of H226 and H226-7883R cells was performed using a CUTANA ChIC/CUT&RUN Kit (EpiCypher, 14-1048) following the manufacturer’s instructions and as described by Paul et al.^37^. Briefly, cells were prepared as single-cell suspensions, immobilized on activated ConA beads, and incubated with SNAP-Certified histone modification antibodies for H3K27ac (EpiCypher 13-0059, clone 2114-3E4) and H3K4me3 (EpiCypher 13-0060) at ~0.5 µg per reaction. After primary antibody binding, pAG-MNase was added, and chromatin digestion was activated with CaCl_2_. Released DNA fragments were purified and quantified. Sequencing libraries were constructed using a CUTANA CUT&RUN Library Prep Kit (EpiCypher, 14-1001/14-1002), with end repair, adapter ligation and SPRIselect bead cleanups according to the manufacturer’s protocol. Libraries were assessed on a TapeStation and sequenced as paired-end reads (~15 million paired-end reads per sample). Alignment of reads and data analysis were done as described previously^37^. After read alignment, we defined differential binding sites as reported in the ChIP–seq analysis section using DiffBind.

### CRISPR editing

CRISPR ribonucleoprotein (RNP) preparation and Neon electroporation conditions were based on previously optimized conditions^82^. Two single-guide RNAs (sgRNAs) were designed to disrupt exons to knock down TF genes. Similarly, two sgRNAs were designed to target and excise REs. The sgRNA sequences are available in Supplementary Table 7 and were used together at a 1:1 ratio. Chemically modified sgRNAs were synthesized (Integrative DNA Technologies), and CRISPR reagents and targeting vectors were delivered to cells using the Neon Electroporation Transfection System (Thermo Fisher Scientific). Reactions of Cas9–sgRNA RNP complexes were performed by combining recombinant Cas9 (Integrative DNA Technologies) and sgRNAs at a 1:3 molar ratio in Neon buffer R (Invitrogen), followed by incubation at room temperature for 10 min. RNP complexes were stored at 4 °C until use. Electroporation reactions were performed with 300,000 parental or GNE-7883-resistant H226 and MSTO cells, 2.5 pmol Cas9 and 7.5 pmol sgRNAs. Electroporation reactions were performed using 10-µl Neon tips with the following conditions: 1230 V, 10 ms pulse width, and 4 pulses. Cells were immediately placed into warm, antibiotic-free RPMI supplemented with fetal bovine serum and allowed to recover for 2 days. Resistant cells were cultured in medium containing 5 μM GNE-7883. RNA and genomic DNA were isolated from the cells 48 h following electroporation. Heterogeneous populations were used in qPCR measurements to assess mRNA and promoter transcript levels.

### DNA FISH

DNA FISH probes were designed following previously established methods. For each genomic locus, we designed oligonucleotide pools consisting of approximately 1,500 oligos, each containing a unique readout barcode sequence specific to that locus (Supplementary Table 8). DNA FISH imaging experiments were performed following the protocol described in ref. ^83^. Briefly, cells were fixed in 4% paraformaldehyde in 1× PBS for 10 min, followed by three PBS washes. Samples were then permeabilized with 0.5% (vol/vol) Triton X-100 for 10 min, treated with 0.1 M HCl for 5 min and washed in PBS. Hybridization was initiated by incubation in prehybridization buffer (2× saline sodium citrate (SSC) + 50% vol/vol formamide and 0.1% Tween) for 35 min at 42 °C, followed by addition of 1 μg of primary DNA probe diluted in 30 µl of hybridization buffer (2× SSC, 50% vol/vol formamide + 10% (vol/vol) dextran sulfate and 0.1% Tween). Samples were denatured on a heat block at 90 °C for 3 min (cultured cells) and hybridized overnight at 42 °C. Posthybridization washes were performed sequentially with prewarmed and room-temperature 2× SSC and stored in 2× SSC at 4 °C for up to 2 weeks. Genomic loci were visualized by hybridization of readout barcodes with complementary DNA oligonucleotide probes conjugated to Cy5 dye. Z-stacks (35 optical sections at 0.3-µm spacing) were acquired on a Nikon Ti-E microscope with NIS-Elements using a Plan Apo VC ×60 oil objective (NA 1.40), sampled at 0.1075 µm per pixel in XY. We implemented a fully automated pipeline to quantify nuclear puncta from Nikon ND2 image stacks. For each ND2 image, volumes were read with Bio-Formats (MATLAB). A maximum-intensity projection of images was generated and segmented with Cellpose (nuclei model; two-dimensional) using the following parameters: diameter = 100 px, flow_threshold = 0.4, cellprob_threshold = 1.0 and min_size = 70 px. The resulting two-dimensional mask was saved and replicated through Z to restrict downstream detection to nuclei. Spot detection was performed in three dimensions on the spot channel after Gaussian smoothing (*σxy* = 1.0 px, *σz* = 0) and a scale–space contrast enhancement computed as a difference of Gaussians (*σ*√2). The normalized response was thresholded at percentile 99.992, cleaned with h-maxima (*h* = 0.02), and intersected with regional maxima constrained by a spherical nonmaximum-suppression radius of 3 px. Candidate maxima were retained only inside nuclear masks and assigned to individual cells; per-cell spot counts were obtained by accumulation and written to CSV.

### Incucyte assay of cell growth

Cell proliferation following TF knockdown was measured by electroporation of 150,000 cells of each genotype with CRISPR guides, followed by plating in a 6-well plate. Following CRISPR engineering, cells were allowed to recover for 24 h before being transferred to an Incucyte ZOOM (Sartorius). Confluency analysis was performed using default settings every 6 h. Cell growth was recorded over a period of 7 days. Experiments were performed in six biological replicates for both parental and resistant cells, with nine measurement points across each well.

### Statistical analyses

Statistical analyses were performed in R or GraphPad Prism. qPCR measurements are presented as the mean ± s.d. of at least two biological replicates and two or more technical replicates, as stated in the figure legends. For results presented in bar graphs, significant changes were determined by two-tailed, unpaired *t*-tests at *P* < 0.05. Differential peaks from ChIP–seq were identified by DESeq2 (DiffBind) using FDR < 0.01. For box plot statistics, we used Wilcoxon rank-sum tests to assess significant differences in sequencing read coverage at *P* < 0.05 as specified in the figure legends. Differential gene expression analyses were performed with the following parameters: FDR < 0.01, log_2_FC > 1. Statistical parameters and numbers of repeats are reported in the figure legends.

### Reporting summary

Further information on research design is available in the Nature Portfolio Reporting Summary linked to this article.

## Online content

Any methods, additional references, Nature Portfolio reporting summaries, source data, extended data, supplementary information, acknowledgements, peer review information; details of author contributions and competing interests; and statements of data and code availability are available at 10.1038/s41594-026-01829-0.

## Supplementary information

Reporting Summary

Peer Review File

Supplementary Tables Supplementary Table 1: Differentially expressed genes in RNA-seq clusters from H226 cells. Supplementary Table 2: List of predicted upstream regulators of RNA-seq gene clusters. Supplementary Table 3: Differentially expressed genes in RNA-seq clusters from MSTO cells. Supplementary Table 4: Chromosomal coordinates of MCC-targeted gene promoters. Supplementary Table 5: List of differentially active promoters in H226 and MSTO cells. Supplementary Table 6: Expression of TFs in H226 cells from TFCheckpoint. Supplementary Table 7: List of primer and guide RNA sequences used in this study. Supplementary Table 8: Chromosomal locations of DNA FISH probes.

## Source data

Source Data Fig. 1 Statistical source data.

Source Data Fig. 2 Statistical source data.

Source Data Fig. 3 Statistical source data.

Source Data Fig. 4 Statistical Source Data

Source Data Fig. 5 Statistical source data.

Source Data Extended Data Fig. 1 Statistical source data.

Source Data Extended Data Fig. 1 Unprocessed DNA FISH images.

Source Data Extended Data Fig. 2 Statistical source data.

Source Data Extended Data Fig. 3 Statistical source data.

Source Data Extended Data Fig. 4 Unprocessed DNA FISH images.

Source Data Extended Data Fig. 5 Unprocessed DNA FISH images.

Source Data Extended Data Fig. 6 Statistical source data.

Source Data Extended Data Fig. 7 Statistical source data.

Source Data Extended Data Fig. 8 Statistical source data.

## Data Availability

All sequencing data are available under GEO accession code GSE248208 (TEAD1 and YAP CUT&RUN and RNA-seq data); BioProject ID PRJNA949402 (CTCF CUT&Tag data); and GEO accession code GSE288780 (datasets presented in this study). Source data are provided with this paper.

## Code availability

The custom MCC code developed for this study is available via GitHub at https://github.com/jhoover204/MCCTools/tree/v1.0.0.
