# Supplementary material for: Promoter reinforcement supports transcriptional resilience in drug-resistant cancer
Source: Nat Struct Mol Biol. 2026 Jul 6;33(7):1051–61. doi: 10.1038/s41594-026-01829-0 (PMC13372675; doi:10.1038/s41594-026-01829-0)
Supplement: Supplementary file 1 — Reporting Summary [file 41594_2026_1829_MOESM1_ESM.pdf]

nature portfolio

Corresponding author(s): Bence Daniel

Last updated by author(s): 04/14/2026

Reporting Summary

Nature Portfolio wishes to improve the reproducibility of the work that we publish. This form provides structure for consistency and transparency in reporting. For further information on Nature Portfolio policies, see our Editorial Policies and the Editorial Policy Checklist.

Statistics

For all statistical analyses, confirm that the following items are present in the figure legend, table legend, main text, or Methods section.

n/a	Confirmed	
☐	☒	The exact sample size (*n*) for each experimental group/condition, given as a discrete number and unit of measurement
☐	☒	A statement on whether measurements were taken from distinct samples or whether the same sample was measured repeatedly
☐	☒	The statistical test(s) used AND whether they are one- or two-sided<br>*Only common tests should be described solely by name; describe more complex techniques in the Methods section.*
☒	☐	A description of all covariates tested
☒	☐	A description of any assumptions or corrections, such as tests of normality and adjustment for multiple comparisons
☐	☒	A full description of the statistical parameters including central tendency (e.g. means) or other basic estimates (e.g. regression coefficient) AND variation (e.g. standard deviation) or associated estimates of uncertainty (e.g. confidence intervals)
☐	☒	For null hypothesis testing, the test statistic (e.g. *F*, *t*, *r*) with confidence intervals, effect sizes, degrees of freedom and *P* value noted<br>*Give P values as exact values whenever suitable.*
☒	☐	For Bayesian analysis, information on the choice of priors and Markov chain Monte Carlo settings
☒	☐	For hierarchical and complex designs, identification of the appropriate level for tests and full reporting of outcomes
☐	☒	Estimates of effect sizes (e.g. Cohen's *d*, Pearson's *r*), indicating how they were calculated

Our web collection on statistics for biologists contains articles on many of the points above.

Software and code

Policy information about availability of computer code

Data collection	Illumina NextSeq 2000; Illumina Novaseq X Plus
Data analysis	HTSeqGenie (R package version 4.30.0); Ingenuity Pathway Analyzer (Qiagen, Spring Release 2025); ENCODE ChIP-seq pipeline (v2.2.1); deepTools v.3.3.0; Custom MCC code: GitHub - https://github.com/jhoover204/MCCTools/tree/v1.0.0; TrimGalore (v0.6.10), Babraham Institute; BWA mem (v0.7.18); pairtools parse2 (v1.1.0); bedtools (v2.26).

For manuscripts utilizing custom algorithms or software that are central to the research but not yet described in published literature, software must be made available to editors and reviewers. We strongly encourage code deposition in a community repository (e.g. GitHub). See the Nature Portfolio guidelines for submitting code & software for further information.

Data

Policy information about availability of data

All manuscripts must include a data availability statement. This statement should provide the following information, where applicable:

- Accession codes, unique identifiers, or web links for publicly available datasets
- A description of any restrictions on data availability
- For clinical datasets or third party data, please ensure that the statement adheres to our policy

Data availability
All sequencing data is available under GEO accession numbers: GSE248208 - ChIP-seq and RNA-seq datasets, and GSE288780 - Micro-Capture C datasets.

Code Availability:
The custom Micro-Capture C code developed for this study is available on GitHub: link - https://github.com/jhoover204/MCCTools/tree/v1.0.0.

Research involving human participants, their data, or biological material

Policy information about studies with human participants or human data. See also policy information about sex, gender (identity/presentation), and sexual orientation and race, ethnicity and racism.

Reporting on sex and gender	n/a
Reporting on race, ethnicity, or other socially relevant groupings	n/a
Population characteristics	n/a
Recruitment	n/a
Ethics oversight	n/a

Note that full information on the approval of the study protocol must also be provided in the manuscript.

Field-specific reporting

Please select the one below that is the best fit for your research. If you are not sure, read the appropriate sections before making your selection.

☒ Life sciences ☐ Behavioural & social sciences ☐ Ecological, evolutionary & environmental sciences

For a reference copy of the document with all sections, see nature.com/documents/nr-reporting-summary-flat.pdf

Life sciences study design

All studies must disclose on these points even when the disclosure is negative.

Sample size	All RNA-seq experiments were performed in biological triplicates. ChIP-seq was performed in biological duplicates. Micro-Capture C was performed in two biological replicates, and in each biological replicate, two technical replicates were performed, each with three different T4DNA ligase concentrations to increase the complexity of the sequencing libraries. No specific statistical method was used to pre-determine sample size. Our sample size choice enabled robust detection of biological differences between conditions in all experimental settings. Covariates such as sequencing depth, library complexity, and potential batch effects were controlled through standardized experimental procedures and computational normalization. All samples were processed under consistent conditions to minimize technical variability.
Data exclusions	No data were excluded.
Replication	At least two biological replicates were performed in all experiments. All replication efforts were successful.
Randomization	Randomization was not applied, as samples were processed based on predefined experimental conditions and not subject to allocation bias.
Blinding	Blinding was not applied, as all experiments involved objective molecular readouts (e.g., sequencing-based assays) where investigator bias is not relevant.

Reporting for specific materials, systems and methods

We require information from authors about some types of materials, experimental systems and methods used in many studies. Here, indicate whether each material, system or method listed is relevant to your study. If you are not sure if a list item applies to your research, read the appropriate section before selecting a response.

Materials & experimental systems

n/a	Involved in the study	
☐	☒	Antibodies
☐	☒	Eukaryotic cell lines
☒	☐	Palaeontology and archaeology
☒	☐	Animals and other organisms
☒	☐	Clinical data
☒	☐	Dual use research of concern
☒	☐	Plants

Methods

n/a	Involved in the study	
☐	☒	ChIP-seq
☒	☐	Flow cytometry
☒	☐	MRI-based neuroimaging

Antibodies

Antibodies used	The following ChIP-seq antibodies have been used in the study: FOSL1 (PA5-66880 – Invitrogen - https://www.thermofisher.com/antibody/product/Fra1-Antibody-Polyclonal/PA5-66880); YAP (14074S – Cell Signaling- https://www.cellsignal.com/products/primary-antibodies/yap-d8h1x-xp-rabbit-mab/14074?srsltid=AfmBOooYy4Xz78p13soDZ0KR1UlBrDZhxtDocoai2F4pgZr0UCuHuSQN). For ChIP-qPCR, the following antibodies have been used: KLF4 (AF3158 – R&D Systems); NYFA (C15310261 – Diagenode).
Validation	These antibodies have been validated by the manufacturer, and have been extensively used in the literature in ChIP-seq or ChIP-qPCR applications.

Eukaryotic cell lines

Policy information about cell lines and Sex and Gender in Research

Cell line source(s)	The NCI-H226 cell line was obtained from ATCC. TEAD small molecule inhibitor (SMI) resistant NF2 null NCI-H226 were generated by incubation with increasing concentrations of TEAD SMI over time (0.25 µM to 3 µM at bi-weekly with 0.1-0.5 µM increments), ensuring at least a 25% confluence at all times. The generation of the resistant cell lines was done using pooled populations of survivor cells.
Authentication	Parental cell lines were authenticated by ATCC. Derived drug-resistant lines were not re-authenticated following generation.
Mycoplasma contamination	All cell lines are maintained by the internal cell line center (Cell Central) of Genentech Inc, and have been tested negative for Mycoplasma contamination.
Commonly misidentified lines (See ICLAC register)	None have been used.

Plants

Seed stocks	n/a
Novel plant genotypes	n/a
Authentication	n/a

ChIP-seq

Data deposition

☒ Confirm that both raw and final processed data have been deposited in a public database such as GEO.

☒ Confirm that you have deposited or provided access to graph files (e.g. BED files) for the called peaks.

Data access links *May remain private before publication.*	All sequencing data is public and available under GEO accession numbers: GSE248208 - ChIP-seq and RNA-seq datasets, and GSE288780 - Micro-Capture C datasets.<br>The custom Micro-Capture C code developed for this study is available on GitHub: link - https://github.com/jhoover204/MCCTools/tree/v1.0.0.
Files in database submission	RNA-seq<br>GSM7871934 Sensitive_DMSO_rep1<br>GSM7871935 Sensitive_DMSO_rep2<br>GSM7871936 Sensitive_DMSO_rep3<br>GSM7871937 Sensitive_GNE-7883_rep1<br>GSM7871938 Sensitive_GNE-7883_rep2<br>GSM7871939 Sensitive_GNE-7883_rep3<br>GSM7871940 Resistant_DMSO_rep1<br>GSM7871941 Resistant_DMSO_rep2<br>GSM7871942 Resistant_DMSO_rep3<br>GSM7871943 Resistant_GNE-7883_rep1<br>GSM7871944 Resistant_GNE-7883_rep2<br>GSM7871945 Resistant_GNE-7883_rep3<br>ChIP-seq<br>GSM8426979 H226_parental_FOSL1_rep1<br>GSM8426980 H226_parental_7883_FOSL1_rep1

GSM8426981 H226_resistant_7883_FOSL1_rep1
GSM8426982 H226_parental_YAP_rep1
GSM8426983 H226_parental_7883_YAP_rep1
GSM8426984 H226_resistant_7883_YAP_rep1
GSM8426985 H226_parental_FOSL1_rep2
GSM8426986 H226_parental_7883_FOSL1_rep2
GSM8426987 H226_resistant_7883_FOSL1_rep2
GSM8426988 H226_parental_YAP_rep2
GSM8426989 H226_parental_7883_YAP_rep2
GSM8426990 H226_resistant_7883_YAP_rep2
Micro-Capture C
GSM8775416 DMSO_treated_parental_H226_cells_rep1
GSM8775417 DMSO_treated_parental_H226_cells_rep2
GSM8775418 G7883_treated_parental_H226_cells_rep1
GSM8775419 G7883_treated_parental_H226_cells_rep2
GSM8775420 G7883_resistant_H226_cells_rep1
GSM8775421 G7883_resistant_H226_cells_rep2
New datasets:
GSM8775416 DMSO_treated_parental_H226_cells_rep1 (Micro-Capture C) Feb 12, 2025 approved None
GSM8775417 DMSO_treated_parental_H226_cells_rep2 (Micro-Capture C) Feb 12, 2025 approved None
GSM8775418 G7883_treated_parental_H226_cells_rep1 (Micro-Capture C) Feb 12, 2025 approved None
GSM8775419 G7883_treated_parental_H226_cells_rep2 (Micro-Capture C) Feb 12, 2025 approved None
GSM8775420 G7883_resistant_H226_cells_rep1 (Micro-Capture C) Feb 12, 2025 approved None
GSM8775421 G7883_resistant_H226_cells_rep2 (Micro-Capture C) Feb 12, 2025 approved None
GSM9385086 ATAC_DMSO_treated_parental_H226_cells_rep1 Dec 18, 2025 approved BW
GSM9385087 ATAC_DMSO_treated_parental_H226_cells_rep2 Dec 18, 2025 approved BW
GSM9385088 ATAC_G7883_treated_parental_H226_cells_rep1 Dec 18, 2025 approved BW
GSM9385089 ATAC_G7883_treated_parental_H226_cells_rep2 Dec 18, 2025 approved BW
GSM9385090 ATAC_G7883_resistant_H226_cells_rep1 Dec 18, 2025 approved BW
GSM9385091 ATAC_G7883_resistant_H226_cells_rep2 Dec 18, 2025 approved BW
GSM9385092 ATAC_DMSO_treated_parental_MSTO_cells_rep1 Dec 18, 2025 approved BW
GSM9385093 ATAC_DMSO_treated_parental_MSTO_cells_rep2 Dec 18, 2025 approved BW
GSM9385094 ATAC_G7883_treated_parental_MSTO_cells_rep1 Dec 18, 2025 approved BW
GSM9385095 ATAC_G7883_treated_parental_MSTO_cells_rep2 Dec 18, 2025 approved BW
GSM9385096 ATAC_G7883_resistant_MSTO_cells_rep1 Dec 18, 2025 approved BW
GSM9385097 ATAC_G7883_resistant_MSTO_cells_rep2 Dec 18, 2025 approved BW
GSM9385098 CUTRUN_H3K27ac_DMSO_treated_parental_H226_cells Dec 18, 2025 approved BW
GSM9385099 CUTRUN_H3K27ac_G7883_treated_parental_H226_cells Dec 18, 2025 approved BW
GSM9385100 CUTRUN_H3K27ac_G7883_resistant_H226_cells Dec 18, 2025 approved BW
GSM9385101 CUTRUN_H3K4me3_DMSO_treated_parental_H226_cells Dec 18, 2025 approved BW
GSM9385102 CUTRUN_H3K4me3_G7883_treated_parental_H226_cells Dec 18, 2025 approved BW
GSM9385103 CUTRUN_H3K4me3_G7883_resistant_H226_cells Dec 18, 2025 approved BW
GSM9385104 CTCF_DMSO_treated_parental_H226_cells_rep1 Dec 18, 2025 approved BW
GSM9385105 CTCF_DMSO_treated_parental_H226_cells_rep2 Dec 18, 2025 approved BW
GSM9385106 p300_DMSO_treated_parental_H226_cells_rep1 Dec 18, 2025 approved BW
GSM9385107 p300_DMSO_treated_parental_H226_cells_rep2 Dec 18, 2025 approved BW
GSM9385108 p300_G7883_treated_parental_H226_cells_rep1 Dec 18, 2025 approved BW
GSM9385109 p300_G7883_treated_parental_H226_cells_rep2 Dec 18, 2025 approved BW
GSM9385110 p300_G7883_resistant_H226_cells_rep1 Dec 18, 2025 approved BW
GSM9385111 p300_G7883_resistant_H226_cells_rep2 Dec 18, 2025 approved BW
GSM9385112 KLF4_DMSO_treated_parental_H226_cells_rep1 Dec 18, 2025 approved BW
GSM9385113 KLF4_DMSO_treated_parental_H226_cells_rep2 Dec 18, 2025 approved BW
GSM9385114 KLF4_G7883_treated_parental_H226_cells_rep1 Dec 18, 2025 approved BW
GSM9385115 KLF4_G7883_treated_parental_H226_cells_rep2 Dec 18, 2025 approved BW
GSM9385116 KLF4_G7883_resistant_H226_cells_rep1 Dec 18, 2025 approved BW
GSM9385117 KLF4_G7883_resistant_H226_cells_rep2 Dec 18, 2025 approved BW
GSM9385118 YAP_DMSO_treated_parental_MSTO_cells_rep1 Dec 18, 2025 approved BW
GSM9385119 YAP_DMSO_treated_parental_MSTO_cells_rep2 Dec 18, 2025 approved BW
GSM9385120 YAP_G7883_treated_parental_MSTO_cells_rep1 Dec 18, 2025 approved BW
GSM9385121 YAP_G7883_treated_parental_MSTO_cells_rep2 Dec 18, 2025 approved BW
GSM9385122 YAP_G7883_resistant_MSTO_cells_rep1 Dec 18, 2025 approved BW
GSM9385123 YAP_G7883_resistant_MSTO_cells_rep2 Dec 18, 2025 approved BW
GSM9385124 DMSO_treated_parental_MSTO_cells_rep1 Dec 18, 2025 approved BW
GSM9385125 G7883_treated_parental_MSTO_cells_rep1 Dec 18, 2025 approved BW
GSM9385126 G7883_resistant_MSTO_cells_rep1 Dec 18, 2025 approved BW
GSM9385127 DMSO_treated_parental_MSTO_cells_rep2 Dec 18, 2025 approved None
GSM9385128 G7883_treated_parental_MSTO_cells_rep2 Dec 18, 2025 approved None
GSM9385129 G7883_resistant_MSTO_cells_rep2 Dec 18, 2025 approved None
GSM9385130 DMSO_treated_parental_H226_cells_rep1 Dec 18, 2025 approved BW
GSM9385131 G7883_treated_parental_H226_cells_rep1 Dec 18, 2025 approved BW
GSM9385132 G7883_resistant_H226_cells_rep1 Dec 18, 2025 approved BW
GSM9385133 DMSO_treated_parental_H226_cells_rep2 Dec 18, 2025 approved None
GSM9385134 G7883_treated_parental_H226_cells_rep2 Dec 18, 2025 approved None
GSM9385135 G7883_resistant_H226_cells_rep2 Dec 18, 2025 approved None
GSM9385136 RNAseq_DMSO_treated_parental_MSTO_cells_rep1 Dec 18, 2025 approved BW
GSM9385137 RNAseq_DMSO_treated_parental_MSTO_cells_rep2 Dec 18, 2025 approved BW

GSM9385138	RNAseq_DMSO_treated_parental_MSTO_cells_rep3 Dec 18, 2025 approved BW
GSM9385139	RNAseq_G7883_treated_parental_MSTO_cells_rep1 Dec 18, 2025 approved BW
GSM9385140	RNAseq_G7883_treated_parental_MSTO_cells_rep2 Dec 18, 2025 approved BW
GSM9385141	RNAseq_G7883_treated_parental_MSTO_cells_rep3 Dec 18, 2025 approved BW
GSM9385142	RNAseq_G7883_resistant_MSTO_cells_rep1 Dec 18, 2025 approved BW
GSM9385143	RNAseq_G7883_resistant_MSTO_cells_rep2 Dec 18, 2025 approved BW
GSM9385144	RNAseq_G7883_resistant_MSTO_cells_rep3 Dec 18, 2025 approved BW
GSM9385145	RNAseq_Cas9_treated_parental_H226_cells_rep1 Dec 18, 2025 approved BW
GSM9385146	RNAseq_Cas9_treated_parental_H226_cells_rep2 Dec 18, 2025 approved BW
GSM9385147	RNAseq_YAPTAZKD_treated_parental_H226_cells_rep1 Dec 18, 2025 approved BW
GSM9385148	RNAseq_YAPTAZKD_treated_parental_H226_cells_rep2 Dec 18, 2025 approved BW
GSM9385149	RNAseq_panTEADKD_treated_parental_H226_cells_rep1 Dec 18, 2025 approved BW
GSM9385150	RNAseq_panTEADKD_treated_parental_H226_cells_rep2 Dec 18, 2025 approved BW

Genome browser session (e.g. UCSC)

We provided an IGV session .xml file (IGV_session_NSMB_A50953.xml) that accesses a remote Dropbox folder that contains all bigwig files. This file can be loaded in IGV by clicking on File - Open Session and selecting this file to open the session. Additionally, this session can be also loaded via the online IGV Web Application on this link: https://igv.org/app/ - On this site, navigate to Session - Local file... and open the provided .xml file.

Methodology

Replicates

RNA-seq was performed in three biological replicates. ChIP-seq, ATAC-seq was performed in two biological replicates. Micro-Capture C was performed in 2 biological replicates, and in each biological replicate, six technical replicates were performed using slightly different ligase conditions to increase library complexity.

Sequencing depth

RNA-seq and ChIP-seq libraries have been sequenced to ~25 million depth with paired-end sequencing. In Micro-Capture C experiments, we aimed to sequence at least 75-80 million pared-end reads per condition.

Antibodies

The following ChIP-seq antibodies have been used in the study: FOSL1 (PA5-66880 – Invitrogen - https://www.thermofisher.com/antibody/product/Fra1-Antibody-Polyclonal/PA5-66880); YAP (14074S – Cell Signaling- https://www.cellsignal.com/products/primary-antibodies/yap-d8h1x-xp-rabbit-mab/14074?srsltid=AfmBOooYy4Xz78p13soDZ0KR1UlBrDZhxtDocoai2F4pgZr0UCuHuSQN). For ChIP-qPCR, the following antibodies have been used: KLF4 (AF3158 – R&D Systems); NYFA (C15310261 – Diagenode).

Peak calling parameters

Peaks were called with the SPP peak caller with and IDR threshold of 0.05. For MCC, custom peak calling parameters were used that are detailed in the methods section, see below too: For each capture viewpoint, signal within ±1 Mb of the bait midpoint was extracted, excluding the central ±2.5 kb to avoid local ligation artifacts. The bigWig-derived signal arrays were then processed using custom Python code built on NumPy, including Gaussian smoothing (σ = 1 bin), computation of a local background via a 10 kb running median, and calculation of median absolute deviations (MAD) for thresholding. Candidate MCC peaks were defined as regions with signal ≥3.5 × MAD above the local baseline for at least 3 consecutive bins (≥150 bp). Adjacent candidates separated by ≤150 bp were merged, and broad plateaus longer than 1kb were subdivided at local valleys where the signal dropped by ≥40% relative to the local maximum. To increase biological specificity, MCC peaks were further filtered to retain only those overlapping accessible chromatin regions from matched ATAC-seq profiles.

Data quality

Pearson correlation analysis showed high concordance across replicates ~0.8-0.9 correlation coefficient. Additionally, ChIP-seq, ATAC-seq and RNA-seq libraries showed low percentage of duplicate reads ~10%, and good percentage of sequencing fragments falling on called peaks ~50-60%.

Software

ChIP-seq results were analyzed using the ENCODE ChIP-seq pipeline (v2.2.1)66. ChIP-seq reads were aligned to the human reference genome (hg38) using Bowtie2 (v2.3.4.3)67. Aligned reads were then filtered for quality and duplicates using samtools (v1.9)68 and Picard (Broad Institute - v2.20.7). The SPP peak caller was used to call ChIP-seq peaks for FOSL1 and YAP, and input was used to assess the background of the experiments69. Peak sets were filtered using a list of genomic regions that contain anomalous, unstructured, or experiment independent high signals. ChIP-seq bam and bed files were then used to call differential peaks by using DiffBind (v3.12.0)70,71. Briefly, the Differential Binding Analysis (DBA) object was created by loading the bam and bed files with the dba() function. Reads were counted with the dba.count() function, followed by depth normalization using the dba.normalize() function, and differential peaks were called across the conditions with the dba.analyze() function using DESeq2 and the following statistical parameters (FDR<0.01).
