# Supplementary figures and images for: Promoter reinforcement supports transcriptional resilience in drug-resistant cancer
Source: Nat Struct Mol Biol. 2026 Jul 6;33(7):1051–61. doi: 10.1038/s41594-026-01829-0 (PMC13372675; doi:10.1038/s41594-026-01829-0)

## Extended Data Fig. 1g

H226 P-DMSO YAP1

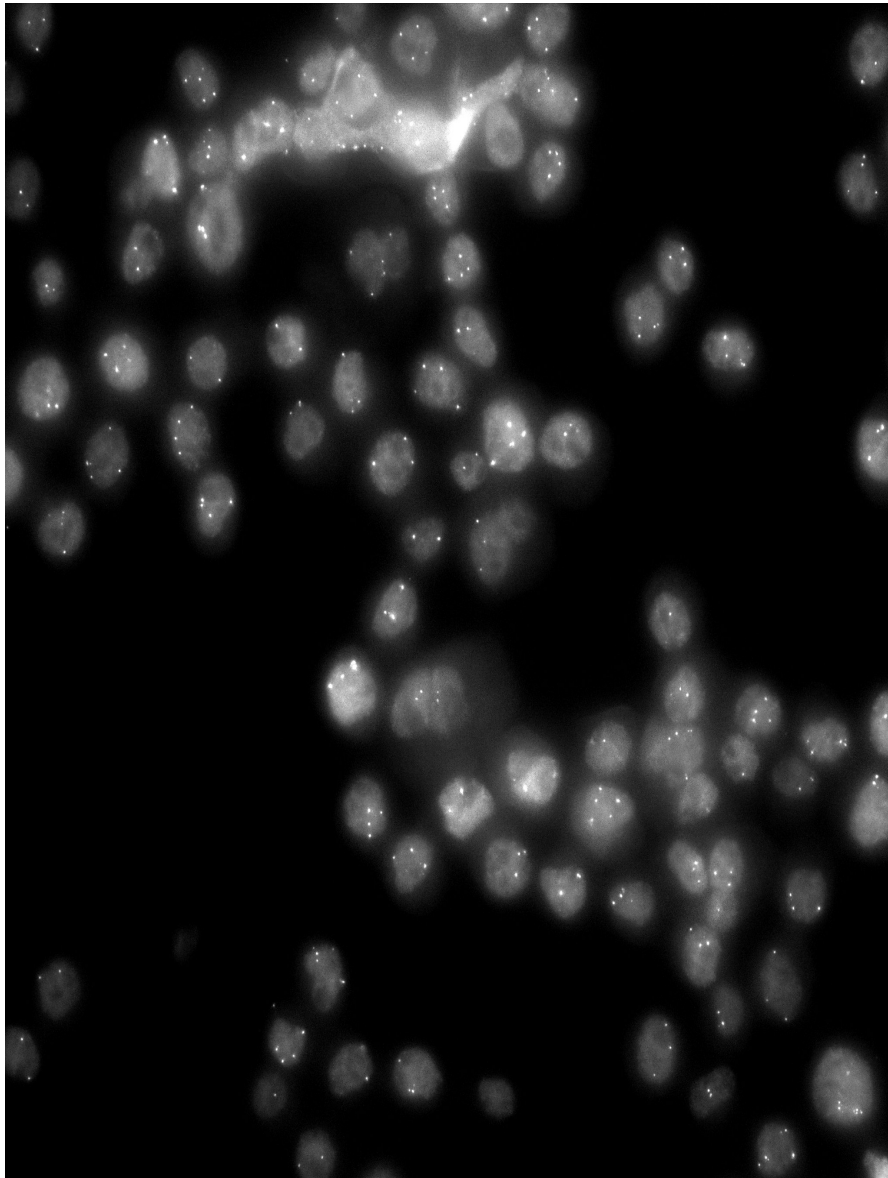

H226 R-G7883 YAP1

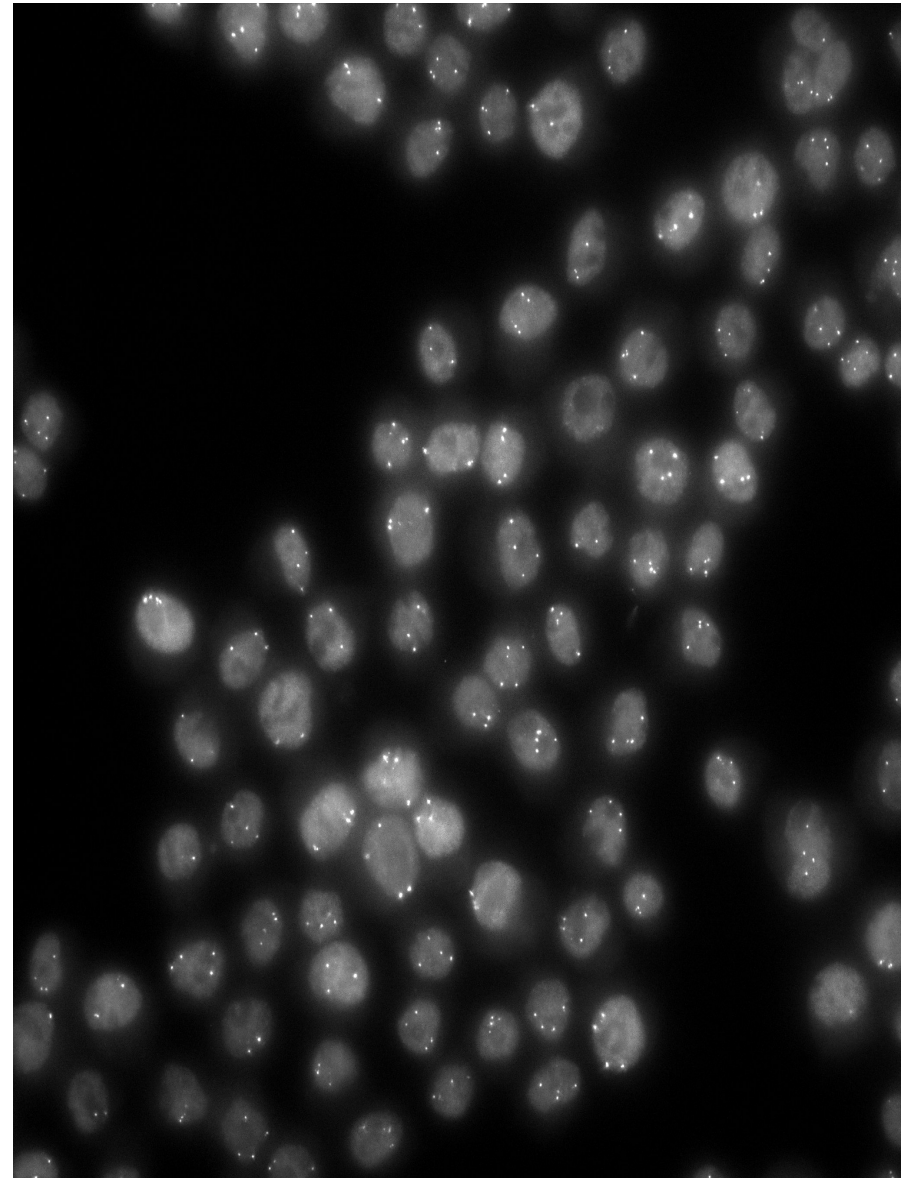

## Extended Data Fig. 1g

MSTO P-DMSO YAP1

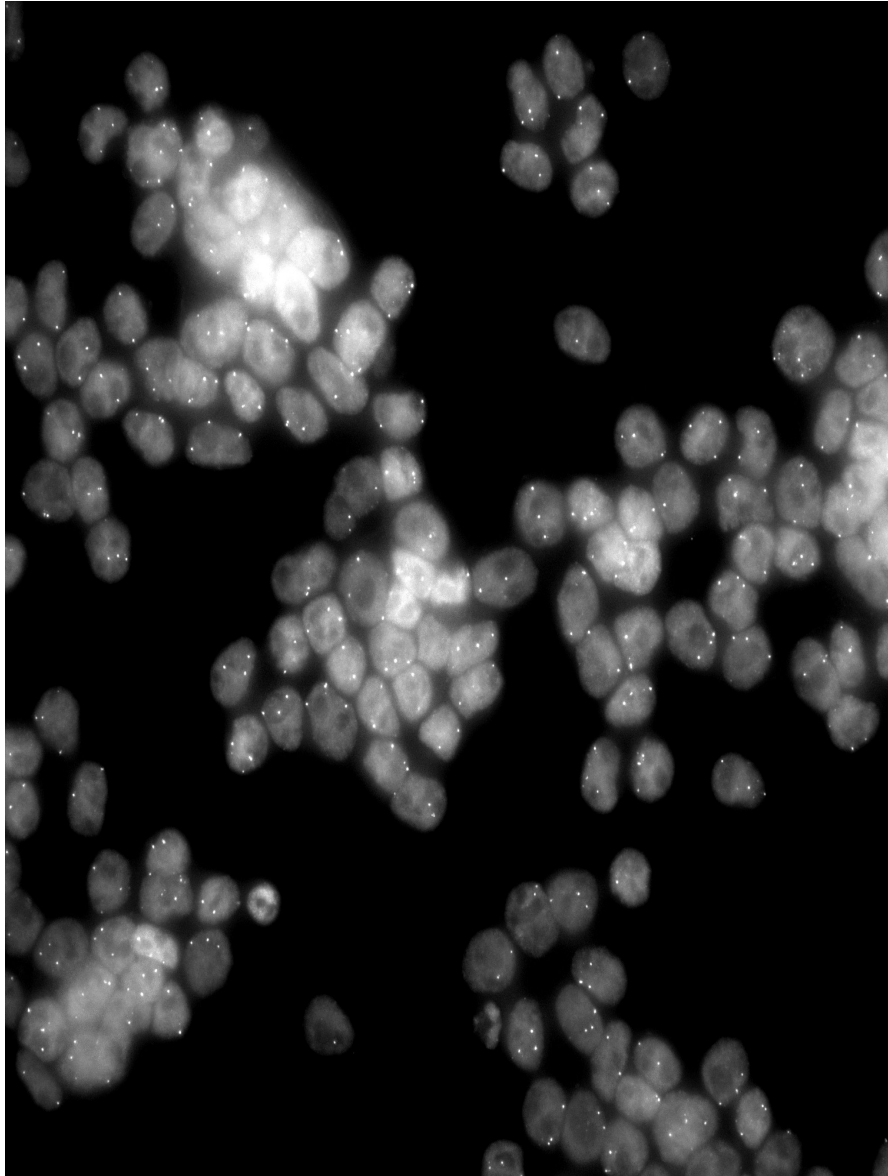

MSTO R-G7883 YAP1

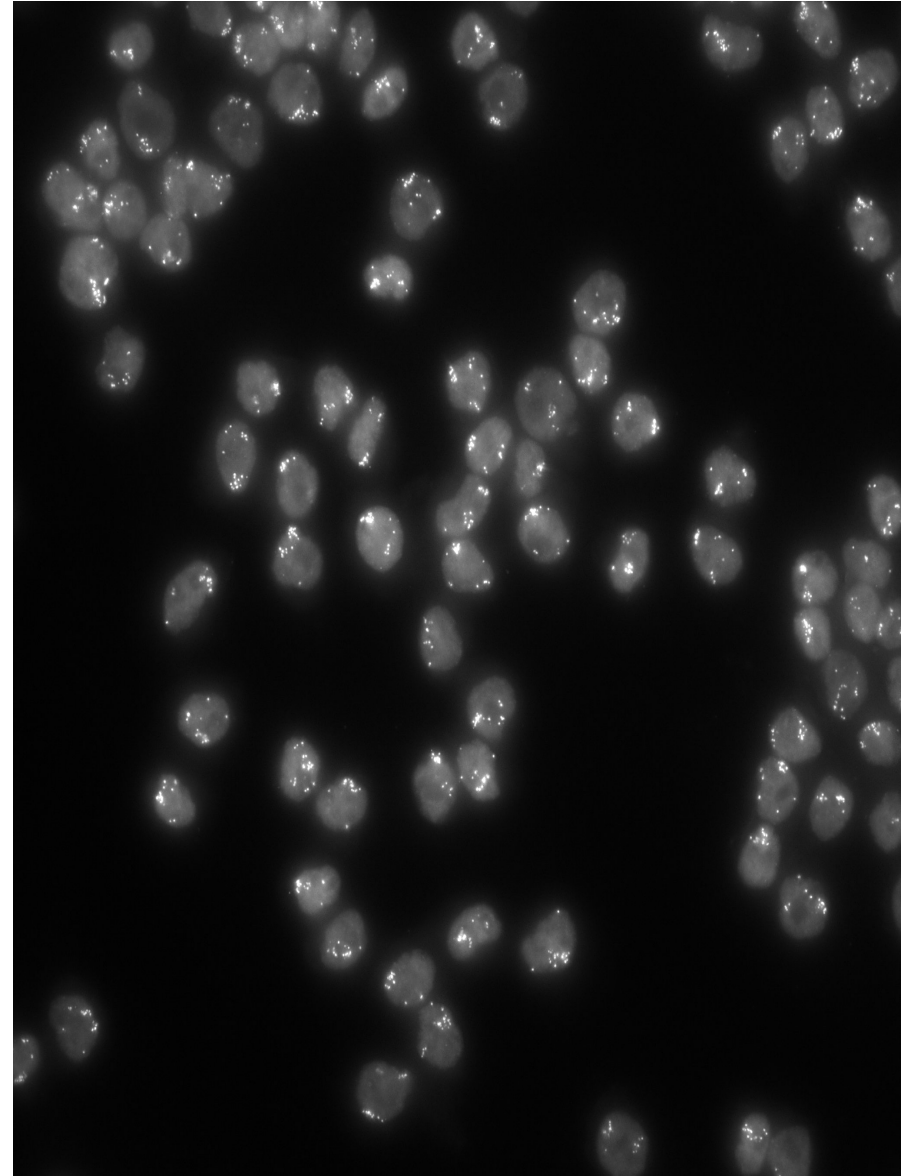

Supplement: Supplementary file 10 — Unprocessed DNA FISH images. [file 41594_2026_1829_MOESM10_ESM.pdf]

## Extended Data Fig. 4a

H226 P-DMSO FOSL1

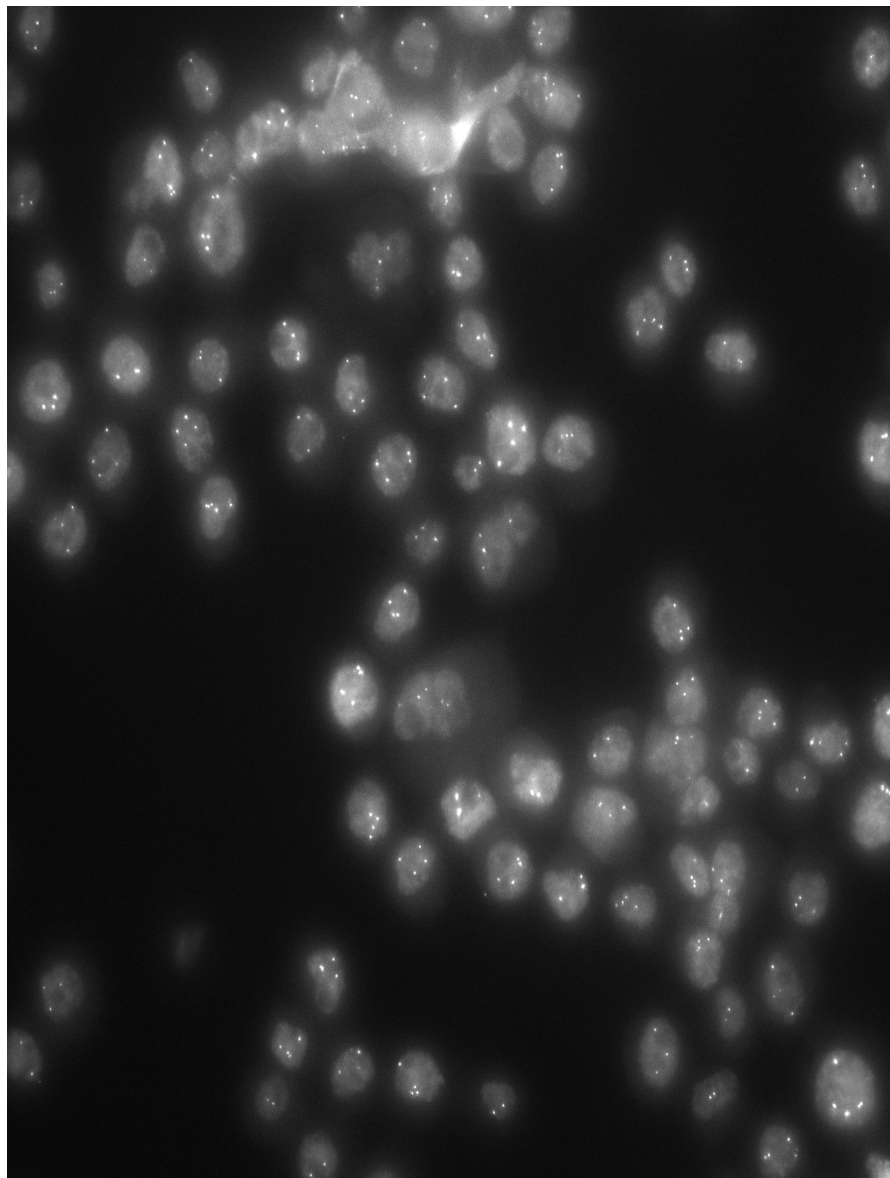

H226 R-G7883 FOSL1

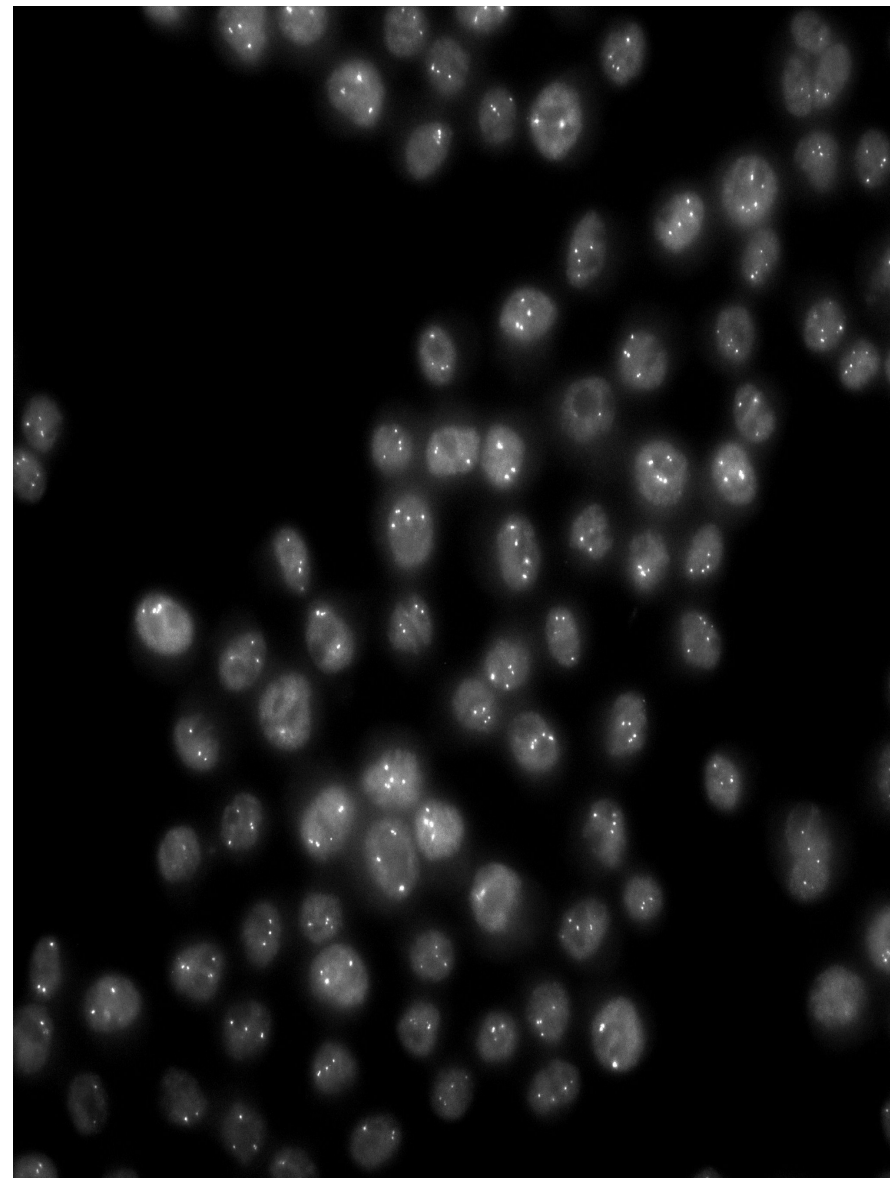

Supplement: Supplementary file 13 — Unprocessed DNA FISH images. [file 41594_2026_1829_MOESM13_ESM.pdf]

## Extended Data Fig. 5d

H226 P-DMSO KLF4

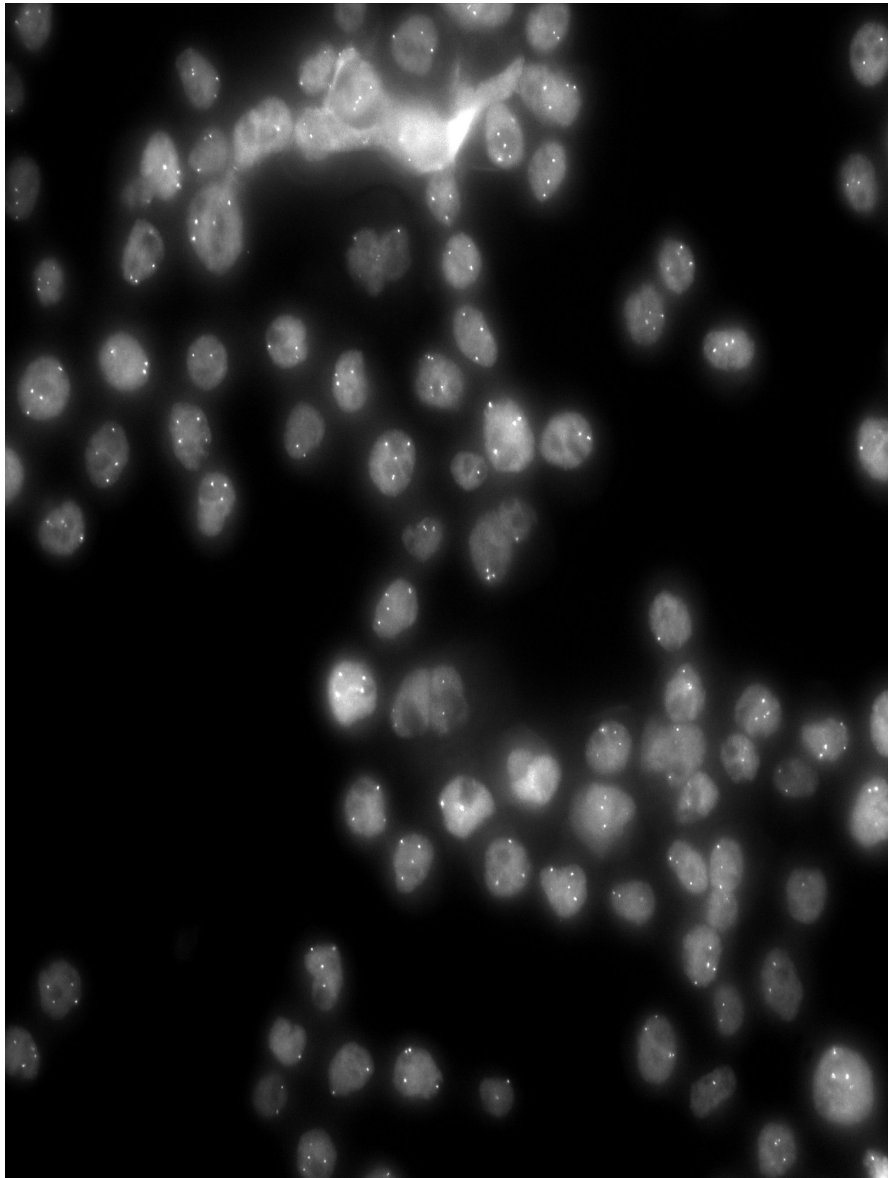

H226 R-G7883 KLF4

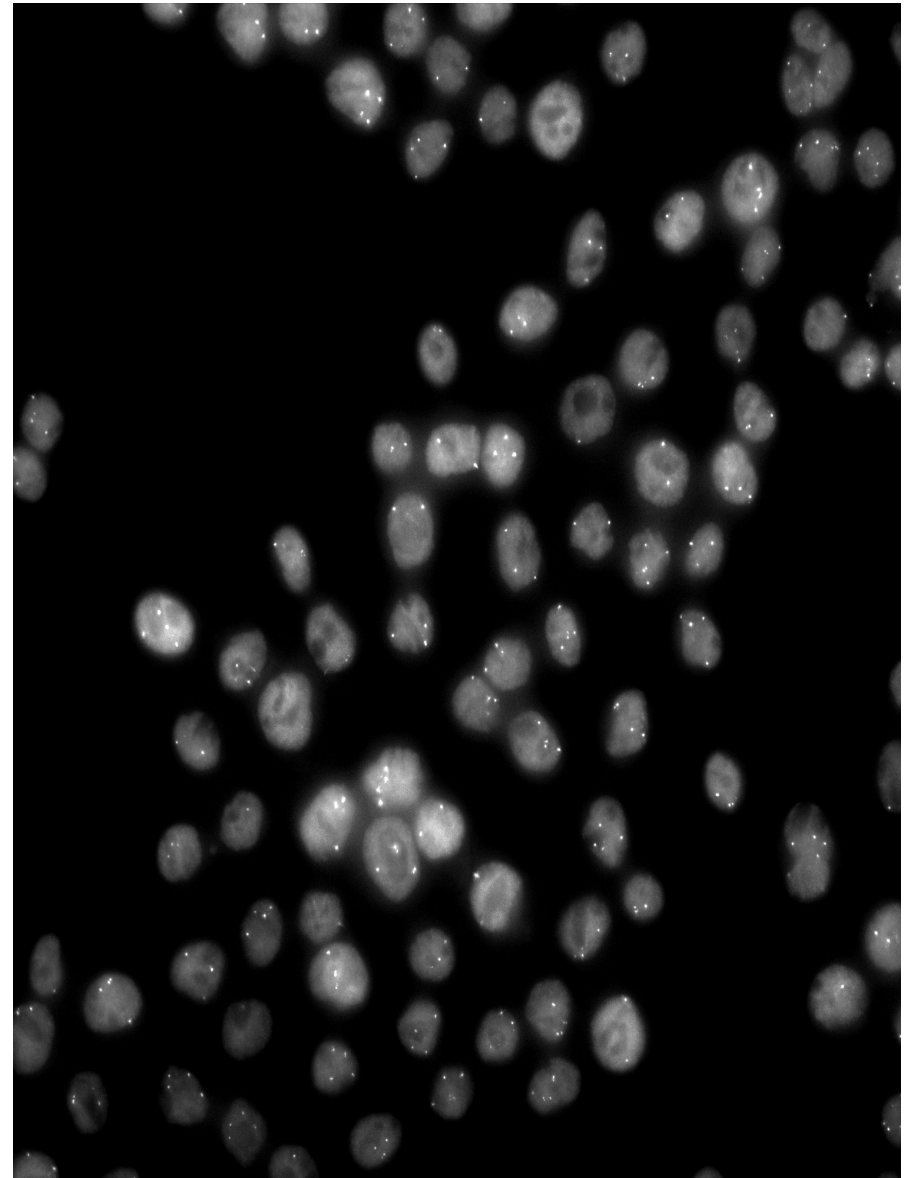

Supplement: Supplementary file 14 — Unprocessed DNA FISH images. [file 41594_2026_1829_MOESM14_ESM.pdf]
