# Supplementary material for: Promoter reinforcement supports transcriptional resilience in drug-resistant cancer
Source: Nat Struct Mol Biol. 2026 Jul 6;33(7):1051–61. doi: 10.1038/s41594-026-01829-0 (PMC13372675; doi:10.1038/s41594-026-01829-0)
Supplement: Supplementary file 2 — Peer Review File [file 41594_2026_1829_MOESM2_ESM.pdf]

nature portfolio

Peer Review File

Promoter reinforcement supports transcriptional resilience in drug-resistant cancer

Corresponding Author: Dr Bence Daniel

Version 0:

Decision Letter:

13th Jun 2025

Dear Dr Daniel,

Thank you again for submitting your manuscript "Promoter-centric gene regulation in drug-resistant cancer". I apologize for the delay in responding, which resulted from the difficulty in obtaining referee reports. We now have comments (below) from the 3 reviewers who evaluated your paper. In light of those reports, we remain interested in your study and would like to see your response to the comments of the referees, in the form of a revised manuscript.

You will see that the reviewers found the dataset interesting but had significant concerns about the depth of analyses and mechanistic understanding, strength of the conclusions, and generalizability, among other concerns. In our view, the referees' concerns point to a dataset that is premature to support the conclusions. The reviewers' remarks would need to be addressed thoroughly experimentally to gather sufficient support for the conclusions and for publication, and reconsideration of the study for this journal and re-engagement of referees will depend on the strength of these revisions.

To guide the scope of the revisions, we list below a prioritized set of referee points that should be addressed in the revision, which we hope will be helpful to you. Please do not hesitate to contact me directly if you would like to discuss these issues further.

-- The reviewers asked for additional evidence supporting the model, including the roles of particular TFs in binding and enhancing promoter activity in resistant cells, and data documenting and supporting changes in enhancer and promoter activity. These points should be addressed in full:

Rev#1 points #2, #3, #5, #7, #8
Rev#2 point #1
Rev#3 points #1, #2, #3

-- We agree with the reviewers that addressing the generalizability of the observations (using different cell models and extending the loci studied), as guided by the reviewers, will be important:

Rev#1 point #6
Rev#2 points #2, #3
Rev#3 point #4

-- Lastly, it is essential to confirm that the mechanism presented is important and causal in drug resistance:
Rev#1 point #4
Rev#2 point #4
Rev#3 point #5

-- Please also address the reviewers' technical comments, minor points, requests for controls, clarifications, edits, or discussion.

Our standard revision period is six months, and we are committed to providing a fair and constructive peer-review process. Please do not hesitate to contact me if you would like to discuss the reviews or anticipate any issues or delays addressing the reviews.

We appreciate the requested revisions are extensive. If you cannot send your revision within this time, please let us know.

We will be happy to consider your revision as long as nothing similar has been accepted for publication at NSMB or published elsewhere. Should your manuscript be substantially delayed without notifying us in advance and your article is eventually published, the received date would be that of the revised, not the original, version.

As you already know, we put great emphasis on ensuring that the methods and statistics reported in our papers are correct and accurate. As such, if there are any changes that should be reported, please submit an updated version of the Reporting Summary along with your revision.

Reporting Summary:
https://www.nature.com/documents/nr-reporting-summary.pdf

Please note that the form is a dynamic 'smart pdf' and must therefore be downloaded and completed in Adobe Reader.

When submitting the revised version of your manuscript, please pay close attention to our href="https://www.nature.com/nature-portfolio/editorial-policies/image-integrity">Digital Image Integrity Guidelines. and to the following points below:

-- that unprocessed scans are clearly labelled and match the gels and western blots presented in figures. Please note that all key data shown in the main figures as cropped gels or blots should be presented in uncropped form, with molecular weight markers. While these data can be displayed in a relatively informal style, they must refer back to the relevant figures. These data should be submitted as source data with the last revision, prior to acceptance.
-- that control panels for gels and western blots are appropriately described as loading on sample processing controls
-- all images in the paper are checked for duplication of panels and for splicing of gel lanes.
-- For any revision that includes light microscopy data, we ask our authors to please include a completed light microscopy reporting table [https://www.nature.com/documents/Light_microscopy_reporting_table.xlsx] to ensure the methods are described thoroughly. The table will be available to reviewers and ultimately published should the manuscript be accepted at the journal.

EXTENDED DATA FIGURES

When re-submitting your manuscript, please ensure that any supplementary figures and tables that are crucial to the manuscript's conclusions are converted into Extended Data figures and tables to increase visibility of these data. Extended Data figures and tables are online-only (present in the online PDF and full-text HTML versions of the paper), peer-reviewed display items that provide essential background to the article but are not included in the main article due to space constraints. A maximum of ten Extended Data display items (figures and tables) is permitted.

Finally, please ensure that you retain unprocessed data and metadata files after publication, ideally archiving data in perpetuity, as these may be requested during the peer review and production process or after publication if any issues arise.

If there are additional or modified structures presented in the final revision, please submit the corresponding PDB validation reports.

SOURCE DATA: we urge authors to provide, in tabular form, the data underlying the graphical representations used in figures. This is to further increase transparency in data reporting, as detailed in this editorial (http://www.nature.com/nsmb/journal/v22/n10/full/nsmb.3110.html). Spreadsheets can be submitted in excel format. Only one (1) file per figure is permitted; thus, for multi-paneled figures, the source data for each panel should be clearly labeled in the Excel file; alternately the data can be provided as multiple, clearly labeled sheets in an Excel file. When submitting files, the title field should indicate which figure the source data pertains to. We encourage our authors to provide source data at the revision stage, so that they are part of the peer-review process.

While we encourage the use of color in preparing figures, please note that this will incur a charge to partially defray the cost of printing. Information about color charges can be found at http://www.nature.com/nsmb/authors/submit/index.html#costs

We require deposition of coordinates (and, in the case of crystal structures, structure factors) into the Protein Data Bank with the designation of immediate release upon publication (HPUB). Electron microscopy-derived density maps and coordinate data must be deposited in EMDB and released upon publication. Deposition and immediate release of NMR chemical shift assignments are highly encouraged. Deposition of deep sequencing and microarray data is mandatory, and the datasets must be released prior to or upon publication. To avoid delays in publication, dataset accession numbers must be supplied with the final accepted manuscript and appropriate release dates must be indicated at the galley proof stage. Please find the complete NRG policies on data availability at http://www.nature.com/authors/policies/availability.html.

Nature Structural & Molecular Biology is committed to improving transparency in authorship. As part of our efforts in this direction, we are now requesting that all authors identified as 'corresponding author' on published papers create and link their Open Researcher and Contributor Identifier (ORCID) with their account on the Manuscript Tracking System (MTS), prior to acceptance. This applies to primary research papers only. ORCID helps the scientific community achieve unambiguous attribution of all scholarly contributions. You can create and link your ORCID from the home page of the MTS by clicking on 'Modify my Springer Nature account'. For more information please visit please visit <a

href="http://www.springernature.com/orcid">www.springernature.com/orcid.

Please use the link below to submit your revised manuscript and related files:

Link Redacted

Note: This URL links to your confidential home page and associated information about manuscripts you may have submitted, or that you are reviewing for us. If you wish to forward this email to co-authors, please delete the link to your homepage.

We look forward to seeing the revised manuscript and thank you for the opportunity to review your work.

Sincerely,

Melina Casadio, PhD
Locum Chief Editor, Nature Structural & Molecular Biology
ORCID ID: https://orcid.org/0000-0003-2389-2243

Referee expertise:

Referee #1: 3D genome, genomics, gene regulation

Referee #2: cancer epigenetics

Referee #3: 3D genome, genomics, gene regulation

Reviewers' Comments:

Reviewer #1 (Remarks to the Author):

In this manuscript, Kameswaran and colleagues investigate how 3D cis-regulatory landscapes of cancer cells (mesothelioma cancer cells; NCI-H226) change in response to a drug targeting the transcription factor TEAD. They compare both an acute and prolonged drug treatment in which the cells have become resistant to the drug. Interestingly, they discover that interactions between gene promoters and distal regulatory elements remain reduced in resistant cells despite recovery of gene expression levels. Editing of these elements shows that the resistant cells have become independent of these elements. Based on analyses of transcription factor binding and promoter activity, the authors propose that these genes have become more dependent on regulation via their promoter and that this “promoter-switch” may be a common mechanism for induced resistance to drugs targeting transcription factors in cancer cells.

This manuscript is clear and well-written and presents very interesting data that contribute to our mechanistic understanding of how the regulatory landscapes of cancer cells adapt to drug treatments. Although it remains unclear how generalizable these findings are, I find these findings in principle sufficiently interesting to justify publication in NSMB. However, at the moment, the conclusions are in my view not yet fully supported by the data. I have the following suggestions for the authors to improve their manuscript:

Major comments

1. I find it curious that YAP binding levels at distal elements seems to increase in resistant cells, even though these elements become less functional. Can the authors comment on this observation?
2. The MCC data are poorly annotated. It has been shown in the original MCC paper (Hua et al. Nature 2021) that promoters frequently interact with both enhancers, CTCF-binding sites, and other promoters. In this manuscript, it is implied that all MCC peaks represent enhancer-promoter interactions, but this is difficult to judge from the data. It would be really helpful if the authors would also show CTCF tracks – even if only for the parental cell line. A better classification and quantification (see below) of the MCC interactions might help the authors to better interpret why not all loci respond in the same way to treatment/resistance.
3. The quantification of the MCC data is not very thorough. The authors only show enrichment at AP-1 motifs in Figure 2C. This seems a bit arbitrary. An advantage of the MCC data is that they can be peak-called. The peaks can then be classified in different categories and easily quantified. This strategy seems more robust and has been used in previous publications with MCC data (scripts should be available, e.g. in Hua et al. Nature 2021 or Karpinska et al. NSMB 2025). It should be noted that the quality of the MCC data in this manuscript seems substantially lower than in previous publications, so it would be important that the authors carefully tune their peal call / quantification strategy.
4. A strong part of the paper is that the authors perform genome editing to perturb the distal elements to demonstrate that the selected loci are no longer dependent on these elements for their expression in the resistant cells. However, since the authors propose that the independence of the elements is a mechanism of resistance, it would be very interesting to know if/how the “acutely” treated cells (P-G7883) depend on these elements.
5. The FOSL1 binding distribution in resistant cells in Figure 4D seems different compared to the other conditions (bimodal?). Does this relate to some of the observed differences in the loci of interest? E.g., do the genes that lose most

distal MCC interactions, gain most FOSL1 at the promoter and/or vice versa?
6. Since the authors propose that KLF4 has a key role in coordinating the promoter-driven gene expression in resistant cells, it would be helpful if the authors would assess this genome-wide via ChIP-seq analysis rather than ChIP-qPCR at a handful of selected genes.
7. It would also be helpful if the authors could include the FOS1 and KLF4 ChIP-seq data directly under the MCC data in the example panels so the readers can more easily/comprehensively interpret all described regulatory changes at loci of interest.
8. Related to comment 3: the MCC data are not very deep / a bit noisy. The quality generally seems sufficient for the analyses of the authors, but the MCC footprinting is not very convincing in my opinion and I think it would be better if the authors would de-emphasize these analyses.

Minor comments

1. Regarding the first sentence of the abstract: not all eukaryotic cells contain distal regulatory elements (e.g., yeast does not). Regarding the first sentence of the introduction, promoters should not be included in the listed “long-range regulatory elements”.
2. It would be helpful if the R-G7883 cells were better described in the main text. Since these cells are crucial to understand the main message of the paper, it would be nice if readers do not have to read another paper to understand the nature of these cells and how they were generated.
3. Line188: The interactions in the ANKRD1 locus are not “fully” restored.

Reviewer #2 (Remarks to the Author):

This manuscript by Kameswaran and colleagues investigates characteristics of a mesothelioma cell line with induced resistance to GNE-7883, a pan-TEAD inhibitor. The primary observation is that when mesothelioma cells develop resistance to GNE-7883 they have reduced activity of some enhancer sites enriched for binding of proteins relevant to the pathway that is being inhibited. A team that includes authors of the present study have previously published that GNE-7883 treatment remodels chromatin in OVCAR-8 cells, closing it at distal regions that are enriched for TEAD motifs and opening it at promoters of upregulated genes, with most significant transcriptional effects on YAP/TAZ targets (Hagenbeek et al 2023). This work extends that observation through additional epigenomic assays and functional studies demonstrating the reduced role of specific enhancer sites.

Strengths of the study include the use of a newly developed chemical agent that impacts activity of a clinically important pathway, and the use of the MCC assay to evaluate promoter-enhancer contacts. The experimental data are presented clearly. However, there are several weaknesses in the present study that preclude my recommending it for publication in its present form.

1) Tumor progression and adaptation to therapy is often accompanied by reorganization of enhancer activity (e.g. Roe Cell 2017, Liu JCI 2021). Are the investigators seeing a loss of enhancer activity because they are specifically focusing on the targets of GNE-7883 in cells that have adapted to TEADi, or is this a general phenomenon affecting enhancers of many other genes? It would be logical to perform ATAC-seq and ChIP for H3K27ac marks in the three cell states to demonstrate that elimination of enhancer-promoter contacts are linked to general changes in chromatin conformation. For unbiased assessment of promoter activity, it would be appropriate for the investigators to use computational analysis of RNA-seq data contrasting the three main conditions (e.g. using methods published in PMID 31491388) to evaluate whether differential promoter activity is enriched at genes where enhancer-promoter contacts have been measured to be different.

2) The authors suggest they have identified a general principle of the elimination of enhancer site activity during therapy resistance from a single targeted drug evaluated in a single cell line. It would be important to show that the proposed adaptation of promoter-centric response as a therapy resistance mechanism is not a specific feature of NCI-H226 and GNE-7883 cells by demonstrating that these results can be observed in additional cell line models.

3) The causal inferences drawn from the study are in some cases not justified by the observations. For example: "Our study introduces the idea that promoters can serve as central regulators of gene expression in drug resistant cancer cells"; this statement implies that this study has discovered that regulation at gene promoters is the key determinant of transcriptional activity. The statement "most promoter elements in resistant cells exist within a dysregulated 3D chromatin configuration compared to parental cells, where REs and promoters are uncoupled in the 3D space" is hard to justify given the targeted nature of the chromatin conformation analysis. The statement "in the resistant state, multiple genes’ expression was more sensitive to perturbation of the promoter-specific TF, NFYA (e.g., ANKRD1, CCN2, ETV4, FOSL1, VIM), suggesting that promoter-driven gene expression is favored in resistant cells" makes a general assertion about the role of promoter-driven gene expression at all genes, when only a handful of genes are evaluated using targeted assays.

4) It is unclear whether the observed differences in enhancer usage at specific sites are important for the development of resistance to TEADi therapy. The most straightforward interpretation is the one already present in Hagenbeek et al; that these enhancer sites are important for normal functioning of the tumor cells and contain binding sites that are disfavored when cells have adapted to TEADi treatment.

5) I don't understand the scale being used for mRNA expression levels in figure 3.

Reviewer #2 (Remarks on code availability):

I made a quick review of the MCCTools github site. The documentation appears to be professional and mature, with hallmarks of an experienced developer. I did not attempt to download or run the code.

Reviewer #3 (Remarks to the Author):

In the manuscript “Promoter-centric gene regulation in drug-resistant cancer”, Vasumathi Kameswaran and colleagues investigate the mechanisms by which gene regulation is reconfigured in mesothelioma cancer cell lines that were developed to acquire resistance to a pan-TEAD inhibitor (TEADi). The study shows that gene expression is broadly restored in drug-resistant cells, accompanied by overall increased YAP binding, which however shifts more towards promoter regions. Analyses of 3D chromatin interactions at a set of 32 TEAD-target genes in TEADi-resistant cells reveals a reduction in distal enhancer (RE)–promoter contacts, despite reestablishment of YAP binding at distal enhancer elements. CRISPR-mediated deletion of several of these distal REs shows that they contribute to expression in parental cells, but are dispensable for gene activation in the resistant state. The authors then investigate FOSL1 (AP-1 component) chromatin occupancy, motivated by its previously established role in TEADi resistance (PMC11836325). They find that FOSL1 binding to chromatin is increased in both acute and resistant cells, again with a bias toward promoter regions, mirroring the YAP binding pattern. To identify potential upstream regulators of the transcriptional program in TEADi-resistant cells, the authors focus on transcription factors whose expression is either restored or increased in resistant cells or that are promoter specific, such as KLF4 and NFYA. ChIP-qPCR data shows that both factors have increased binding at a subset of TEAD-target genes in the resistant cells. Notably, the authors suggest a hierarchical regulatory network where KLF4 regulates the expression of other TEAD-target genes, including FOSL1.

In summary, this study an interesting study, elegantly presented, featuring clear writing and effective data visualization. Overall it provides compelling evidence that in acquiring TEADi resistance the cells rearrange their cis-regulation at least partially through a mechanism that is less dependent on distal enhancers and more reliant on promoter-centred regulation. A switch from enhancer- to promoter-centric gene regulation has been previously described in association with SWI/SNF-inhibition (PMC11307202). However, the current manuscript unveils such a compensatory mechanism for a cell-type specific TF family (TEAD) in a disease setting, and provides mechanistic insights into the molecular rewiring that might underlie many resistance/adaptation processes in cancer. Moreover, the application of MCC to obtain mechanistic insight into a gene regulatory process is very well don and a great example of the power of this method that has rarely been used to such good effect. Additionally, although not yet approved for clinical use, TEADi are a promising therapeutic strategy for Hippo-deficient cancers, such as mesothelioma, and are currently in clinical trials. Taking this in consideration, this manuscript offers fundamental insights and relevant contributions towards understanding resistance mechanisms to TEAD inhibitors in YAP/TEAD-driven diseases. However, given the limited set of TEAD target genes the generality of some statements should be toned down. Moreover, the model developed from the results and the presentation/analyses of some data need revision. To fully support the publication of this manuscript following major and minor comments should be addressed

Major comments

1 – YAP/TEAD co-binding and global YAP binding at promoters in resistant cells
In the model depicted in Fig. 5E, the authors present YAP and TEAD co-binding in resistant cells. Is this truly the case? The authors should have the data (YAP ChIP-seq & TEAD Cut&RUN from Paul et al 2025) to clarify the binding dynamics of YAP and TEAD in resistant cells, including whether co-binding does occur, if both factors cooperate in resistant cells and how many of the restored or novel YAP peaks are TEAD-dependent, particularly within the 32 TEAD target genes. If co-binding cannot be demonstrated, the model in Fig. 5E should be revised accordingly. Also, why is KLF4 depicted at the resistant promoter, but not YAP, FOSL1 and NFYA?
Additionally, it appears to me as if the shift towards promoter binding for YAP (as shown in Fig. 1E), is not reflected for those TEAD targets presented in Figs. 2, 3, and S2. Can the authors comment on this?
Finally, to evaluate condition-specific RE–promoter interactions involving TEAD motifs, the authors should present averaged MCC signal profiles centred on TEAD motifs across untreated, acute, and resistant states, similar to the analysis shown in Fig. 2C.

2. FOSL1 binding at promoters and integration to the proposed model

The FOSL1 promoter-specific binding shown in Fig. 4A–C is convincing, yet this is not integrated in the proposed model in Fig. 5. Furthermore, FOSL1’s functional relationship with YAP or KLF4 remains unclear, as is not shown if YAP-FOSL1-KLF4 act together to regulate the same subset of genes. In Fig. 4D, FOSL1 enrichment at TEAD-target promoters appears bimodally distributed in the resistant cells. Here, the authors should correlate FOSL1 levels at promoters with expression level of these target genes, as well (if possible) with YAP binding and RE-P interactions (e.g. What’s the MCC read count/signal distribution between promoters and distal peaks in untreated vs. resistant cells?). This could strengthen the

argument for a concerted shift towards promoter-based regulation achieved, especially at those promoters where YAP+FOSL1 bind.
Finally, I don't see the benefit of the TF-footprinting analysis as presented in Fig. 4E. The description in the results and methods lack clarity, statistical significance is not addressed, and the inconsistent symmetry across plots raises concerns about interpretability of the analysis. What is the contribution of this analysis to the overall conclusions?

3. Identification of key transcription factors that contribute to gene expression changes
While analysing TFs with increased or restored expression in resistant cells is a good starting point, a more informative approach would be to evaluate the individual contribution of each TF to gene expression changes across conditions. This could be done by integrating gene set enrichment analysis (GSEA) with transcription factor network analysis using expression data for each condition, through tools such as WebGestalt. Such analysis would help clarify the regulatory roles of the studied TFs and potentially uncover additional regulators that might warrant future investigation.

4. Generalization of the promoter switch model based on a limited selection of TEAD-target genes
The shift from distal regulatory elements to promoter-centric gene regulation is well supported for the subset of the TEAD target genes and nicely demonstrated through the CRISPR deletion experiments. However, as presented at the moment, the selection of the 32 MCC promoter targeted genes seems a bit arbitrary ("TEAD target genes that exhibit either restored or increase expression in resistant cells"). Why do the authors know these are direct TEAD targets? Why were these 32 selected vs. other genes from Clusters C1, C2 and C4 (if I am not mistaken)? The authors should give an explanation for that. Can they provide an alternative (control) set of genes that are differentially regulated (e.g. genes that do not recover from TEAD-inhibitor treatment?)
Furthermore, extrapolation from these 32 targets to a general "promoter switch" mechanism in resistant cells seems a bit premature, especially given a number of TEAD-target genes show only partial and ANKRD1 no switching
The study would be strengthened by providing bioinformatic support on why these 32 genes are representative of a general mechanism in the cells, or alternatively, why these 32 genes are well-suited to understand the mechanistic adaptation of YAP to the TEAD-inhibitor.
As of now, the 32 promoters appear to be cherry-picked without controls, and a sweeping statement as in l344/345 of the discussion ("Our work, leveraging MCC, demonstrates that most promoter elements in resistant cells exist within a dysregulated 3D chromatin configuration") is not supported.

5 - Control TEADi-resistant cells for genomic rearrangements at key loci:
From my understanding, the NCI-H226 TEADi-resistant cells were derived from the NCI-H226 cell line prior to the experimental setup with the acute and control treatments, as it is described in the methods that the resistant cell line was expanded using pooled populations of survivor cells. To confidently state that the observed changes in gene expression reflect drug resistance rather than spontaneous genomic alterations arising from longer cell culture passaging, the authors should provide basic controls such as chromosome counts or detection of copy number variations (e.g. arrayCGH) between the control cells and the resistant pool of cells, especially to rule out amplifications at key loci like KLF4 and YAP.

Minor comments:

1 - The introduction would benefit from a brief explanation of the relevance of TEAD activity in mesothelioma and the rationale for using mesothelioma cell lines in this study.
2 - The authors should avoid overgeneralizing statements in cases where the data do not provide broad support and use more precise wording throughout the study. For instance:
• Lines 163–164: "biased towards promoters in resistant cells", should be TEADi-treated cells, as a similar bias is observed in acutely treated cells.
• Line 236: "RE–P interactions" in TEAD-target genes
• Line 345: Not all promoters show this feature, only the studied TEAD-promoters.
• Lines 361–363: The wording is misleading as it suggests that the proposed mechanism occurs genome-wide, instead the data supports this only for a specific subset of genes.
3 – I suggest including a schematic model of untreated, acutely treated, and resistant cells as Fig. 1A, to illustrate the cellular model used throughout the manuscript and clarify the relation between cell conditions.
4 – If I understand correctly, the performed differential expression analysis involved a three-way comparison between untreated, acute, and resistant conditions. This should be better described in the Methods section, as we believe this is not immediately evident.
5 - To further improve already strong visual coherence across Fig. panels, the authors could consider aligning the colour scheme used in Fig. S1A and S1D with that of the other Figures.
6 - The definition criteria of "TEAD-target genes" are not provided. Please specify the criteria used to identify TEAD targets either in the Methods section or within the main Results. (see Maj. Comment)
7 – In addition to the metaplot of YAP coverage, I would like to see a heatmap of the ChIP-seq coverage at each of the 502 YAP peaks in Fig. 2B. This would help to assess how uniform the recovery of the YAP signal is across peaks (as it appears from the bigwig track in the Fig.s), and probably better show the increased signal, as the authors write.
8 - Is the "repressed common FOSL1 peaks" plot in Fig. 4A correct? It appears identical to the "repressed acute FOSL1 peaks" plot?
9 – The yaxis in FOSL1 ChIP in Fig. 4E is missing.

10 - Is NFYA upregulated in the resistant state? The manuscript presents the expression profiles of all other TFs and uses expression levels as one of the criteria for selecting TFs to investigate. To maintain consistency, the authors should add NFYA expression across all conditions.
11 - The discussion would benefit from a more detailed comparison between the mechanism proposed here and that described for SWI/SNF in PMC11307202 (lines 358–361). Expanding on both the similarities and differences would help to better contextualize the findings and clarify the novelty of the mechanism presented in this study.
12 – In addition to being a bit of an overstatement, based on the presented data (see Major Coment 3) the term “promoter switch” might not be the most accurate, as this to me implies a switch between two or more promoters.

Version 1:

Decision Letter:

Our ref: NSMB-A50953A

2nd Mar 2026

Dear Dr. Daniel,

Thank you for submitting your revised manuscript "Promoter-centric gene regulation in drug-resistant cancer" (NSMB-A50953A). Please accept my sincere apologies for the very long delay in sending you a decision, I am afraid one of the referees was unavoidably delayed. Both we and the referee convey our sincere apologies.

Your study has now been seen by the original referees and their comments are below. The reviewers find that the paper has improved in revision, and therefore we'll be happy in principle to publish it in Nature Structural & Molecular Biology, pending minor revisions to satisfy the referee #3' final requests and to comply with our editorial and formatting guidelines.

We are now performing detailed checks on your paper and will send you a checklist detailing our editorial and formatting requirements in about a week. Please do not upload the final materials and make any revisions until you receive this additional information from us.

To facilitate our work at this stage, it is important that we have a copy of the main text as a word file. If you could please send along a word version of this file as soon as possible, we would greatly appreciate it; please make sure to copy the NSMB account (cc'ed above).

Thank you again for your interest in Nature Structural & Molecular Biology Please do not hesitate to contact me if you have any questions.

Sincerely,

Carolina Perdigoto, PhD
Chief Editor
Nature Structural & Molecular Biology
orcid.org/0000-0002-5783-7106

Reviewer #1 (Remarks to the Author):

The authors have addressed all my concerns. I recommend their interesting study for publication in NSMB.

Reviewer #2 (Remarks to the Author):

The authors have added significant new data and analysis to their paper, strengthening the generality and clarity of their work. They have responded to all of the comments I raised in response to their original submission, and I recommend the revised version work for publication in its present form.

Reviewer #2 (Remarks on code availability):

As previously noted in response to the first submission.

Reviewer #3 (Remarks to the Author):

The revised version of the manuscript is greatly improved, and the additional analyses and experiments strengthen the overall conclusion. The concerns raised in my review have been sufficiently addressed. I like the term “promoter reinforcement” for the observed phenomenon as well as the new Fig. 4a-d, which provides a lot of food for thought, also from a basic gene regulatory point of view.

However, there are two minor points that I want to raise and make the authors aware of.
1. The beginning of the results section describing Figs. 1-2 (up to ~line 250) are a really tough read, certainly stemming from describing the clustering and plots. I want to encourage the authors to improve readability as you might lose readers before the really exciting and strong part of the paper (to me at least) starts with the experiments in Figure 3 and the following Figs..
2. Throughout the authors write of induced and repressed ChIP- and MCC-seq peaks, possibly trying to stay coherent with the gene expression terminology. I really struggled with making that suggestive link and I find it very inaccurate. A peak cannot be repressed. It can be gained or lost, or the signal at the peaks can be lower/weaker or higher/increased. I recommend to stay more descriptive/precise and revise this terminology.

Version 2:

Decision Letter:

29th May 2026

Dear Dr. Daniel,

We are now happy to accept your revised paper "Promoter reinforcement supports transcriptional resilience in drug-resistant cancer" for publication as a Article in Nature Structural & Molecular Biology.

Acceptance is conditional on the manuscript's not being published elsewhere and on there being no announcement of this work to the newspapers, magazines, radio or television until the publication date in Nature Structural & Molecular Biology.

Over the next few weeks, your paper will be copyedited to ensure that it conforms to Nature Structural & Molecular Biology style. Once your paper is typeset, you will receive an email with a link to choose the appropriate publishing options for your paper and our Author Services team will be in touch regarding any additional information that may be required.

After the grant of rights is completed, you will receive a link to your electronic proof via email with a request to make any corrections within 48 hours. If, when you receive your proof, you cannot meet this deadline, please inform us at rjsproduction@springernature.com immediately.

You will not receive your proofs until the publishing agreement has been received through our system.

Due to the importance of these deadlines, we ask that you please let us know now whether you will be difficult to contact over the next month. If this is the case, we ask you provide us with the contact information (email, phone and fax) of someone who will be able to check the proofs on your behalf, and who will be available to address any last-minute problems.

To assist our authors in disseminating their research to the broader community, our SharedIt initiative provides all co-authors with the ability to generate a unique shareable link that will allow anyone (with or without a subscription) to read the published article. Recipients of the link with a subscription will also be able to download and print the PDF.

As soon as your article is published, you can generate your shareable link by entering the DOI of your article here: http://authors.springernature.com/share. Corresponding authors will also receive an automated email with the shareable link

Note the policy of the journal on data deposition: http://www.nature.com/authors/policies/availability.html.

Your paper will be published online soon after we receive proof corrections and will appear in print in the next available issue. You can find out your date of online publication by contacting the production team shortly after sending your proof corrections.

You may wish to make your media relations office aware of your accepted publication, in case they consider it appropriate to organize some internal or external publicity. Once your paper has been scheduled you will receive an email confirming the publication details. This is normally 3-4 working days in advance of publication. If you need additional notice of the date and time of publication, please let the production team know when you receive the proof of your article to ensure there is sufficient time to coordinate. Further information on our embargo policies can be found here: https://www.nature.com/authors/policies/embargo.html

You can now use a single sign-on for all your accounts, view the status of all your manuscript submissions and reviews, access usage statistics for your published articles and download a record of your refereeing activity for the Nature journals.

If you have not already done so, we strongly recommend that you upload the step-by-step protocols used in this manuscript to the Protocol Exchange. Protocol Exchange is an open online resource that allows researchers to share their detailed experimental know-how. All uploaded protocols are made freely available, assigned DOIs for ease of citation and fully

searchable through nature.com. Protocols can be linked to any publications in which they are used and will be linked to from your article. You can also establish a dedicated page to collect all your lab Protocols. By uploading your Protocols to Protocol Exchange, you are enabling researchers to more readily reproduce or adapt the methodology you use, as well as increasing the visibility of your protocols and papers. Upload your Protocols at www.nature.com/protocolexchange/. Further information can be found at www.nature.com/protocolexchange/about.

An online order form for reprints of your paper is available at https://www.nature.com/reprints/author-reprints.html. Please let your coauthors and your institutions' public affairs office know that they are also welcome to order reprints by this method.

Authors may need to take specific actions to achieve compliance with funder and institutional open access mandates. If your research is supported by a funder that requires immediate open access (e.g. according to Plan S principles or the NIH public access policy) then you should select the gold OA route, and we will direct you to the compliant route where possible. Because authors warrant under our subscription licensing terms that they haven't committed to licensing any version of their article under a licence inconsistent with the terms of our agreement – including the applicable embargo period – publication under the subscription model isn't suitable for authors whose funders require no embargo.

In approximately 10 business days you will receive an email with a link to choose the appropriate publishing options for your paper and our Author Services team will be in touch regarding any additional information that may be required.

You will not receive your proofs until the publishing agreement has been received through our system.

If you have any questions about our publishing options, costs, Open Access requirements, or our legal forms, please contact ASJournals@springernature.com

Sincerely,

Carolina Perdigoto, PhD
Chief Editor
Nature Structural & Molecular Biology
orcid.org/0000-0002-5783-7106

Click here if you would like to recommend Nature Structural & Molecular Biology to your librarian:
http://www.nature.com/subscriptions/recommend.html#forms

Open Access This Peer Review File is licensed under a Creative Commons Attribution 4.0 International License, which permits use, sharing, adaptation, distribution and reproduction in any medium or format, as long as you give appropriate credit to the original author(s) and the source, provide a link to the Creative Commons license, and indicate if changes were

made.
In cases where reviewers are anonymous, credit should be given to 'Anonymous Referee' and the source.
The images or other third party material in this Peer Review File are included in the article’s Creative Commons license, unless indicated otherwise in a credit line to the material. If material is not included in the article’s Creative Commons license and your intended use is not permitted by statutory regulation or exceeds the permitted use, you will need to obtain permission directly from the copyright holder.
To view a copy of this license, visit https://creativecommons.org/licenses/by/4.0/

Manuscript #: NSMB-A50953
Title: Promoter-centric gene regulation in drug resistant cancer

Summary of key additions to the revised manuscript:

We thank the Reviewers for their thoughtful and constructive feedback, which have significantly strengthened our study. In response, we have performed new experimental and computational work, expanded the scope of our analyses, refined our mechanistic model, and toned-down some of our conclusions. Below, we summarize the major additions implemented in the revised manuscript; followed by point-by-point responses.

1. Expansion of experimental and analytical data in the H226 model.

To address requests for enhanced mechanistic understanding, we have generated and integrated a comprehensive set of new datasets in our primary cellular model, NCI-H226 (H226), including ATAC-seq, occupancy data for KFL4, p300, CTCF, TEAD1 and histone modifications H3K27ac, and H3K4me3. These datasets clarify the regulatory landscape of promoters and distal REs in this model and provide more mechanistic insight (**Fig. 4**).

We have also improved the MCC dataset by extending the probe set to include control regions whose expression is repressed in resistance vs. parental cells. Additionally, we have implemented an MCC peak calling pipeline, regulatory element (RE) annotation, and motif enrichment, enabling an in-depth investigation of promoter-RE interactions (**Fig. 2**). Using proActiv[1], we have quantified promoter activity genome-wide (**Supplementary Fig. 2c, d**) and linked these changes to looping differences and epigenomic remodeling across our treatment conditions (**Fig. 4**). We have also performed DNA FISH for *YAP1* (**Supplementary Fig. 1g**), *KLF4* (**Supplementary Fig. 5d**) and *FOSL1* (**Supplementary Fig. 4a**) to assess the contribution of copy-number variation to our observations.

2. Extension of our analyses to a second TEAD-inhibitor (TEADi)-resistant model to broaden relevance.

To address concerns regarding model specificity and the limited scope of the original submission, we have established and characterized an independent G7883-sensitive mesothelioma model, MSTO-211H (MSTO), and performed RNA-seq (**Supplementary Fig. 1a, b, c**), YAP ChIP-seq (**Supplementary Fig. 1e**), ATAC-seq (MCC peak annotation and peak calling – **Fig. 2b, c, e, f**), and MCC on 50 promoters paralleling the H226 study (**Fig. 2d-f; Supplementary Fig. 2b**).

Remarkably, the promoter-centric adaptation is preserved in this new model system, with reduced RE-P interactions at G7883-sensitive loci, and restored or increased promoter activity in resistant cells, despite model-specific differences in YAP occupancy. Using CRISPR editing, we confirm the reduced contribution of a select set of REs to the observed gene activation in resistant MSTO cells (**Fig. 3d, e, f**). This new model strengthens our previous findings and demonstrates that the phenomenon extends beyond a single system.

3. Integrated promoter-RE linkage analysis supports a refined mechanistic model.

Peak-resolved MCC maps allowed us to define promoter-RE linkages and integrate these with epigenomic features (**Fig. 4; Supplementary Fig. 6**). In H226, genes with recovered expression in resistant cells consistently show:

- weakened promoter-RE interactions
- restored H3K4me3 at promoters and restored promoter activity,
- loss of p300 and H3K27ac at both promoters and distal REs, and
- resistance-induced KLF4 binding at promoters and at dysfunctional distal REs

These integrated data support a refined model in which the studied promoters engage a compensatory regulatory response - termed *promoter reinforcement* - when distal regulatory element function is impaired. Collectively, the extensive new experiments and analyses substantially strengthen the rigor, mechanistic depth, and generalizability of the study. We hope that the revised manuscript is now suitable for further consideration at ***Nature Structural & Molecular Biology***.

Point-by-point responses to Reviewer comments:

Reviewer #1 (Remarks to the Author):

In this manuscript, Kameswaran and colleagues investigate how 3D cis-regulatory landscapes of cancer cells (mesothelioma cancer cells; NCI-H226) change in response to a drug targeting the transcription factor TEAD. They compare both an acute and prolonged drug treatment in which the cells have become resistant to the drug. Interestingly, they discover that interactions between gene promoters and distal regulatory elements remain reduced in resistant cells despite recovery of gene expression levels. Editing of these elements shows that the resistant cells have become independent of these elements. Based on analyses of transcription factor binding and promoter activity, the authors propose that these genes have become more dependent on regulation via their promoter and that this “promoter-switch” may be a common mechanism for induced resistance to drugs targeting transcription factors in cancer cells.

This manuscript is clear and well-written and presents very interesting data that contribute to our mechanistic understanding of how the regulatory landscapes of cancer cells adapt to drug treatments. Although it remains unclear how generalizable these findings are, I find these findings in principle sufficiently interesting to justify publication in NSMB. However, at the moment, the conclusions are in my view not yet fully supported by the data. I have the following suggestions for the authors to improve their manuscript:

We thank the Reviewer for their positive assessment of our work and constructive suggestions. We have performed additional experiments and analysis to address each of the Reviewer suggestions, as detailed below.

Major comments

1. I find it curious that YAP binding levels at distal elements seems to increase in resistant cells, even though these elements become less functional. Can the authors comment on this observation?

We agree that this is a curious observation. In H226 cells, we previously showed that YAP binding is restored or increased at distal regulatory elements (REs) in resistant cells despite weakened RE-promoter interactions (**Fig. 2b**), and that deletion of these distal REs predominantly affects gene expression in parental cells, indicating reduced distal RE dependence in resistance (**Fig. 3a, b; Supplementary Fig. 3a, b**).

Our expanded datasets refine this interpretation and clarify the regulatory state of distal REs and promoters in resistance. Chromatin accessibility increases broadly in resistant cells, with substantially more ATAC peaks opening (16,212) than closing (5,737), and opening regions are enriched at promoters (26% vs. 9% for closing sites), indicating promoter-biased chromatin remodeling (**Supplementary Fig. 7a, b**). At globally opening regions, p300 binding is reduced upon acute G7883 treatment and largely restored in resistance, whereas H3K27ac remains low (**Supplementary Fig. 7a, b**). Accessibility and YAP/TEAD1 occupancy also increase at MCC-linked RE-promoter pairs (**Supplementary Fig. 6b**).

Notably, while promoters uniquely regain H3K4me3 and transcriptional activity[1], neither p300 nor H3K27ac fully recover at distal REs or promoters (**Fig. 4a-d**). Thus, resistant cells re-establish YAP/TEAD1 binding without restoring distal RE H3K27ac or full enhancer function[2,3], whereas promoter activity may persist through H3K4me3-dependent mechanisms as described previously[4,5]. This can explain why MCC-linked distal REs remain functionally compromised despite increased accessibility and restored/gained YAP/TEAD1 occupancy, supporting a shift toward a promoter-centered transcriptional state in which distal REs become accessible but uncoupled from gene activation[6,7].

Importantly, analysis of a second TEADi-resistant model, MSTO-211H (MSTO), reveals similar core features - reduced RE-promoter interactions and restored or gained promoter activity in resistance - despite the absence of restored YAP binding at distal REs (**Fig. 2e**). This indicates that while distal RE occupancy patterns may vary across models, reduced distal RE reliance and promoter-centered adaptation are shared properties of the resistant state.

2. The MCC data are poorly annotated. It has been shown in the original MCC paper (Hua et al. Nature 2021) that promoters frequently interact with both enhancers, CTCF-binding sites, and other promoters. In this manuscript, it is implied that all MCC peaks represent enhancer-promoter interactions, but this is difficult to judge from the data. It would be really helpful if the authors would also show CTCF tracks – even if only for the parental cell line. A better classification and quantification (see below) of the MCC interactions might help the authors to better interpret why not all loci respond in the same way to treatment/resistance.

We thank the Reviewer for this suggestion. To address it, we first developed a new MCC peak-calling workflow (**described in Methods, Page 35-36, Lines 1056-1078**) that identifies promoter-linked MCC peaks using local-background/MAD (Median Absolute Deviation) thresholding and filters them by ATAC-seq accessibility to ensure biological specificity. MAD provides a robust, outlier-insensitive estimate of local background variation, enabling reliable peak detection in sparser MCC profiles. This approach yielded a defined set of MCC interactions/peaks

(H226: 543 for 40 promoters; MSTO: 1,838 for 50 promoters) that can be consistently annotated and compared across conditions **(Fig. 2b, e)**.

Using these peak-called MCC interactions, we next performed comprehensive annotation with CTCF and YAP occupancy data in both H226 (**Fig. 2c, see below**) and MSTO parental cells (**Fig. 2c, f, see below**). This analysis shows that many MCC-linked regions correspond to YAP and/or CTCF-bound REs, while others fall within promoters (defined as MCC peaks intersecting ±2 kb of GENCODE TSSs) or other ATAC-seq defined accessible regions (Other).

Figure 2.

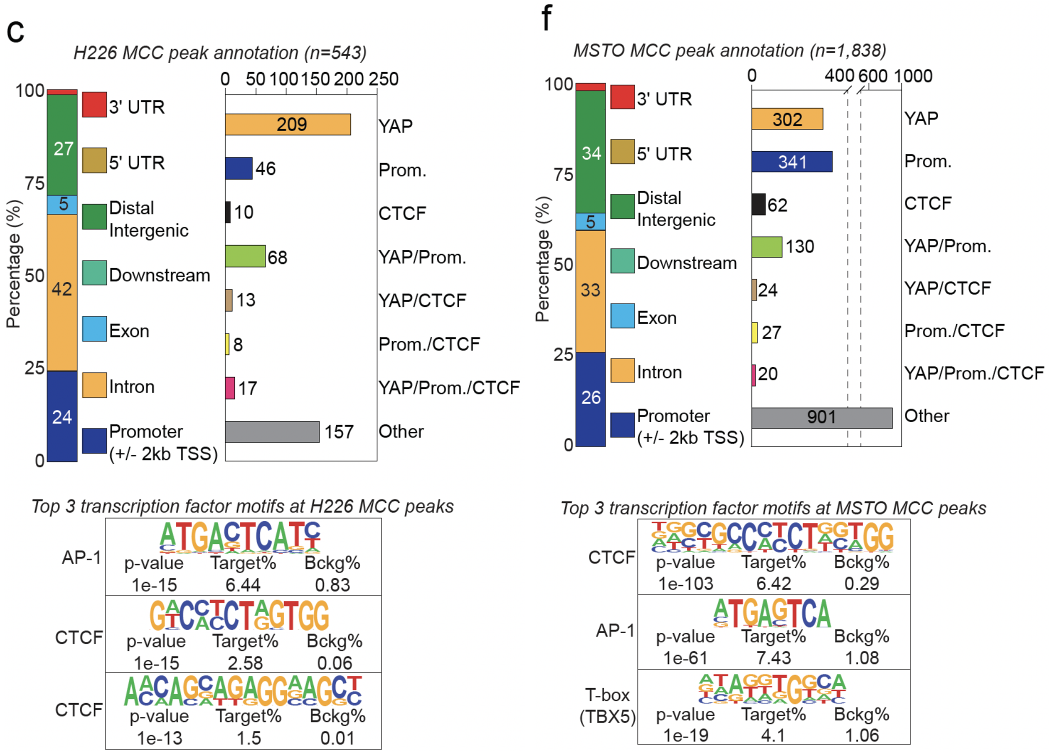

<u>Figure legend:</u> Annotation of H226 **(Fig. 2c)** and MSTO **(Fig. 2f)** MCC peaks to genomic features and their overlap with YAP, CTCF, promoter sites, and the combinations of these. The "Other" group represents MCC peaks that overlap with open chromatin regions from ATAC-seq (top). *De novo* motif enrichment at MCC peaks in MSTO cells (bottom).

Importantly, inclusion of CTCF tracks revealed that at several of our example gene loci, promoter-CTCF interactions weaken or are lost in resistance. We observe such cases in both models - for example, the -27kb CTCF-bound RE at the *TACC3* locus in H226 (**Fig. 3b**), and the -214kb/-212kb CTCF-bound REs at the *RGS4* locus in MSTO (**Fig. 3f**), all of which exhibit weaker interactions with promoters in the resistant state, and CRISPR experiments prove their diminished function. These findings emphasize that loss of RE function in resistance is not limited to YAP-bound regions but also applies to CTCF-bound REs, supporting a broader disruption in promoter-RE connectivity and function.

Together, the updated peak-calling and annotation pipeline clarifies the promoter-RE interaction landscape and provides additional mechanistic insights into resistance remodeled 3D chromatin interactions.

3. The quantification of the MCC data is not very thorough. The authors only show enrichment at AP-1 motifs in Figure 2C. This seems a bit arbitrary. An advantage of the MCC data is that they can be peak-called. The peaks can then be classified in different categories and easily quantified. This strategy seems more robust and has been used in previous publications with MCC data (scripts should be available, e.g. in Hua et al. Nature 2021 or Karpinska et al. NSMB 2025). It should be noted that the quality of the MCC data in this manuscript seems substantially lower than in previous publications, so it would be important that the authors carefully tune their peal call / quantification strategy.

We appreciate this suggestion, in the revised version, we now quantify MCC signals at the peak-called sites (described in **Methods, Page 35-36, Lines 1056-1078**). For both H226 and MSTO, we display heatmaps and histograms of MCC read distributions across all peaks (**Fig. 2b, e - see below**) and provide direct statistical comparisons of MCC signal between conditions (Wilcoxon signed-rank test, p<0.01; **Supplementary Fig. 2b**). These analyses offer a more rigorous and comprehensive quantification of MCC interactions (**Pages: 8-9, Lines: 209 - 237**).

Figure 2 and Supplementary Figure 2.

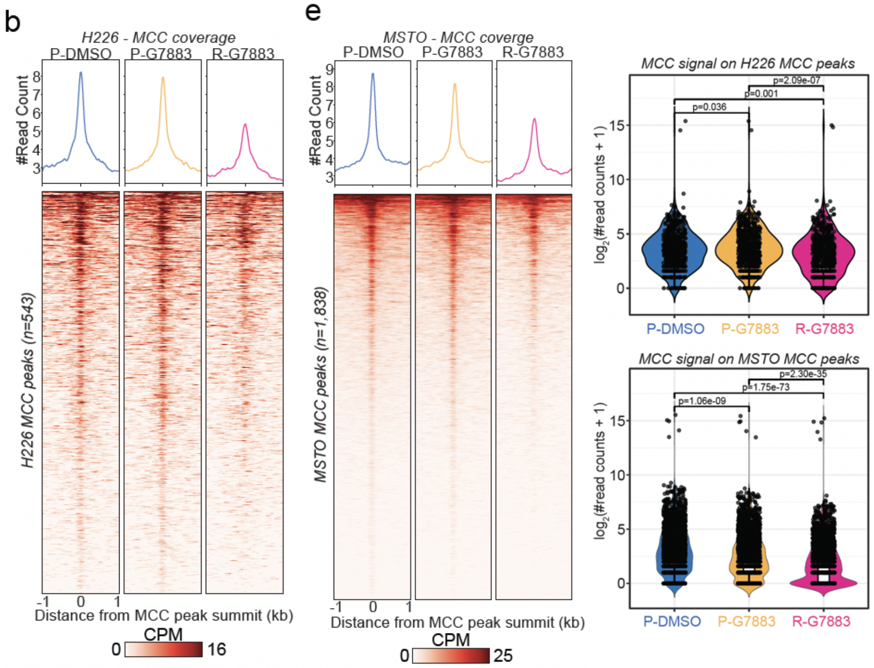

<u>**Figure legend:**</u> Aggregated read-density plots and corresponding heatmaps depicting normalized MCC contact profiles in H226 (**Fig. 2b**) and MSTO (**Fig. 2e**) conditions at peak called genomic regions. Violin plot quantification of normalized (# - CPM) MCC read count in the two cell line models across conditions (**Supplementary Fig. 2b**).

4. A strong part of the paper is that the authors perform genome editing to perturb the distal elements to demonstrate that the selected loci are no longer dependent on these elements for their expression in the resistant cells. However, since the authors propose that the independence of the elements is a mechanism of resistance, it would be very interesting to know if/how the “acutely” treated cells (P-G7883) depend on these elements.

We thank the Reviewer for this suggestion and agree that inclusion of the acute treatment condition would strengthen the interpretation of distal RE dependence. We focused on one of our canonical examples, *FOSL1*, and repeated the promoter and -15kb RE deletions in DMSO and G7883 acutely treated (48-hours) parental H226 and MSTO cells. As expected, *FOSL1*

expression is already markedly reduced in the acute inhibitor treated state relative to DMSO controls, limiting the dynamic range for further repression. Consistent with this, promoter deletion does not further affect *FOSL1* expression in either model. In contrast, deletion of the -15kb RE produces an additional decrease in *FOSL1* expression in acutely stimulated H226 cells, whereas the same perturbation has no additional effect on already repressed expression in MSTO cells. These results (**Supplementary Fig. 3d - see below**) suggest that acute TEAD inhibition can reduce transcription almost as strongly as RE removal in the *FOSL1* locus, and suggest that in this state, further dependence on this RE is marginal. We describe these results on **Page: 10, Lines: 289-293**.

Supplementary Fig. 3d

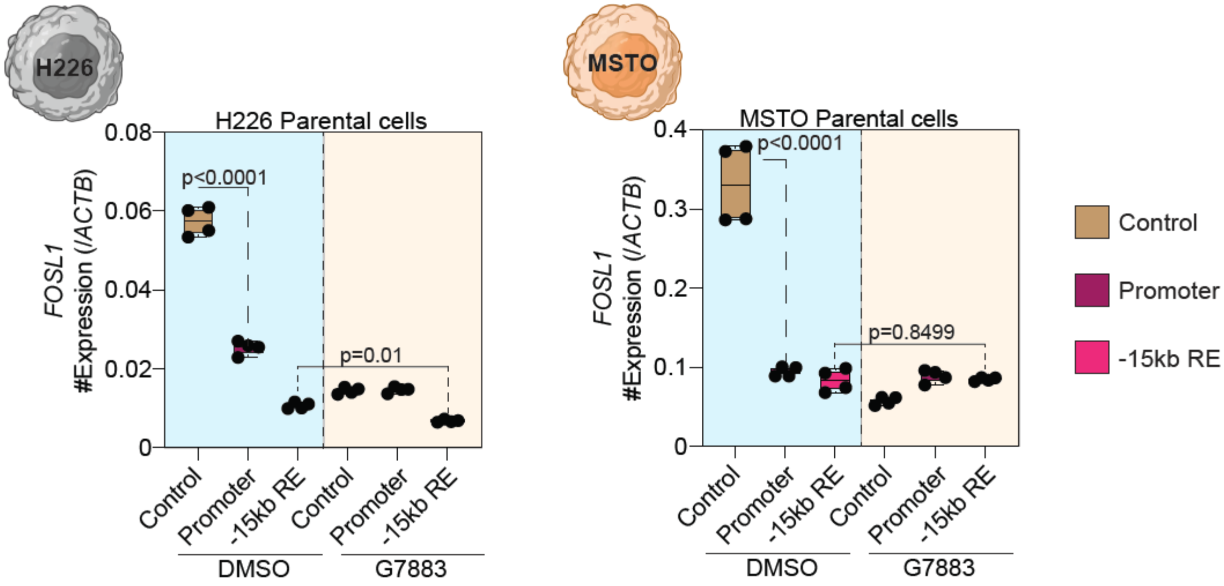

Figure legend: CRISPR-mediated perturbation of the promoter and a distal RE upstream of *FOSL1* in parental and acutely GNE-7883-treated H226 and MSTO cells. Boxplots show relative FOSL1 expression measured by RT-qPCR under the indicated conditions, with the same boxplot structure and sample size as above. Statistical significance was assessed using two-tailed unpaired *t*-tests ($p<0.05$).

5. The FOSL1 binding distribution in resistant cells in Figure 4D seems different compared to the other conditions (bimodal?). Does this relate to some of the observed differences in the loci of interest? E.g., do the genes that lose most distal MCC interactions, gain most FOSL1 at the promoter and/or vice versa?

We thank the Reviewer for this observation. In the revised manuscript, we further analyzed FOSL1 binding and find that the bimodal distribution in resistant cells reflects promoter-level heterogeneity rather than a direct relationship with the extent of distal MCC interaction loss. At the genome-wide level, resistance-induced FOSL1 binding becomes more promoter-biased (**Supplementary Fig. 4b-d**). However, when focusing specifically on MCC-targeted promoters (n

= 40, including promoters of genes without transcriptional recovery), average FOSL1 occupancy changes are modest and variable across loci (**Fig. 4c, e**).

Importantly, genes exhibiting the strongest reduction in RE-promoter interactions do not show correspondingly higher FOSL1 binding at promoters (**Fig. 4c, d**). Thus, variation in FOSL1 occupancy does not explain differences in looping loss across MCC target genes. Instead, our integrated multi-omic analysis shows that resistance-associated transcriptional recovery correlates most consistently with increased KLF4 promoter binding and restored promoter H3K4me3, together with reduced p300 and H3K27ac at promoters and distal REs (**Fig. 4c-f**; **Supplementary Fig. 6 b-d**).

Accordingly, while FOSL1 shows a global shift toward promoter-biased binding in resistance, promoter-associated KLF4 enrichment and H3K4me3 recovery - not FOSL1 binding - best explain gene expression restoration at MCC target loci. These findings are described in the revised manuscript (**Pages 12-13, Lines 351-385**).

Figure 4. - see below

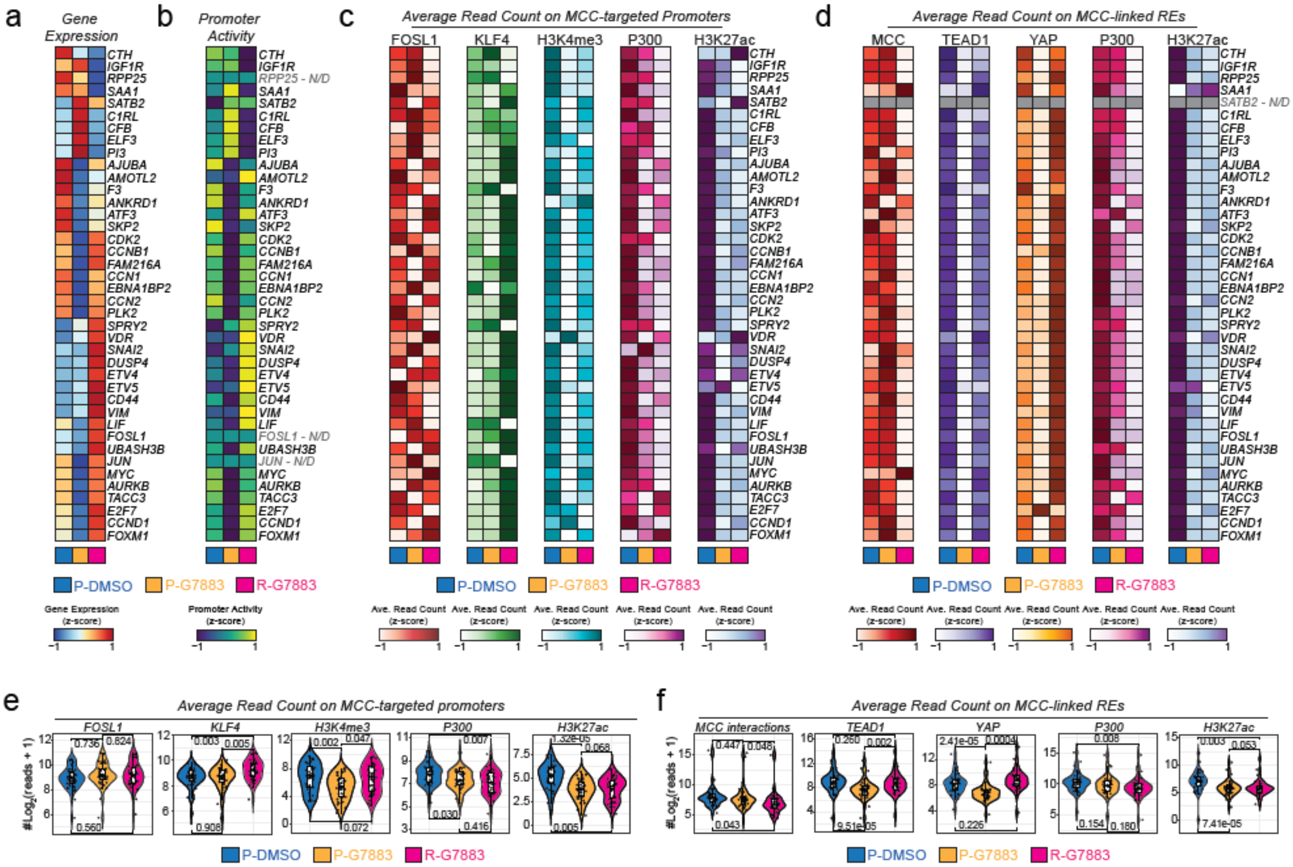

Figure legend: (**Fig. 4a, b**) RNA-seq expression and promoter activity (z-scores) for MCC targets (n = 40) across P-DMSO, P-G7883, and R-G7883. (**Fig. 4c, d**) Average normalized read counts (z-scores) at MCC-targeted promoters and MCC-linked distal REs for indicated TFs and histone marks. (**Fig. 4e, f**) Violin plots of normalized (# - CPM) read count distributions at promoters and distal REs (Wilcoxon test, $p < 0.05$). N/D - not detected.

6. Since the authors propose that KLF4 has a key role in coordinating the promoter-driven gene expression in resistant cells, it would be helpful if the authors would assess this genome-wide via ChIP-seq analysis rather than ChIP-qPCR at a handful of selected genes.

We concur with the reviewer's point and therefore performed genome-wide KLF4 ChIP-seq in the H226 model and integrated these results into our multi-omic analyses at MCC-linked RE-P interactions as described above (**Fig. 4c, e and Supplementary Fig. 6b, c**). Globally, we identify 1,346 KLF4 peaks that are reduced and 1,149 KLF4 peaks that are induced in the resistant state (FDR<0.01; **Supplementary Fig. 5e - see below**). Importantly, resistance-induced KLF4 peaks show a strong positional shift toward promoters: 21% of induced peaks map to promoter regions (+/-2 kb TSS) compared to only 7% of resistance-repressed peaks, whereas repressed peaks are more frequently gene distal (**Supplementary Fig. 5e, right panels**). Thus, while some sites lose KLF4 binding, resistance is characterized by a selective expansion of promoter-localized KLF4 occupancy.

These genome-wide data, together with the promoter-level analysis at MCC-target genes (**Fig. 4c, e**) and the KLF4 knockdown experiments (**Fig. 5b-d**), support our conclusion that KLF4 plays a central, promoter-biased role in coordinating gene expression of our MCC-target genes in the resistant state. In the revised version, we describe these findings on **Page 12, Lines 335-347**.

Supplementary Fig. 5e

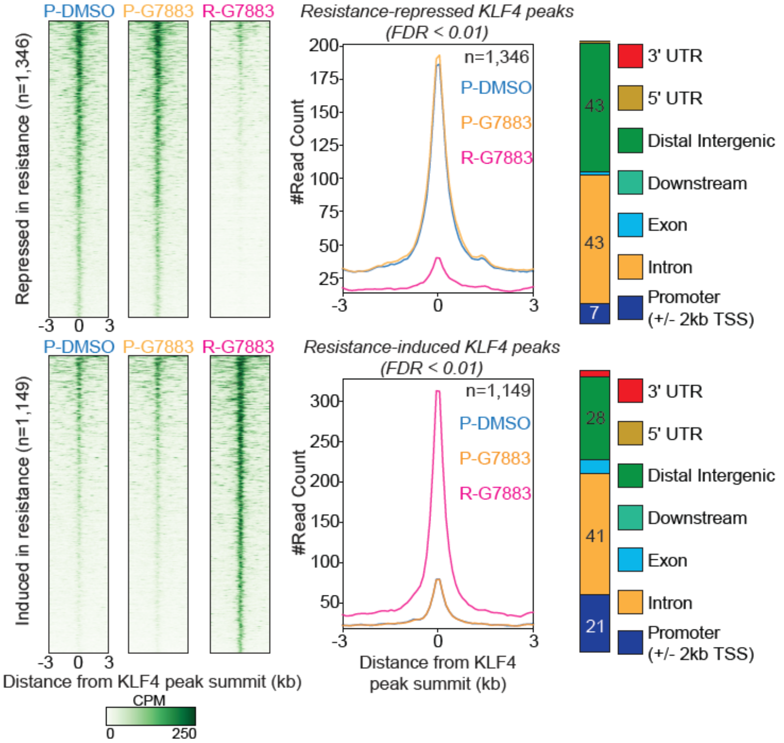

Figure legend: Differential KLF4 ChIP–seq binding analysis in resistant versus parental cells (FDR<0.01). Heatmaps display normalized read density at KLF4 binding sites that are significantly repressed (top) or induced (bottom) in resistance. Histograms (right panels) show corresponding average KLF4 signal profiles centered on peak summits. The stacked bar plot illustrates the genomic annotation of repressed and induced KLF4 peaks.

7. It would also be helpful if the authors could include the FOS1 and KLF4 ChIP-seq data directly under the MCC data in the example panels so the readers can more easily/comprehensively interpret all described regulatory changes at loci of interest.

We thank the Reviewer for this helpful suggestion. In the revised manuscript, we have expanded our genome browser panels to include the full set of relevant ChIP-seq/CUT&RUN datasets directly alongside the MCC signal at each example locus. Specifically, we now display FOSL1, KLF4, YAP, p300, H3K4me3, H3K27ac (all conditions), and CTCF (only from parental cells) profiles together with MCC interactions (**Fig. 4g; Supplementary Fig. 6e**).
We have also included CTCF ChIP-seq and CTCF CUT&Tag[8] tracks for both H226 and MSTO parental cells, respectively; and present these tracks in all CRISPR-editing examples (**Fig. 3a-f; Supplementary Fig. 3a-c**).

Together, these expanded genome browser panels provide a more comprehensive view of the regulatory changes at each locus and allow readers to readily interpret how transcription factor binding, histone modifications, and chromatin architecture change across conditions.

8. Related to comment 3: the MCC data are not very deep / a bit noisy. The quality generally seems sufficient for the analyses of the authors, but the MCC footprinting is not very convincing in my opinion and I think it would be better if the authors would de-emphasize these analyses.
We thank the Reviewer for this comment. In line with this suggestion, we have removed the TF-footprinting analysis from the manuscript.

Minor comments
1. Regarding the first sentence of the abstract: not all eukaryotic cells contain distal regulatory elements (e.g., yeast does not). Regarding the first sentence of the introduction, promoters should not be included in the listed "long-range regulatory elements".
We thank the Reviewer for these important suggestions. We have revised the text to refer specifically to mammalian cells instead of eukaryotic cells (**Page 2, Line 33**) and have removed promoters from the list of long-range regulatory elements in the introduction **Page 3, Lines: 50-52**).

2. It would be helpful if the R-G7883 cells were better described in the main text. Since these cells are crucial to understand the main message of the paper, it would be nice if readers do not have to read another paper to understand the nature of these cells and how they were generated.
We agree and now included a schema in **Fig. 1a** to clarify how the different cell states are defined. We have also included a short description in both the results and methods sections to describe the generation of these cell lines (**Results: Page 5, Lines 112-114; Methods: Page 26, Lines: 776-783**).

3. Line188: The interactions in the ANKRD1 locus are not "fully" restored.
We agree and we have revised this section (**Page 10, Lines: 282-288**).

Reviewer #2 (Remarks to the Author):

This manuscript by Kameswaran and colleagues investigates characteristics of a mesothelioma cell line with induced resistance to GNE-7883, a pan-TEAD inhibitor. The primary observation is that when mesothelioma cells develop resistance to GNE-7883 they have reduced activity of some enhancer sites enriched for binding of proteins relevant to the pathway that is being inhibited. A team that includes authors of the present study have previously published that GNE-7883 treatment remodels chromatin in OVCAR-8 cells, closing it at distal regions that are enriched for TEAD motifs and opening it at promoters of upregulated genes, with most significant transcriptional effects on YAP/TAZ targets (Hagenbeek et al 2023). This work extends that observation through additional epigenomic assays and functional studies demonstrating the reduced role of specific enhancer sites.

Strengths of the study include the use of a newly developed chemical agent that impacts activity of a clinically important pathway, and the use of the MCC assay to evaluate promoter-enhancer contacts. The experimental data are presented clearly. However, there are several weaknesses in the present study that preclude my recommending it for publication in its present form.
We thank the Reviewer for the constructive evaluation of our manuscript. Below, we have provided a point-by-point response to all comments.

1) Tumor progression and adaptation to therapy is often accompanied by reorganization of enhancer activity (e.g. Roe Cell 2017, Liu JCI 2021). Are the investigators seeing a loss of enhancer activity because they are specifically focusing on the targets of GNE-7883 in cells that have adapted to TEADi, or is this a general phenomenon affecting enhancers of many other genes? It would be logical to perform ATAC-seq and ChIP for H3K27ac marks in the three cell states to demonstrate that elimination of enhancer-promoter contacts are linked to general changes in chromatin conformation.

We thank the Reviewer for raising this important point. To determine whether reduced enhancer activity is locus-specific or global, we examined H3K27ac and p300 levels at opening and closing chromatin regions defined by ATAC-seq across all three H226 conditions. Globally, resistance is characterized by extensive chromatin opening (16,212 ATAC peaks gained vs. 5,737 lost; FDR<0.01, $Log_2FC>0.5$). At these opening regions, p300 is repressed by acute inhibitor treatment but its binding is restored in resistance, whereas H3K27ac shows little or no recovery, indicating that resistant cells gain accessibility but fail to re-establish active enhancer-associated H3K27 acetylation, suggesting impaired histone acetyltransferase (HAT) function (**Supplementary Fig. 7a, b - see below on the next page**). In the revised version, these results are described in a separate section, entitled: *Enhanced chromatin opening despite impaired HAT activity in resistance* - on **Page 14, Lines: 387-408**.

Supplementary Figure 7.

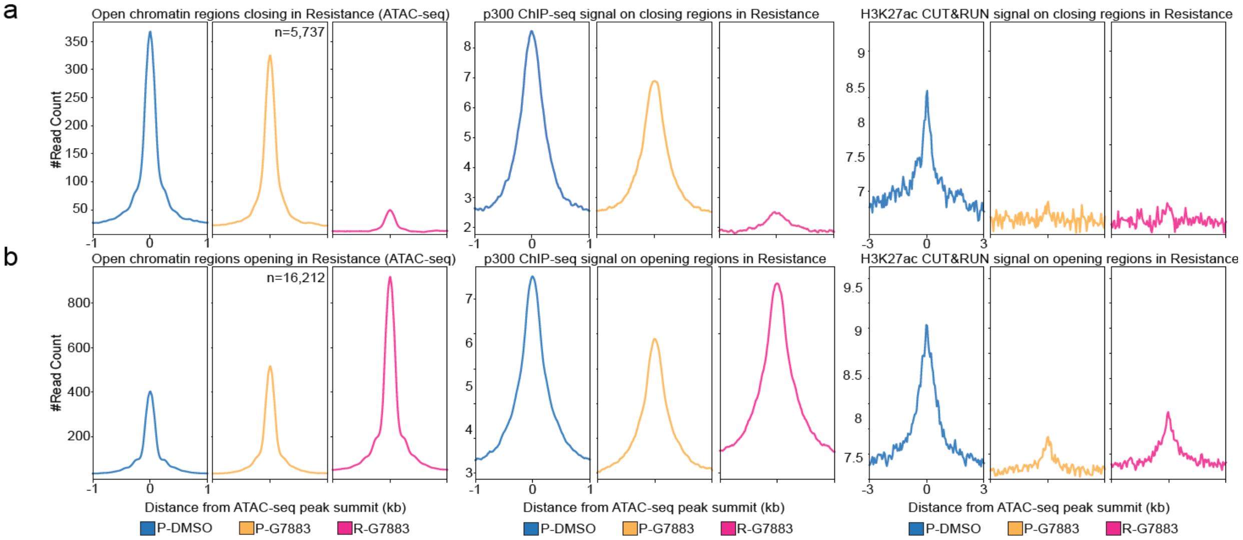

Figure legend: **(a)** Aggregate ATAC-seq profiles at regions repressed in resistant cells, with corresponding p300 ChIP-seq and H3K27ac CUT&RUN signals across P-DMSO, P-G7883, and R-G7883 conditions. **(b)** Aggregate ATAC-seq profiles at regions induced in resistant cells, with matched p300 and H3K27ac profiles across the same conditions.

In contrast, in our targeted analysis of MCC-linked RE-P pairs that we established by peak calling in the revised manuscript (**Methods, Page 35-36, Lines 1056-1078)**, on average, we observe no meaningful recovery of either p300 or H3K27ac, despite restored YAP/TEAD1 occupancy and increased accessibility in resistance (**Fig. 4c-f – see below on the next page; Supplementary Fig. 6b-d**). However, at promoters of MCC-target genes with restored or gained expression and promoter activity[1], we also find a recovery of the active promoter mark H3K4me3[9] in resistance (**Fig. 4c, Supplementary Fig. 6d**). These H3K4me3 gains along with KLF4 binding together represent the most prominent epigenetic features associating with genes/promoters that regain expression/activity in resistance and further reinforce the shift toward promoter-centric gene regulation when distal RE-level acetylation and connectivity is compromised (**Fig. 4a-d – see below on the next page**).

Figure 4.

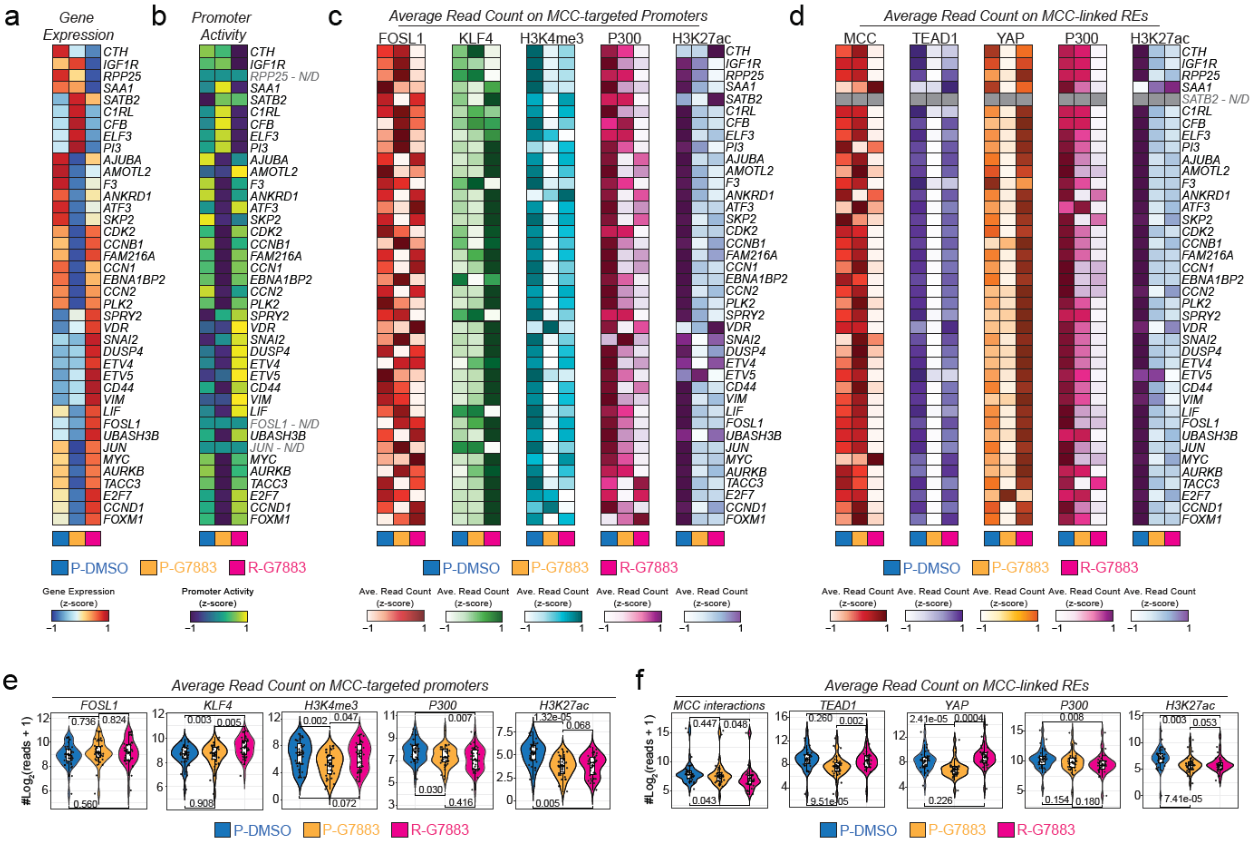

Figure legend: (**Fig. 4a, b**) RNA-seq expression and promoter activity (z-scores) for MCC targets (n = 40) across P-DMSO, P-G7883, and R-G7883. (**Fig. 4c, d**) Average normalized read counts (z-scores) at MCC-targeted promoters and MCC-linked distal REs for indicated TFs and histone marks. (**Fig. 4e, f**) Violin plots of normalized (# - CPM) read count distributions at promoters and distal REs (Wilcoxon test, $p < 0.05$). N/D - not detected.

Collectively, the results suggest a more widespread dysfunction across REs in the resistant state that is more pronounced at TEADi-sensitive RE-P pairs assayed by MCC. However, the studied promoters can regain H3K4me3 levels, which correlates with transcriptional recovery despite diminished enhancer function.

1b) For unbiased assessment of promoter activity, it would be appropriate for the investigators to use computational analysis of RNA-seq data contrasting the three main conditions (e.g. using methods published in PMID 31491388) to evaluate whether differential promoter activity is enriched at genes where enhancer-promoter contacts have been measured to be different.

We thank the Reviewer for this important suggestion. We have implemented the suggested unbiased promoter activity[1] analysis in the revised manuscript. Comparing parental and resistant states, we identified 578 resistance-induced and 794 resistance-repressed promoters in H226, and 632 induced and 376 repressed promoters in our new cell line model, MSTO-211H (LFSR < 0.1; **Supplementary Fig. 2c**).

We then examined our MCC target promoters, focusing on genes with restored or gained expression in resistance (n=32 in H226 and n=30 in MSTO), and found that promoter activity changes closely parallel gene expression changes across the three conditions (**Fig. 4a, b**). Importantly, a substantial fraction of these genes also show significant corresponding gains in promoter activity in resistance compared to parental cells (13 of 32 in H226; 10 of 30 in MSTO). Most of the remaining loci show partial or full restoration to parental levels after acute G7883 treatment-induced repression. These promoter-level changes provide an independent and quantitative readout that captures the major expression trends observed in resistance. In the revised version, we present these results on **Supplementary Fig. 2d**, while we describe these results on **Page: 9, Lines: 238-248.**

Finally, we have incorporated these promoter activity profiles into our new integrative multi-omic analysis (**Fig. 4a-f - shown in response to the comment above**). Together, these results demonstrate that promoter activity generally mirrors gene expression dynamics and associates with H3K4me3 levels at promoters, even when RE-P interactions weaken, and thereby further supports promoter-centric mechanisms as key features of transcriptional recovery in resistance.

2) The authors suggest they have identified a general principle of the elimination of enhancer site activity during therapy resistance from a single targeted drug evaluated in a single cell line. It would be important to show that the proposed adaptation of promoter-centric response as a therapy resistance mechanism is not a specific feature of NCI-H226 and GNE-7883 cells by demonstrating that these results can be observed in additional cell line models.

We recognize the importance of this suggestion. To evaluate whether promoter-centric adaptation is unique to the H226 model, we extended our analyses into an additional pleural mesothelioma model, MSTO-211H (referred to as MSTO), which carries an inactivating *LATS2* mutation and as a result displays high YAP/TAZ/TEAD activity. MSTO cells are sensitive to G7883 but acquire a resistant state in which they continue proliferating despite TEAD inhibition[10].

Using a newly designed MCC probe panel (n = 50), combined with our new peak-calling strategy (**Methods, Page 35-36, Lines 1056-1078; Fig. 2d-f**), we demonstrate that MSTO-resistant cells recapitulate the major regulatory features observed in H226. This includes reduced distal RE-promoter interactions (**Fig. 2a, b, d, e; Supplementary Fig. 2b**), diminished enhancer contribution (*RGS4* locus), and reliance on promoter-driven transcription in resistance, as confirmed by CRISPR perturbations at *FOSL1* and *TACC3* loci (**Fig. 3d-f**).

Notably, MSTO cells do not show restoration of YAP binding at MCC-linked REs following acute inhibitor treatment, in contrast to H226 cells where YAP occupancy is restored or increased in resistance (**Fig. 2 b, e – see below on the next page**). Thus, while the molecular details of YAP occupancy differ between models, the emergence of promoter-centered transcriptional regulation under TEAD inhibition is shared on our example genes. These findings are described in the revised manuscript on **Pages 8-11**.

Figure 2.

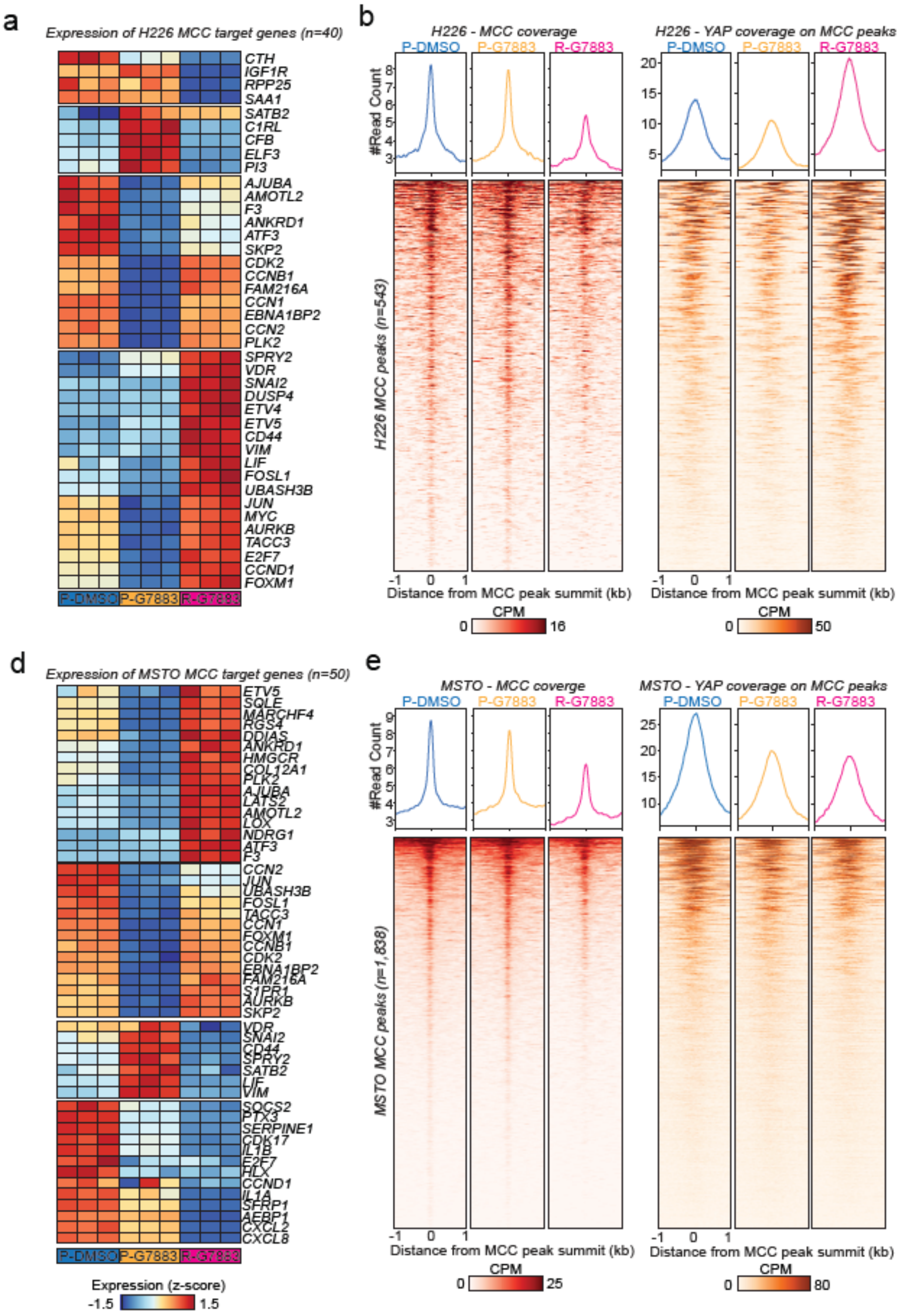

Figure legend: Heatmaps represents expression (z-score) of genes selected for MCC profiling in H226 (n=40) (**Fig. 2a**) and MSTO cells (n=50) (**Fig. 2d**) across parental (P-DMSO), acutely TEAD-inhibited (P-G7883), and resistant (R-G7883) conditions. Aggregated read-density plots and corresponding heatmaps depicting normalized MCC contact profiles (left) and YAP ChIP-seq coverage (right) centered on MCC peak summits in H226 (**Fig. 2b**, n=543) and in MSTO (**Fig. 2e**, n=1,838). # -indicates normalized CPM

3) The causal inferences drawn from the study are in some cases not justified by the observations. For example: "Our study introduces the idea that promoters can serve as central regulators of gene expression in drug resistant cancer cells"; this statement implies that this study has discovered that regulation at gene promoters is the key determinant of transcriptional activity. The statement "most promoter elements in resistant cells exist within a dysregulated 3D chromatin configuration compared to parental cells, where REs and promoters are uncoupled in the 3D space" is hard to justify given the targeted nature of the chromatin conformation analysis. The statement "in the resistant state, multiple genes' expression was more sensitive to perturbation of the promoter-specific TF, NFYA (e.g., ANKRD1, CCN2, ETV4, FOSL1, VIM), suggesting that promoter-driven gene expression is favored in resistant cells" makes a general assertion about the role of promoter-driven gene expression at all genes, when only a handful of genes are evaluated using targeted assays.

We agree with the Reviewer that several statements in the original version could be interpreted as over-generalizations relative to the targeted nature of our assays. In the revised manuscript, we have toned down these conclusions and now explicitly limit our claims to the set of genes and RE-P interactions examined. We no longer state that promoters act as "central regulators" in drug-resistant cancer cells in general, nor do we imply that most promoters, genome-wide, exist in a dysregulated 3D configuration. Additionally, we carefully edited the **Discussion**, pointing to the targeted nature of our study and the major limitation of generalizability **– Page 16, Lines: 471-477 -** "*In summary, our findings reveal a shift in gene regulatory logic in TEADi-resistant mesothelioma cells, in which promoter-centered regulation can support transcriptional resilience under therapeutic pressure. While this study focuses on a defined set of TEAD-responsive loci, it raises the possibility that promoter reinforcement contributes more broadly to adaptive gene regulatory responses in cancer. Determining the generality and therapeutic implications of this mechanism across additional drug-resistant cancer models will be an important direction for future work.*"

Regarding NFYA, we have removed these datasets from the revised manuscript since NFYA is not resistance-induced, and we felt that including them distracted from the major results we obtained for KLF4, which is the primary transcriptional regulator in this system based on our current datasets.

Although we have greatly extended our datasets and added a new cell line model - we overall clearly emphasize in the revised text that our findings illustrate examples of promoter-centered regulation observed at a select set of genes, rather than establishing a broad causal mechanism across all genes in a genome-wide manner.

4) It is unclear whether the observed differences in enhancer usage at specific sites are important for the development of resistance to TEADi therapy. The most straightforward interpretation is the one already present in Hagenbeek et al; that these enhancer sites are important for normal functioning of the tumor cells and contain binding sites that are disfavored when cells have adapted to TEADi treatment.

We agree with the Reviewer that we cannot conclusively link changes at multiple enhancer sites to the development of resistance. We have now toned down the conclusions in the revised manuscript such that our interpretation now focuses only on the loci we examined directly.

That said, we note that our KLF4 perturbation experiments provide a clear example of a TF with promoter-biased genomic localization (KLF4 ChIP-seq - **Supplementary Fig. 5e**), that can support transcriptional recovery in resistance. KLF4 occupancy at promoters is accompanied by restored promoter activity, gene expression, H3K4me3 levels (**Fig. 4a-c**), and *KLF4* knockdown reduces the proliferative capacity of resistant H226 cells (**Fig. 5d**). Nonetheless, we present this as an illustrative example rather than the exact mechanism of resistance.

5) I don't understand the scale being used for mRNA expression levels in figure 3.
We thank the Reviewer for pointing out this limitation of the figure legend. We have revised the figure panels to better indicate on the y-axis that the plot depicts normalized mRNA expression level relative to the expression of the housekeeping gene, *ACTB* (**Figure 3**). Additionally, we edited the figure legend and describe in the methods section as well how normalization was performed in our RT-qPCR assays: **Methods: Page 27, Line 806** - **"***Transcript levels (mRNA) were normalized to ACTB expression.*"; **Figure 3 legend: Page 19, Lines: 553-555** - "*Boxplots represent ACTB-normalized (#) mRNA levels of the genes in the indicated conditions following CRISPR deletion.*".

Reviewer #2 (Remarks on code availability):

I made a quick review of the MCCTools github site. The documentation appears to be professional and mature, with hallmarks of an experienced developer. I did not attempt to download or run the code.
We appreciate the reviewer's assessment of the MCCTools documentation.

Reviewer #3 (Remarks to the Author):

In the manuscript "Promoter-centric gene regulation in drug-resistant cancer", Vasumathi Kameswaran and colleagues investigate the mechanisms by which gene regulation is reconfigured in mesothelioma cancer cell lines that were developed to acquire resistance to a pan-TEAD inhibitor (TEADi). The study shows that gene expression is broadly restored in drug-resistant cells, accompanied by overall increased YAP binding, which however shifts more towards promoter regions. Analyses of 3D chromatin interactions at a set of 32 TEAD-target genes in TEADi-resistant cells reveals a reduction in distal enhancer (RE)–promoter contacts, despite reestablishment of YAP binding at distal enhancer elements. CRISPR-mediated deletion of several of these distal REs shows that they contribute to expression in parental cells, but are dispensable for gene activation in the resistant state. The authors then investigate FOSL1 (AP-1 component) chromatin occupancy, motivated by its previously established role in TEADi resistance (PMC11836325). They find that FOSL1 binding to chromatin is increased in both acute and resistant cells, again with a bias toward promoter regions, mirroring the YAP binding pattern. To identify potential upstream regulators of the transcriptional program in TEADi-resistant cells, the authors focus on transcription factors whose expression is either restored or increased in resistant cells or that are promoter specific, such as KLF4 and NFYA. ChIP-qPCR data shows that both factors have increased binding at a subset of TEAD-target genes in the

resistant cells. Notably, the authors suggest a hierarchical regulatory network where KLF4 regulates the expression of other TEAD-target genes, including FOSL1.

In summary, this study an interesting study, elegantly presented, featuring clear writing and effective data visualization. Overall it provides compelling evidence that in acquiring TEADi resistance the cells rearrange their cis-regulation at least partially through a mechanism that is less dependent on distal enhancers and more reliant on promoter-centred regulation. A switch from enhancer- to promoter-centric gene regulation has been previously described in association with SWI/SNF-inhibition (PMC11307202). However, the current manuscript unveils such a compensatory mechanism for a cell-type specific TF family (TEAD) in a disease setting, and provides mechanistic insights into the molecular rewiring that might underlie many resistance/adaptation processes in cancer. Moreover, the application of MCC to obtain mechanistic insight into a gene regulatory process is very well done and a great example of the power of this method that has rarely been used to such good effect. Additionally, although not yet approved for clinical use, TEADi are a promising therapeutic strategy for Hippo-deficient cancers, such as mesothelioma, and are currently in clinical trials. Taking this in consideration, this manuscript offers fundamental insights and relevant contributions towards understanding resistance mechanisms to TEAD inhibitors in YAP/TEAD-driven diseases. However, given the limited set of TEAD target genes the generality of some statements should be toned down. Moreover, the model developed from the results and the presentation/analyses of some data need revision. To fully support the publication of this manuscript following major and minor comments should be addressed

We thank the Reviewer for the positive and constructive evaluation of our manuscript. Below, we provide individual responses to all comments.

Major comments

1 – YAP/TEAD co-binding and global YAP binding at promoters in resistant cells
In the model depicted in Fig. 5E, the authors present YAP and TEAD co-binding in resistant cells. Is this truly the case? The authors should have the data (YAP ChIP-seq & TEAD Cut&RUN from Paul et al 2025) to clarify the binding dynamics of YAP and TEAD in resistant cells, including whether co-binding does occur, if both factors cooperate in resistant cells and how many of the restored or novel YAP peaks are TEAD-dependent, particularly within the 32 TEAD target genes. If co-binding cannot be demonstrated, the model in Fig. 5E should be revised accordingly. Also, why is KLF4 depicted at the resistant promoter, but not YAP, FOSL1 and NFYA?

We thank the Reviewer for these suggestions. As previously reported[10], YAP and TEAD1 chromatin occupancy is restored in resistant H226 cells, with substantial overlap between their binding sites (Paul et al., 2025; Fig. 4B–D). Consistent with this, our revised multi-omic analysis - performed after MCC peak calling and definition of RE-promoter connectivity (**Methods, Page 35-36, Lines 1056-1078**) - shows that both YAP and TEAD1 binding are restored on average at MCC-targeted promoters and MCC-linked distal REs in resistance (**Fig. 4c, d; Supplementary**

Fig. 6b, c). At the resolution of ChIP-seq and CUT&RUN, these data support broad co-occupancy of YAP and TEAD1, while not implying single-molecule co-binding.

To further clarify the selection of MCC-targeted genes, we now include pan-*TEAD1-4* and *YAP/TAZ* knockdown RNA-seq analyses, demonstrating that the majority of our MCC targeted genes that restore or gain expression in resistance are transcriptionally dependent on TEAD TFs and/or YAP/TAZ in parental cells (27 of the original 32; **Supplementary Fig. 2a; Page 8, Lines 211–215**). This confirms that the analyzed loci are enriched for *bona fide* TEAD/YAP targets.

Regarding the schematic in Fig. 5e, KLF4 is depicted at resistant promoters because our functional analyses identify it as the most consistent promoter-associated regulator in resistance. Genome-wide KLF4 ChIP-seq now shows that resistance-induced KLF4 binding is promoter-biased (21% vs. 7% for repressed KLF4 peaks; **Supplementary Fig. 5e, f**) and increased across MCC target promoters as well (**Fig. 4c**), but with enhanced occupancy at MCC-linked distal REs too (**Supplementary Fig. 6b, c**). In contrast, FOSL1 does not show a consistent increase at MCC target promoters (**Fig. 4c**), and NFYA does not exhibit resistance-induced expression or a comparable functional impact on target gene regulation to KLF4; accordingly, NFYA-related data have been removed in the revised manuscript to streamline data presentation.

We have updated **Fig. 5e** to reflect these findings, including restored YAP and TEAD1 binding at promoters in resistance, persistent loss of p300 and H3K27ac at both promoters and REs, promoter-specific recovery of H3K4me3, and resistance-induced KLF4 binding. Altogether, these analyses support that YAP and TEAD1 co-occupancy is broadly restored in resistance[10], while KLF4 displays promoter-biased binding that aligns most closely with promoter-centered transcriptional recovery at our MCC target genes.

Figure 5e

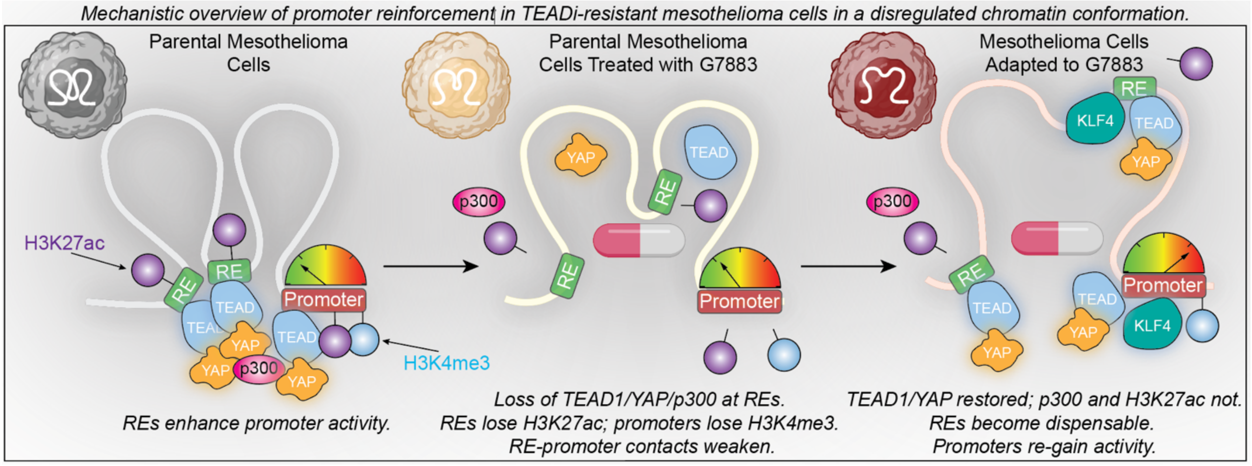

Figure legend: In parental mesothelioma cells, distal regulatory elements (REs) contact promoters and support gene expression. Acute treatment with the TEAD inhibitor GNE-7883 disrupts TEAD1/YAP binding, enhancer acetylation, and weakens RE-promoter interactions. In resistant cells, RE-promoter contacts are further weakened or lost, and distal REs lose functional influence despite continued transcription factor binding. In contrast, promoters regain activity marked by restored H3K4me3 and recruitment of resistance-associated transcription factors, including KLF4 but also TEAD1/YAP, enabling promoter-centered gene expression despite incomplete enhancer reactivation.

Additionally, it appears to me as if the shift towards promoter binding for YAP (as shown in Fig. 1E), is not reflected for those TEAD targets presented in Figs. 2, 3, and S2. Can the authors comment on this?

We agree with the Reviewer that the global promoter-biased shift or resistance-induced YAP binding (observed in Fig. 1f) is not consistently observed across the specific promoters shown in the original version in Figs. 2, 3, and Supplementary Fig.2. In the revised version, we have quantified YAP binding at the MCC-targeted promoters (+/-2kb around MCC-targeted gene TSSs), and we do, in fact, observe YAP restoration at individual gene promoters (**Supplementary Fig. 6b, c**), and, across MCC-linked REs (**Fig. 4d**), along with TEAD1 restoration. (described on Page 13, Lines 366-370). Therefore, we conclude that YAP and TEAD1 binding is restored at most MCC target promoters and linked REs - these observations are now part of our new schematic that we introduced above (**Fig. 5e**).

Finally, to evaluate condition-specific RE–promoter interactions involving TEAD motifs, the authors should present averaged MCC signal profiles centred on TEAD motifs across untreated, acute, and resistant states, similar to the analysis shown in Fig. 2C.

We thank the Reviewer for this suggestion. First, we note that we have now extended our analysis to a second TEAD-inhibitor–resistant mesothelioma model (MSTO-211H, referred to as MSTO), where we observe the same core phenomenon that we described in H226 cells, namely, a pronounced reduction of RE-P interactions in resistant cells compared to parental counterparts (**Fig. 2d-f; Supplementary Fig. 2b**). The reproducibility of this phenomenon across two independent models strengthens the central conclusion that RE-P connectivity diminishes during acquired resistance to TEAD inhibition.

In the revised manuscript, we have implemented an MCC peak-calling workflow, which produces an unbiased set of promoter-linked interactions/peaks (543 in H226 for 40 promoters and 1,838 in MSTO for 50; **Fig. 2b, e**). Rather than centering our analysis on predefined motifs, we now quantify MCC signals directly at these peak-called interaction sites across parental, acutely treated, and resistant states (**Fig. 2a, b, d, e, - see below**). This provides a more accurate and comprehensive assessment of RE-P communication than motif-centered aggregation, because it captures the full diversity of interaction types present in the MCC data in an unbiased manner.

Across all peak-called MCC interactions in both models, we observe a significant reduction in MCC signal in resistant cells (Wilcoxon signed-rank test, $p<0.05$), consistent with impaired RE-P communication (**Fig. 2b, e; Supplementary Fig. 2b**). These results extend and align with the datasets that we presented in the first submission and now rest on a more rigorous and unbiased quantification strategy. However, we note that while YAP binding is gained or restored at these locations in resistant H226 cells, it is not in resistant MSTO cells (**Fig. 2e**).

Together, the analyses performed in two independent mesothelioma models demonstrate that RE-P connectivity is reduced in drug-resistance, and the peak-based quantification provides a more robust framework for evaluating condition-specific differences.

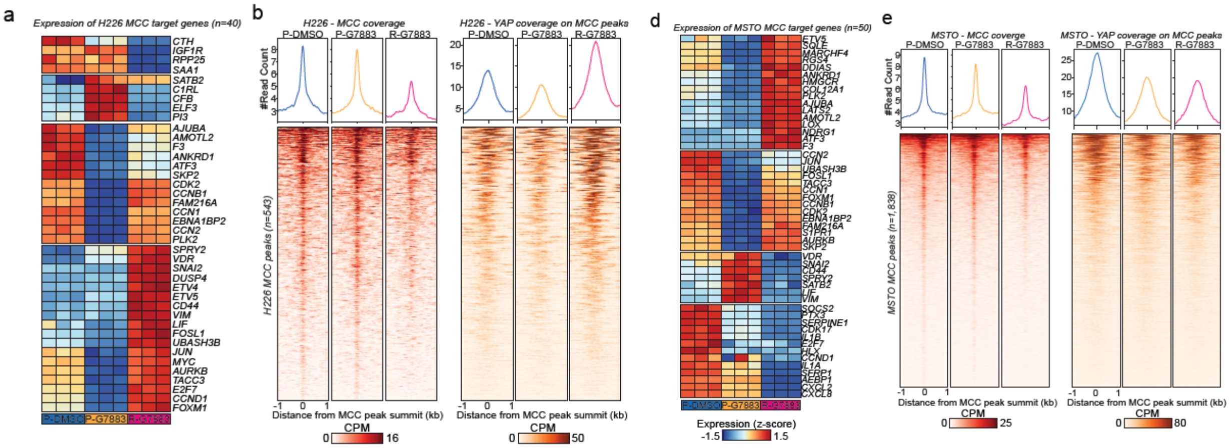

Figure legend: Heatmaps represents expression (z-score) of genes selected for MCC profiling in H226 cells (n=40) (**Fig. 2a**) or MSTO cells (n=50) (**Fig. 2d**) across parental (P-DMSO), acutely TEAD-inhibited (P-G7883), and resistant (R-G7883) conditions. Aggregated read-density plots and corresponding heatmaps depicting normalized MCC contact profiles (left) and YAP ChIP-seq coverage (right) centered on MCC peak summits in H226 (n=543; **Fig. 2b**) and in MSTO (n=1,838; **Fig. 2e**). # - indicates normalized read count (CPM).

2. FOSL1 binding at promoters and integration to the proposed model

The FOSL1 promoter-specific binding shown in Fig. 4A–C is convincing, yet this is not integrated in the proposed model in Fig. 5. Furthermore, FOSL1's functional relationship with YAP or KLF4 remains unclear, as is not shown if YAP-FOSL1-KLF4 act together to regulate the same subset of genes. In Fig. 4D, FOSL1 enrichment at TEAD-target promoters appears bimodally distributed in the resistant cells. Here, the authors should correlate FOSL1 levels at promoters with expression level of these target genes, as well (if possible) with YAP binding and RE-P interactions (e.g. What's the MCC read count/signal distribution between promoters and distal peaks in untreated vs. resistant cells?). This could strengthen the argument for a concerted shift towards promoter-based regulation achieved, especially at those promoters where YAP+FOSL1 bind.
Finally, I don't see the benefit of the TF-footprinting analysis as presented in Fig. 4E. The description in the results and methods lack clarity, statistical significance is not addressed, and the inconsistent symmetry across plots raises concerns about interpretability of the analysis. What is the contribution of this analysis to the overall conclusions?

We thank the Reviewer for raising these insightful points. In the revised manuscript, our expanded multi-omic framework - including MCC peak calling, promoter activity quantification, and integrated ChIP/CUT&RUN analyses - allowed us to more systematically assess FOSL1, YAP, and KLF4 behavior at MCC-linked RE-P interactions (**Fig. 4a-f, Supplementary Fig. 6a-d**).

First, to better clarify the bimodal distribution of FOSL1 occupancy observed in resistant cells (original **Fig. 4d**), we have now integrated several features of the regulatory landscape in these cells - including RNA-seq, promoter activity assessment[1], TF (FOSL1/KLF4/YAP/TEAD1) binding, histone modifications (H3K4me3, H3K27ac), p300, ATAC, and MCC signal. Collectively,

these data reveal that FOSL1 promoter enrichment does not consistently correlate with expression restoration, YAP binding, or RE-P interaction strength at our MCC-targeted genes. This is likely a reflection of the genes that were selected for capture based on their TEAD/YAP/TAZ dependence and resistance-associated expression behavior, rather than FOSL1 occupancy. As a result, FOSL1 gains are variable across these loci – on average trending towards being induced in resistance, but not predictive of transcriptional recovery and MCC looping interaction strength (**Fig. 4a-f, see below**).

Figure 4.

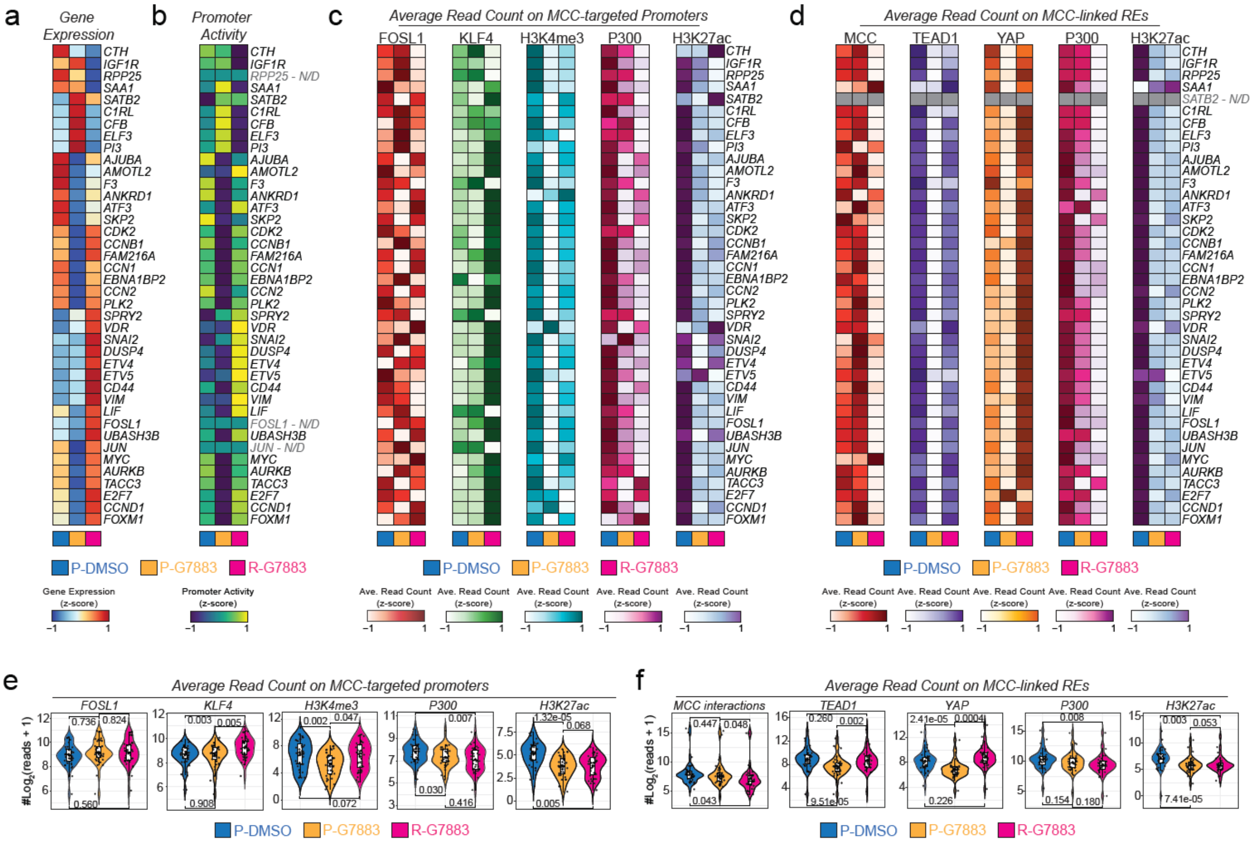

Figure legend: (**Fig. 4a, b**) RNA-seq expression and promoter activity (z-scores) for MCC targets (n = 40) across P-DMSO, P-G7883, and R-G7883. (**Fig. 4c, d**) Average normalized read counts (z-scores) at MCC-targeted promoters and MCC-linked distal REs for indicated TFs and histone marks. (**Fig. 4e, f**) Violin plots of normalized (# - CPM) read count distributions at promoters and distal REs (Wilcoxon test, p <0.05). N/D - not detected.

By contrast, our genome-wide KLF4 ChIP-seq shows that resistance-induced KLF4 peaks are promoter-biased, and KLF4 exhibits more consistent promoter binding gain across the genes we studied with MCC (**Fig. 4c, e**). Furthermore, KLF4 binding coincides with restored H3K4me3 and promoter activity (**Fig. 4a-c**), and its knockdown reduces proliferation and expression of multiple resistance-restored and -induced genes (**Fig. 5b-d**). In contrast, *FOSL1* knockdown has a weaker effect on our studied genes (**Supplementary Fig. 8c**), and we show that its expression requires the presence of KLF4 suggesting that KLF4 acts upstream of FOSL1 (**Fig. 5b**). These

data underlie our decision to depict KLF4, but not FOSL1 as the main promoter-acting factor at the MCC target loci in the model presented in Fig. 5e.

Additionally, incorporating FOSL1 and YAP tracks alongside MCC data in our genome browser plots (**Fig. 4g**) shows that RE-P interactions decrease in resistance even at sites where YAP or FOSL1 binding is restored. Combined with our MCC-targeted (**Supplementary Fig. 6b, c**) and global chromatin profiling data (**Supplementary Fig. 7a-e**), which shows widespread chromatin opening but lack of H3K27ac restoration reinforce the notion that enhancer function is impaired in resistance, and suggests that transcriptional rescue can occur through promoter-centered mechanisms. At promoters, YAP/TEAD1, FOSL1 and KLF4 can bind and act cooperatively at a subset of our example genes but this TF constellation does not uniformly apply to our MCC examples.

Finally, we concur that the TF-footprinting analysis did not substantially improve our mechanistic interpretation and have now removed these results from the updated manuscript.

3. Identification of key transcription factors that contribute to gene expression changes
While analysing TFs with increased or restored expression in resistant cells is a good starting point, a more informative approach would be to evaluate the individual contribution of each TF to gene expression changes across conditions. This could be done by integrating gene set enrichment analysis (GSEA) with transcription factor network analysis using expression data for each condition, through tools such as WebGestalt. Such analysis would help clarify the regulatory roles of the studied TFs and potentially uncover additional regulators that might warrant future investigation.

We thank the Reviewer for this suggestion and performed the recommended WebGestalt transcription-factor (TF) target analysis. This method infers upstream regulators based on over-representation of curated TF-target gene sets among our DEGs but does not perform motif scanning nor incorporate the TF’s own expression levels. Thus, it predicted SP-family regulators of resistance-induced expression, as well as other TFs, that are not differentially expressed in our system (e.g., the top hit PITX2, - **see below**). Interestingly, SP factors are well-known promoter-associated TFs that bind GC-box motifs, and their annotated target-gene sets substantially overlap with those of KLF-family TFs[9,10].

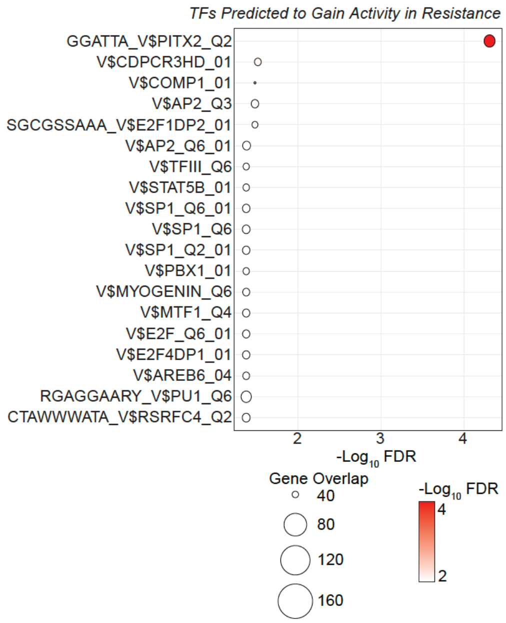

Figure legend: Differentially expressed genes (DEGs) were identified from RNA-seq using DESeq2 (FDR < 0.01, $Log_2FC>1$), and genes up-regulated in resistant versus parental cells were subjected to WebGestalt over-representation analysis (ORA) using the network_Transcription_Factor_target database (ChEA, ENCODE, TRANSFAC). The plot shows the top enriched TF-target sets whose experimentally annotated downstream genes are over-represented among resistance-induced DEGs. Dot size reflects the number of overlapping genes; color indicates $-\log_{10}$(FDR). Because this analysis relies on curated TF-target gene sets and does not incorporate TF expression, enriched SP-family factors reflect GC-box-associated promoter annotations in these databases.

Thus, the SP predictions are compatible with - but do not distinguish between - SP and KLF as regulators of gene expression in resistance. Importantly, the predicted SP-family TFs are not differentially expressed in resistance, whereas KLF4, which shares the same GC-rich binding preference, is differentially expressed and exhibits promoter-biased localization in resistant cells (**Supplementary Fig. 5e**).

To better address this suggestion and identify transcription factors that can more directly contribute to resistance-induced gene expression programs, instead, we implemented an integrative, expression-aware TF prioritization pipeline. First, we applied IPA (Ingenuity Pathway Analysis) upstream regulator analysis to the RNA-seq clusters (**Supplementary Fig. 5a**), then we focused on clusters with restored or gained expression in resistance (RNA-seq clusters 1, 4, and 5 - Fig. 1b). Second, we restricted these predictions to *bona fide* TFs and transcriptional regulators using the TFcheckpoint database (**Supplementary Fig. 5b**). Third, because upstream-regulator tools do not necessarily consider TF expression, we intersected this list with the 203 TFs whose expression is either restored or increased in resistance compared to parental cells, yielding 22 transcriptional regulators (**Supplementary Fig. 5c, - see below**).

Supplementary Fig. 5

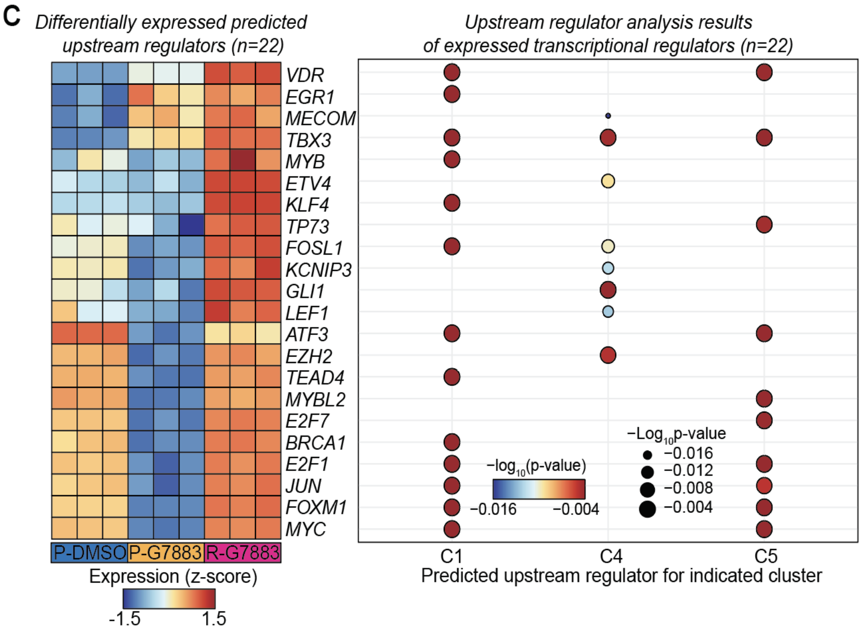

<u>**Figure legend:**</u> Heatmap depicting transcriptional regulators that were predicted by IPA upstream regulator analysis and whose expression was either restored or specifically increased in the resistant state (left panel). Dot plot summarizing IPA upstream regulator analysis results for the same factors (right panel; dot color and size represent $-\log_{10}$ enrichment p-values).

This refined set includes KLF4, FOSL1, and ETV4, the same TFs we perturb experimentally (**Fig. 5b; Supplementary Fig. 8c**). Our functional assays demonstrate that KLF4 is the dominant regulator of resistance-associated transcription on our TEAD/YAP target genes, with FOSL1 and ETV4 playing more modest roles. Overall, we believe that this integrative and experimentally validated approach provides a more biologically grounded framework for identifying TFs relevant to the resistant state than WebGestalt alone.

4. Generalization of the promoter switch model based on a limited selection of TEAD-target genes
The shift from distal regulatory elements to promoter-centric gene regulation is well supported for the subset of the TEAD target genes and nicely demonstrated through the CRISPR deletion experiments. However, as presented at the moment, the selection of the 32 MCC promoter targeted genes seems a bit arbitrary ("TEAD target genes that exhibit either restored or increase expression in resistant cells"). Why do the authors know these are direct TEAD targets? Why were these 32 selected vs. other genes from Clusters C1, C2 and C4 (if I am not mistaken)? The authors should give an explanation for that. Can they provide an alternative (control) set of genes that are differentially regulated (e.g. genes that do not recover from TEAD-inhibitor treatment?)

Furthermore, extrapolation from these 32 targets to a general "promoter switch" mechanism in resistant cells seems a bit premature, especially given a number of TEAD-target genes show only partial and ANKRD1 no switching

The study would be strengthened by providing bioinformatic support on why these 32 genes are representative of a general mechanism in the cells, or alternatively, why these 32 genes are well-suited to understand the mechanistic adaptation of YAP to the TEAD-inhibitor.

As of now, the 32 promoters appear to be cherry-picked without controls, and a sweeping statement as in l344/345 of the discussion ("Our work, leveraging MCC, demonstrates that most promoter elements in resistant cells exist within a dysregulated 3D chromatin configuration") is not supported.

We thank the Reviewer for raising these important points. In the revised manuscript, we now provide experimental evidence clarifying why most of the genes selected for MCC analysis are TEAD- and YAP/TAZ-regulated targets. Using *TEAD1-4* (pan-TEAD) knockdown and *YAP/TAZ* knockdown RNA-seq datasets, we show that 27 of the 32 MCC-targeted genes are transcriptionally dependent on TEAD and/or YAP/TAZ in parental H226 cells (**Supplementary Fig. 2a**). Thus, we can infer that the MCC capture regions include transcriptional targets of the TEAD-YAP/TAZ regulatory module, that are perturbed by the small molecule, G7883.

To address the request for appropriate controls, we have extended our capture probe set to include an alternative promoter set of genes whose expression is either unchanged or repressed in resistance (e.g., *CTH*, *RPP25*, *IGF1R*; n=8). Notably, while these control promoters also show loss of MCC-detected RE-promoter interactions, they typically exhibit weaker H3K4me3 recovery and weaker or no KLF4 promoter binding than the genes that recover or gain expression in resistance (**Fig. 4a-c**). This suggests that promoter-centered mechanisms are more restricted to a subset of genes in resistant cells, rather than a global response.

Additionally, to extend our understanding of this mechanism beyond the previously described 32 target loci, we have now incorporated a second TEAD inhibitor-resistant model (MSTO), as described above. In this system, we once again observe reduced RE-P interactions in resistance and validate via CRISPR perturbation that long-range RE function becomes dispensable (e.g., *FOSL1*, *TACC3*, *RGS4*), mirroring the H226 results (**Fig. 3d-f**). This independent model reinforces that weakened RE-promoter communication is a reproducible feature of TEAD-inhibitor resistance.

Finally, we have renamed the proposed phenomenon as “Promoter Reinforcement”, which we think more appropriately reflects the mechanism we describe. We have also toned down some of our conclusions to avoid implying that this behavior applies genome-wide; instead, emphasizing our observations at the specific TEAD/YAP-target genes interrogated in this study.

Together, we believe that these important additions address the Reviewer’s suggestions and strengthened our study.

5 - Control TEADi-resistant cells for genomic rearrangements at key loci:
From my understanding, the NCI-H226 TEADi-resistant cells were derived from the NCI-H226 cell line prior to the experimental setup with the acute and control treatments, as it is described in the methods that the resistant cell line was expanded using pooled populations of survivor cells. To confidently state that the observed changes in gene expression reflect drug resistance rather than spontaneous genomic alterations arising from longer cell culture passaging, the authors should provide basic controls such as chromosome counts or detection of copy number variations (e.g. arrayCGH) between the control cells and the resistant pool of cells, especially to rule out amplifications at key loci like KLF4 and YAP.

We thank the Reviewer for highlighting this important control. To exclude the possibility that the transcriptional changes observed in resistant cells arise from spontaneous genomic alterations rather than TEAD inhibitor-driven adaptation, we performed DNA-FISH assays targeting *YAP1* (**Supplementary Fig. 1g, see below**), *FOSL1* (**Supplementary Fig. 4a**), and *KLF4* (**Supplementary Fig. 5d, see below**) in parental and resistant H226 cells. These analyses reveal no detectable copy-number differences between parental and resistant H226 cells at any of these loci, indicating that the regulatory and expression changes we report in H226 are not attributable to gene amplification.

Supplementary Figures 1, 4 and 5.

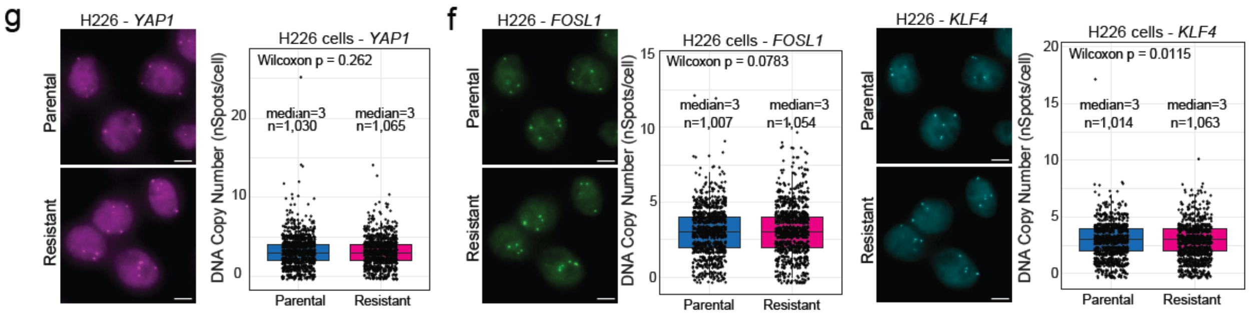

Figure legend: DNA FISH images and quantification by image analysis in H226 cells for the indicated genes. Wilcoxon signed-rank test was used to assess significant differences at p<0.001. Median of the distribution and the number of cells sampled in each group for data analysis are indicated.

For completeness, we performed *YAP1* DNA FISH in the MSTO model as well. Here, we do observe a *YAP1* copy-number gain in resistant cells (**Supplementary Fig. 1g, see below**). Importantly, despite this increase in *YAP1* dosage, MSTO resistant cells still display reduced RE-promoter interactions at our MCC loci, loss of distal RE function, but interestingly, no YAP restoration. Nonetheless, we detected promoter-centered transcriptional rescue at our studied genomic loci, consistent with the phenotype observed in H226 (**Fig. 3d, e**). Thus, even with higher

YAP1 copy number in MSTO, the qualitative regulatory features of resistance remain similar, supporting the conclusion that these adaptations are functional consequences of TEAD inhibition.

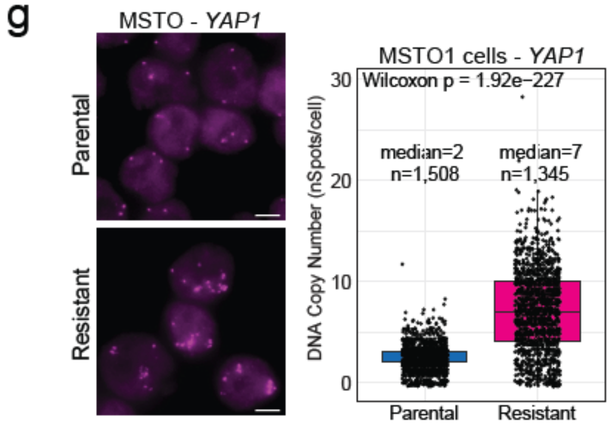

Figure legend: DNA FISH images and quantification by image analysis in MSTO cells for YAP1. Wilcoxon signed-rank test was used to assess significant differences at p<0.001. Median of the distribution and the number of cells sampled in each group for data analysis are indicated.

Together, these DNA-FISH results confirm that the regulatory changes we describe reflect drug-induced transcriptional and epigenetic adaptation mechanisms rather than the pure consequences of genomic drift. These results have been integrated into the revised manuscript: ***YAP* DNA FISH: Pages 7-8, Lines: 199-206; *FOSL1* DNA FISH: Page 11, Lines: 304-305; *KLF4* DNA FISH: Page 12, Lines: 337-338.**

Minor comments:

1 - The introduction would benefit from a brief explanation of the relevance of TEAD activity in mesothelioma and the rationale for using mesothelioma cell lines in this study.

We thank the Reviewer for this suggestion. We have now expanded the Introduction to briefly explain the relevance of TEAD activity in mesothelioma and our rationale for using mesothelioma cell line models in this study. These additions can be found on **Pages 3-4, Lines 77-84**: "*Malignant pleural mesothelioma is an aggressive cancer arising from pleural mesothelial cells, and epithelial-like cell layer lining the pleura. Mutations of upstream Hippo pathway regulators such as LATS2 or NF2 are frequently detected across pleural mesothelioma cells[33–35], culminating in high nuclear accumulation of YAP/TAZ and hyper-active transcriptional activity through its interaction with TEAD TFs. Thus, pharmacological regulation of the Hippo pathway bears therapeutic relevance to pleural mesothelioma and several other cancers, but it remains unclear how targeting this crucial pathway will affect the regulatory landscape of these cells.*".

2 - The authors should avoid overgeneralizing statements in cases where the data do not provide broad support and use more precise wording throughout the study.

We agree and we have toned down the wording throughout the manuscript to avoid overgeneralization and now use more precise language that better reflects our data – starting with our **Abstract** that states on **Page 2, Lines: 40-44**: "*Using base-pair-resolution Micro Capture-C at a defined set of TEAD target genes, we find that RE-promoter interactions are weakened or*

lost at many loci in resistant cells, even as promoter activity and gene expression recover in the context of partial epigenetic restoration.".

For instance:

• Lines 163–164: "biased towards promoters in resistant cells", should be TEADi-treated cells, as a similar bias is observed in acutely treated cells. – we have further edited this part of the text that can be found on **Page 7, Line 196-197** in the revised version: **"***These genomic annotation results show that TEADi- and resistance-induced YAP peaks are more enriched at promoter-proximal regions in both models.*".

• Line 236: "RE–P interactions" in TEAD-target genes – we have removed this sentence from the revised version.

• Line 345: Not all promoters show this feature, only the studied TEAD-promoters. – we have substantially revised the **Discussion** paying attention to all these details according to this suggestion. Example from **Discussion**: **Page 15, Lines 443-447**: "*By assessing promoter interaction profiles at 90 loci across two mesothelioma cell lines (H226: 40 promoters; MSTO: 50 promoters), we find that although TEADi-resistant cells regain YAP/TEAD1 binding genome-wide, this occurs within a dysregulated chromatin context in which many of our target promoters become physically uncoupled from their distal REs.*".

Additionally, we have introduced a new paragraph at the end of our **Discussion** to further emphasize some of the limitations of our study. **Page 16, Lines: 471-477**: "*In summary, our findings reveal a shift in gene regulatory logic in TEADi-resistant mesothelioma cells, in which promoter-centered regulation supports transcriptional resilience under therapeutic pressure. While this study focuses on a defined set of TEAD-responsive loci, it raises the possibility that promoter reinforcement contributes more broadly to adaptive gene regulatory responses in cancer. Determining the generality and therapeutic implications of this mechanism across additional drug-resistant cancer models will be an important direction for future work.*"

• Lines 361–363: The wording is misleading as it suggests that the proposed mechanism occurs genome-wide, instead the data supports this only for a specific subset of genes. – we have carefully revised this sentence in the revised manuscript as follows on: **Pages 15-16, Lines: 447-451**: "*While alterations in 3D genome organization in cancer are often interpreted through enhancer hijacking or TAD reorganization driven by structural variation and epigenetic reprogramming[50], our data demonstrate that the studied TEADi-sensitive genes can restore - and in many cases gain - expression without re-establishing RE-P connectivity in the resistant state.*"

Page 15, Lines 452-453: "*Instead, our results identify promoters as key regulatory units under these conditions in our gene set, supported by four lines of evidence:*"

3 – I suggest including a schematic model of untreated, acutely treated, and resistant cells as Fig. 1A, to illustrate the cellular model used throughout the manuscript and clarify the relation between cell conditions. “

We agree with the Reviewer’s suggestion and have now included a schematic in **Fig. 1a** that illustrates the relationship between the untreated, acutely treated, and resistant cell states, providing a clear overview of the cellular model used throughout the manuscript.

4 – If I understand correctly, the performed differential expression analysis involved a three-way comparison between untreated, acute, and resistant conditions. This should be better described in the Methods section, as we believe this is not immediately evident.

We have now further clarified the differential expression analysis in the Methods section, explicitly describing that it was performed as a three-way comparison between untreated (P-DMSO), acutely treated (P-G7883), and resistant (R-G7883) conditions. In the revised version, these details have been added on **Page 29, Lines 850-852**: “*Differentially expressed genes were identified based on a fold change cutoff of $Log_2FC>1$ and a false discovery rate cutoff of FDR<0.01 in a three-way comparison analysis strategy to retain all differential genes across conditions.*”.

5 - To further improve already strong visual coherence across Fig. panels, the authors could consider aligning the colour scheme used in Fig. S1A and S1D with that of the other Figures.

In the revised version, we substantially reorganized Supplementary Fig. 1 to improve visual coherence and consistency with the main figures. As part of this update, we aligned the color schemes and restructured the panels to better mirror the layout of Fig. 1. In the revised version, Supplementary Fig. 1 presents the MSTO RNA-seq and YAP ChIP-seq analyses in a similar format as Fig. 1 for H226. In doing so, we removed the original Supplementary Fig. 1a (PCA) and 1d (YAP histograms for each RNA-seq cluster), as they did not provide additional information beyond what is now presented in a more streamlined manner.

6 - The definition criteria of “TEAD-target genes” are not provided. Please specify the criteria used to identify TEAD targets either in the Methods section or within the main Results. (see Maj. Comment)

In the revised manuscript, we now clearly define the criteria used to identify TEAD-target genes. Specifically, we performed pan-TEAD knockdown and YAP/TAZ knockdown RNA-seq experiments in parental H226 cells and show that most genes included in our MCC panel (27 of the 32 included in the original study – highlighted in green on the heatmap in **Supplementary Fig. 2a**) exhibit reduced expression upon pan-TEAD and/or YAP/TAZ depletion. These datasets provide direct experimental evidence to the Reviewer’s comment. We have added these data in the **Results** section on **Page 8, Line 211-215**: “*Thus, we carried out high-resolution genome conformation mapping via MCC in our two models, and designed model-specific hybrid capture probes for promoters of TEAD target genes that were validated by YAP/TAZ or pan-TEAD perturbations in H226 parental cells (Supplementary Fig. 2a, right panel).*”.

7 – In addition to the metaplot of YAP coverage, I would like to see a heatmap of the ChIP-seq coverage at each of the 502 YAP peaks in Fig. 2B. This would help to assess how uniform the

recovery of the YAP signal is across peaks (as it appears from the bigwig track in the Fig.s), and probably better show the increased signal, as the authors write.
We thank the Reviewer for this suggestion. In the revised version, we have addressed this point using our newly implemented MCC peak-calling workflow, which provides an unbiased set of promoter-linked interacting genomic regions (peaks) for each promoter studied. We then plotted YAP ChIP-seq coverage at all MCC-called peaks in both H226 and MSTO (**Fig. 2b, e**). These heatmaps and histograms show that YAP signal is broadly restored across MCC-linked regions in resistant H226 cells, whereas YAP occupancy is not restored in the MSTO resistant state. This peak-based approach offers a comprehensive assessment of YAP signal across all RE-P interactions. In the revised version, we describe these results on **Page 8, Line 219-232**.

8 - Is the "repressed common FOSL1 peaks" plot in Fig. 4A correct? It appears identical to the "repressed acute FOSL1 peaks" plot?
We went back and carefully checked the underlying data, and the plot is correct. The overall pattern is very similar across the two peak groups, but the difference can be seen in the heatmaps when comparing the P-DMSO signal – signal intensity is higher in the "Common" group than in the "Acute" group (**now shown in Supplementary Fig. 4b**).

9 – The yaxis in FOSL1 ChIP in Fig. 4E is missing.
This panel belonged to the TF-footprinting analysis, which we have removed from the revised manuscript following the Reviewers' suggestion to keep the focus on the core findings.

10 - Is NFYA upregulated in the resistant state? The manuscript presents the expression profiles of all other TFs and uses expression levels as one of the criteria for selecting TFs to investigate. To maintain consistency, the authors should add NFYA expression across all conditions.
We thank the Reviewer for this question. Although NFYA binding was shown to be induced in the resistant cells at a few gene promoters, it does not exhibit resistance-induced expression. Since we have not expanded our NFYA datasets to the global level, we have removed the NFYA-related datasets from the revised manuscript. Instead, we expanded our analysis of KLF4 and other regulatory features of the MCC target loci, that provide better and more streamlined mechanistic insight into the promoter-centric compensatory mechanism seen in resistant cells.

11 - The discussion would benefit from a more detailed comparison between the mechanism proposed here and that described for SWI/SNF in PMC11307202 (lines 358–361). Expanding on both the similarities and differences would help to better contextualize the findings and clarify the novelty of the mechanism presented in this study.
We have now expanded the **Discussion** to include this comparison, which can be found on **Page 16, Lines 461-470**: "*Our observation that gene expression becomes uncoupled from distal RE activity at TEADi-sensitive loci parallels findings by Martin et al., who showed that acute inhibition of SWI/SNF chromatin remodeling complexes compromises enhancer activity while promoters recover accessibility through the EP400 complex[51]. Whereas their study captured an immediate compensatory response to chromatin remodeler loss, our work extends this concept by establishing promoter reinforcement as a stable regulatory feature in a drug-resistant state[51]. In*

addition, global loss of enhancer-promoter communication has been reported in the context of carcinogenesis[52], suggesting that promoter-centric regulatory behavior may represent a broader mode of transcriptional adaptation in cancer.".

12 – In addition to being a bit of an overstatement, based on the presented data (see Major Comment 3) the term "promoter switch" might not be the most accurate, as this to me implies a switch between two or more promoters.
We agree that the term "promoter switch" could be misleading. In the revised manuscript, we have replaced it with the term "Promoter Reinforcement" which better reflects the compensatory phenomenon we describe without implying a switch between alternative promoters.

References

1. Demircioğlu, D. *et al.* A Pan-cancer Transcriptome Analysis Reveals Pervasive Regulation through Alternative Promoters. *Cell* 178, 1465-1477.e17 (2019).
2. Roe, J.-S. et al. Enhancer Reprogramming Promotes Pancreatic Cancer Metastasis. Cell 170, 875-888.e20 (2017).
3. Hsu, E., Zemke, N. R. & Berk, A. J. Promoter-specific changes in initiation, elongation, and homeostasis of histone H3 acetylation during CBP/p300 inhibition. *eLife* 10, e63512 (2021).
4. Tropberger, P. et al. Regulation of Transcription through Acetylation of H3K122 on the Lateral Surface of the Histone Octamer. Cell 152, 859–872 (2013).
5. Lauberth, S. M. et al. H3K4me3 Interactions with TAF3 Regulate Preinitiation Complex Assembly and Selective Gene Activation. Cell 152, 1021–1036 (2013).
6. Zanconato, F. et al. Genome-wide association between YAP/TAZ/TEAD and AP-1 at enhancers drives oncogenic growth. *Nat. Cell Biol.* 17, 1218–1227 (2015).
7. Weinert, B. T. et al. Time-Resolved Analysis Reveals Rapid Dynamics and Broad Scope of the CBP/p300 Acetylome. Cell 174, 231-244.e12 (2018).
8. Barbosa, I. A. M. et al. Cancer lineage-specific regulation of YAP responsive elements revealed through large-scale functional epigenomic screens. *Nat. Commun.* 14, 3907 (2023).
9. Bernstein, B. E. et al. Genomic Maps and Comparative Analysis of Histone Modifications in Human and Mouse. Cell 120, 169–181 (2005).
10. Paul, S. et al. Cooperation between the Hippo and MAPK pathway activation drives acquired resistance to TEAD inhibition. Nat. Commun. 16, 1743 (2025).

NSMB-A50953A

We thank the Reviewers for their positive evaluation of our work. Their insights have been invaluable in refining our work and strengthening our conclusions. We have carefully revised the manuscript to address any remaining textual requests. Please find our point-by-point responses to the individual points below.

Reviewer #1 (Remarks to the Author):

The authors have addressed all my concerns. I recommend their interesting study for publication in NSMB.

We thank the Reviewer for their positive assessment and for recommending our manuscript for publication.

Reviewer #2 (Remarks to the Author):

The authors have added significant new data and analysis to their paper, strengthening the generality and clarity of their work. They have responded to all of the comments I raised in response to their original submission, and I recommend the revised version work for publication in its present form.

Reviewer #2 (Remarks on code availability):

As previously noted in response to the first submission.

We thank the Reviewer for their positive assessment and for recommending our manuscript for publication in its current form.

Reviewer #3:
Remarks to the Author:
The revised version of the manuscript is greatly improved, and the additional analyses and experiments strengthen the overall conclusion. The concerns raised in my review have been sufficiently addressed. I like the term “promoter reinforcement” for the observed phenomenon as well as the new Fig. 4a-d, which provides a lot of food for thought, also from a basic gene regulatory point of view.

However, there are two minor points that I want to raise and make the authors aware of.
1. The beginning of the results section describing Figs. 1-2 (up to ~line 250) are a really tough read, certainly stemming from describing the clustering and plots. I want to encourage the authors to improve readability as you might lose readers before the really exciting and strong part of the paper (to me at least) starts with the experiments in Figure 3 and the following Figs..

We appreciate the Reviewer's suggestion to improve readability of the results section. To address this, we have condensed our description of the RNA-Seq clusters (Lines 135-342).

2. Throughout the authors write of induced and repressed ChIP- and MCC-seq peaks, possibly trying to stay coherent with the gene expression terminology. I really struggled with making that suggestive link and I find it very inaccurate. A peak cannot be repressed. It can be gained or lost, or the signal at the peaks can be lower/weaker or higher/increased. I recommend to stay more descriptive/precise and revise this terminology.

We agree with the Reviewer that 'induced' and 'repressed' may imply functional expression changes rather than physical binding. We have updated the text to use the terms such as 'gained/increased' and lost/decreased' peaks to more accurately describe our ChIP-seq findings in the results (Lines 345-585; 1286-1295; 1319-1332), Methods and Figure Legends.
